# Digital sleep phenotype and wrist actigraphy in individuals at clinical high risk for psychosis and people with schizophrenia spectrum disorders: a systematic review and meta-analysis

**DOI:** 10.1136/bmjment-2024-301337

**Published:** 2025-02-10

**Authors:** Rosario Aronica, Edoardo Giuseppe Ostinelli, Charlotte Austin, Dominic Oliver, Philip McGuire, Paolo Brambilla, John Torous, Andrea Cipriani

**Affiliations:** 1 Department of Pathophysiology and Transplantation, University of Milan, Milano, Italy; 2 Department of Psychiatry, University of Oxford, Oxford, England, UK; 3 Department of Neurosciences and Mental Health, IRCCS Foundation Maggiore Policlinico Hospital, Milan, Lombardia, Italy; 4 Department of Psychiatry, University of Oxford, Oxford Precision Psychiatry Lab (OxPPL), Oxford, UK; 5 Warneford Hospital, Oxford Health NHS Foundation Trust, Oxford, England, UK; 6 NIHR Oxford Biomedical Research Centre, Oxford, England, UK; 7 Psychiatry, Beth Israel Deaconess Medical Center, Boston, Massachusetts, USA

**Keywords:** Sleep, Adult psychiatry, Schizophrenia & psychotic disorders, Data Interpretation, Statistical

## Abstract

**Aim:**

To identify sleep abnormalities in individuals at clinical high risk for psychosis (CHR-P) or with schizophrenia spectrum disorders (SSDs) compared with healthy controls (HCs) using wrist actigraphy, and to assess potential differences in the direction of effect with self-reported assessments of sleep.

**Methods:**

We conducted a systematic review of observational studies, with the search last updated on 29 April 2024. Primary outcome was total sleep time (TST), with secondary outcomes including time in bed (TIB), sleep latency, sleep efficiency, wake after sleep onset, nighttime awakenings and self-reported sleep quality. Random-effects pairwise meta-analyses were used to summarise the effects of each outcome.

**Results:**

Nineteen studies were included, with 18 contributing to the meta-analyses (202 CHR-P, 584 SSD, 582 HC). TST results were inconclusive for CHR-P (MD −4.88 min (95% CI −20.57 to 10.81)), while SSD participants showed an increase in TST compared with HC (MD 106.13 min (86.02 to 124.24)). Factors such as antipsychotic medications (pseudo-R²=88.14%), age (38.89%) and gender (26.29%) partially explained the heterogeneity between subgroups. Additionally, CHR-P individuals exhibited reduced sleep efficiency (MD −2.04% (−3.55 to 0.53)), whereas SSD participants had increased TIB (MD 121.58 min (88.16 to 155.00)) and sleep latency (MD 13.05 min (2.11 to 24.00)). The risk-of-bias assessment ranged from *some concerns* to *high risk*.

**Conclusions:**

Our analyses identified sleep abnormalities in CHR-P and SSD compared with placebo. However, observed heterogeneity and potential biases across studies may limit the interpretability of findings. These limitations underscore the need for standardised guidelines and more precise participant stratification.

**WHAT IS ALREADY KNOWN ON THIS TOPIC**

- Sleep abnormalities are common in individuals at clinical high risk for psychosis (CHR-P) and those with schizophrenia spectrum disorders (SSDs).
- Previous meta-analyses, primarily focused on polysomnography, have identified distinct sleep abnormalities across clinical stages of schizophrenia, from high-risk states to remitted schizophrenia.

**WHAT THIS STUDY ADDS**

- Our analysis of 18 studies—including 202 individuals with CHR-P, 584 with SSD and 582 healthy controls (HCs)—reveals that wrist actigraphy can identify sleep abnormalities in CHR-P and SSD compared with HCs.
- Sleep abnormalities across these diagnostic subgroups are influenced by participant characteristics and treatment with antipsychotic medications.
- Additionally, high heterogeneity was identified, likely influenced by the variability in wrist actigraphy devices, particularly in recording frequency, with more precise recordings providing more consistent outcome.

**HOW THIS STUDY MIGHT AFFECT RESEARCH, PRACTICE OR POLICY**

- Our study highlights the potential of wrist actigraphy in future research.
- As more data become available, this will enable validation of our findings and potentially aid identification of digital phenotypes of sleep across different subgroups of psychosis, which could inform detection strategies and putative treatment target.
- Additionally, this study underscores the need to standardise protocols and characteristics for wrist actigraphy devices to improve consistency and reliability of the data.

## Background

 Sleep is a complex physiological process driven by intrinsic neural network and regulated by circadian mechanisms, which entrain approximately to 24-hour cycles.^1^ These mechanisms regulate homeostatic functions and adapt to environmental and behavioural factors, such as light exposure and feeding patterns.^2^ Disruption to sleep cycles affect not only physical health^3^ but also mental health conditions.^4^ In fact, sleep abnormalities and insomnia are recognised as key diagnostic criteria in several clinical rating scales for psychiatric disorders.^5^

Sleep abnormalities impact the clinical trajectory of individuals at clinical high risk for psychosis (CHR-P) and with schizophrenia spectrum disorders (SSDs). They can trigger the onset of symptoms in CHR-P individuals,^6^ increase the risk of relapse in patients with SSD, exacerbate psychotic symptoms,^7^,^9^ impair quality of life^10^ and increase risk of suicide.^11^

Polysomnography (PSG) is the gold standard to measure sleep objectively.^12^ However, its limitations include low longitudinal reliability, high costs, interrater variability in the scoring and limited acceptability.^13^ By contrast, wearable technologies like actigraphy are easy to use, cheap and thus simplify collection of longitudinal data.^14^,^16^ The American Academy of Sleep Medicine has encouraged the use of wrist actigraphy to estimate sleep in adults with sleep disorders, circadian rhythm disorders and mental health conditions, including schizophrenia.^17^

Previous studies tried to summarise the evidence on sleep abnormalities in the context of CHR-P and SSD, but they focused on PSG studies.^18^,^20^ One meta-analysis by Meyer *et al*^21^ included studies using wrist actigraphy for sleep, but it accounted for just seven studies on people with remitted schizophrenia. Since the publication of this paper 4 years ago, new evidence has been produced.

## Objectives

This comprehensive systematic review and meta-analysis aims to identify sleep abnormalities in CHR-P and SSD compared with healthy controls (HCs), measured by at least 12 hours of continuous wrist actigraphy recording in observational studies. Secondarily, it aims to evaluate self-reported sleep assessment in the same studies to assess potential differences in the direction of effect.

## Study selection and analysis

The study protocol is available on Open Science Framework (osf.io/qjcdm).

We searched for any published and unpublished studies in PubMed, EMBASE, Medline, Cochrane Central Register of Controlled Trials, international trial registries (ClinicalTrials.gov, clinicalregistries.eu, WHO International Trial Registries) and OpenGrey until 29 April 2024. Reference lists of the included studies were also screened. No language restriction was applied. Details on the search string are provided in the online supplemental materials. We included observational studies using wrist actigraphy to assess sleep of people at CHR-P and with SSD, in comparison with HCs. People at CHR-P were diagnosed by using any established CHR-P psychometric instruments (ultra-high risk: Comprehensive Assessment of At-Risk Mental States (CAARMS), Structured Interview for Psychosis-Risk Syndromes / Scale of Prodromal Symptoms (SIPS/SOPS), Bonn Scale for the Assessment of Basic Symptoms in Psychosis (BSIP), Early Recognition Inventory Retrospective Assessment of Symptoms (EriRAOS); basic symptoms: Bonn Scale for the Assessment of Basic Symptoms (BSABS), Schizophrenia Proneness Instrument, Adult Version (SPI-A), Schizophrenia Proneness Instrument, Child and Youth Version (SPI-CY), or diagnostic criteria (Diagnostic and Statistical Manual of Mental Disorders, 5th Edition, Attenuated Psychosis Syndrome (DSM-5 APS)). Individuals at genetic risk for psychosis were also included. People with SSD were diagnosed by using any standard operationalised criteria, such as DSM-III, DSM-IV, DSM-5, ICD-10 and ICD-11. Studies on affective disorders with psychotic features and studies in which the comparator group were composed by individuals with sleep disorders (or other psychiatric diagnosis) were excluded. Reviews were not included, but their reference lists were screened. Relevant conference abstracts and published study protocols were also collected to seek the full publication or study data, when available.

Two researchers (RA, CA) independently screened and selected the studies, reviewed published and unpublished reports, extracted data from the included studies and assessed the risk of bias. Any discrepancies were double-checked and resolved by discussion with the other members of the team. For the outcomes of interest, researchers extracted mean values and standard deviations (or CIs and SEs when SD was unavailable). For three-arm studies (two non-controls and one control group), a combined mean was calculated by averaging the outcomes of the two non-control arms. This combined mean was then used for the analyses.^22^ For studies not reporting the SD value, it was calculated from the CI and sample size.^22^

### Outcome measures

The primary outcome was total sleep time (TST), as measured by wrist actigraphy. Secondary outcomes included other actigraphy measures of sleep continuity, such as time in bed (TIB), sleep latency, sleep efficiency, wakefulness after sleep onset (WASO) and nighttime awakenings (NA). Self-reported assessments of sleep (Pittsburgh Sleep Quality Index (PSQI) and Athens Insomnia Scale (AIS)) were also collected.

### Risk-of-bias assessment: ROBINS-E

We used the ROBINS-E tool^23^ to assess the risk of bias for our primary outcome across the included studies, ensuring consistent scoring despite expected heterogeneity in non-randomised study design.

### Statistical analysis

We conducted random-effect pairwise meta-analyses in CHR-P and SSD in comparison with HCs. We used mean differences or standardised mean differences based on whether study-specific outcomes were reported using a common metric or not. We used restricted maximum likelihood (REML) to estimate the between-studies variance τ^2^. Heterogeneity was assessed using I² and τ^2^ metrics, along with prediction intervals around the pooled effect size. We assessed the small-study effect bias through visual inspection of funnel plots and Egger’s test, which was applied when at least ten studies were included in a pairwise comparison.^22^

Subgroup analyses were conducted considering the duration of follow-up (less than 1 week, or 1 week and more), the epoch of the actigraphy device (less than 60 s, or equal and above 60 s) and the diagnostic subgroup (eg, chronic schizophrenia). Meta-regressions were performed based on the following set of preplanned covariates: publication year, percentage of individuals taking antipsychotic medications, age, gender, epoch, follow-up time, Positive and Negative Syndrome Scale (PANSS) total score severity, study sample and geography.

## Findings

**Figure 1:**
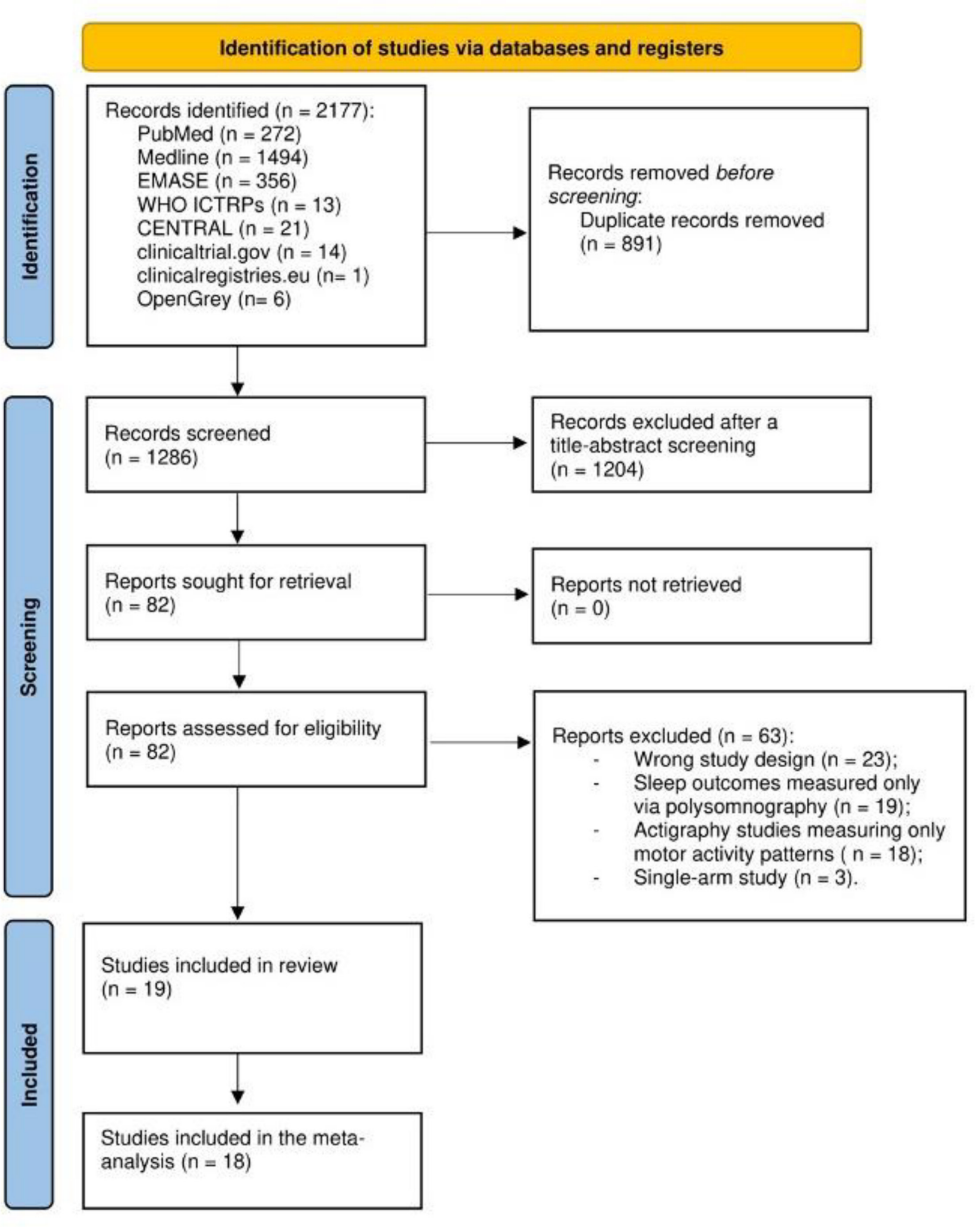
PRISMA (Preferred Reporting Items for Systematic Review and Meta-Analysis) 2020 flow diagram for systematic review.

The search yielded 2177 records (figure 1). After removing 891 duplicates, we screened 1286 records, with 82 records qualifying for the full-text assessment phase. Ultimately, 19 studies met the eligibility criteria. The average severity of psychotic symptoms for all included individuals was mild. Most of the studies used medical (non-consumer) devices,^24^ except for three studies that used the Actiwatch 2 by Philips, a device that can be classified as either consumer or non-consumer. The follow-up time ranged from 1 to 42 nights of continuous recordings. One study^25^ reported the outcomes as median values, and it did not contribute to the quantitative analysis. Therefore, 18 studies contributed to our meta-analyses, with a total of 202 participants at CHR-P, 584 participants with SSD and 582 HCs. The mean age of the participants at CHR-P was 20.7 years, with 51.3% being female; the mean age of the participants with SSD was 37.7 years, with 36.7% being female. In the HC groups, mean age was similar to the corresponding CHR-P/SSD group, but there were differences in terms of gender: with lower proportion of females (46.8%) in the control group for CHR-P; and higher proportion of females (48.8%) in the control group for SSD. Online supplemental table 1 summarises the characteristics of the studies included in the review.

In terms of risk of bias, 5 out of 18 studies (27.78%) had a *high risk* of bias, while the remaining 13 (72.22%) had *some concerns*. No studies were rated at *low risk* of bias (more details in online supplemental figures 1 and 2).

### Primary outcome: TST

For individuals at CHR-P (5 studies, 202 at-risk individuals and 140 HCs) no difference was found in TST (MD −4.88 min (95% CI −20.57 to 10.81)); however, individuals with SSD (13 studies, 574 participants and 442 HCs) showed an increased TST with a MD of 106.13 min (95% CI 86.02 to 124.24), even when the prediction interval was considered (44.59 to 167.68) (see figure 2a; more details in online supplemental figures 3-48).

**Figure 2:**
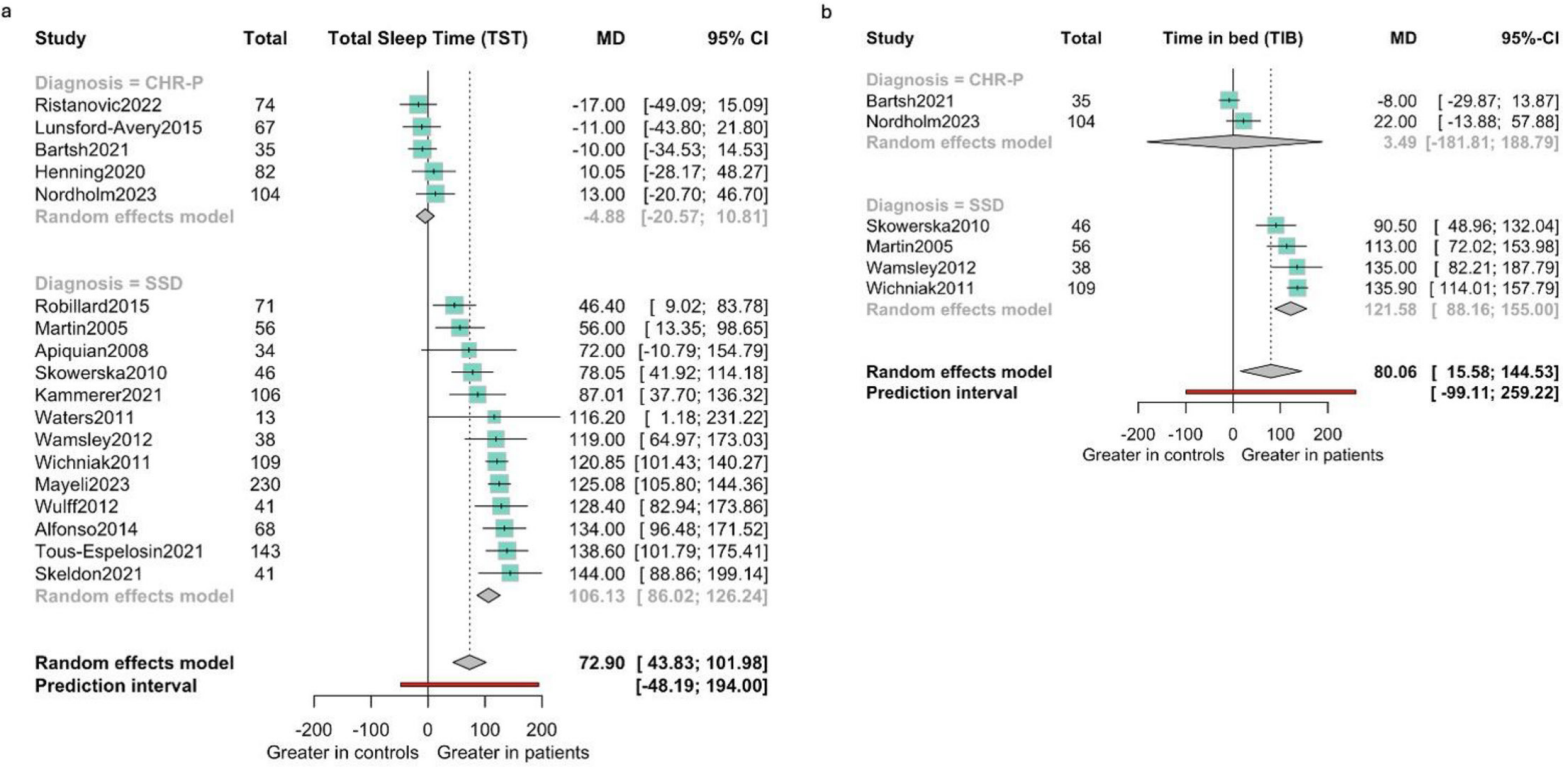
(a) Total sleep time by diagnostic subgroups (mean difference expressed in minutes). (b) Time in bed by diagnostic subgroups (mean difference expressed in minutes).

### Secondary outcomes

#### Time in bed

For individuals at CHR-P (2 studies, 87 CHR-P individuals and 52 HCs) no difference in time spent in bed (TIB) was found (MD 3.49 min (95% CI −181.81 to 188.79)). By contrast, individuals with SSD (4 studies, 145 SSD and 104 HCs) showed an increased TIB with an MD of 121.58 min ((95% CI 88.16 to 155.00); prediction interval (48.73 to 194.43)) (see figure 2b; more details in online supplemental figures 49-54).

#### Sleep latency

**Figure 3:**
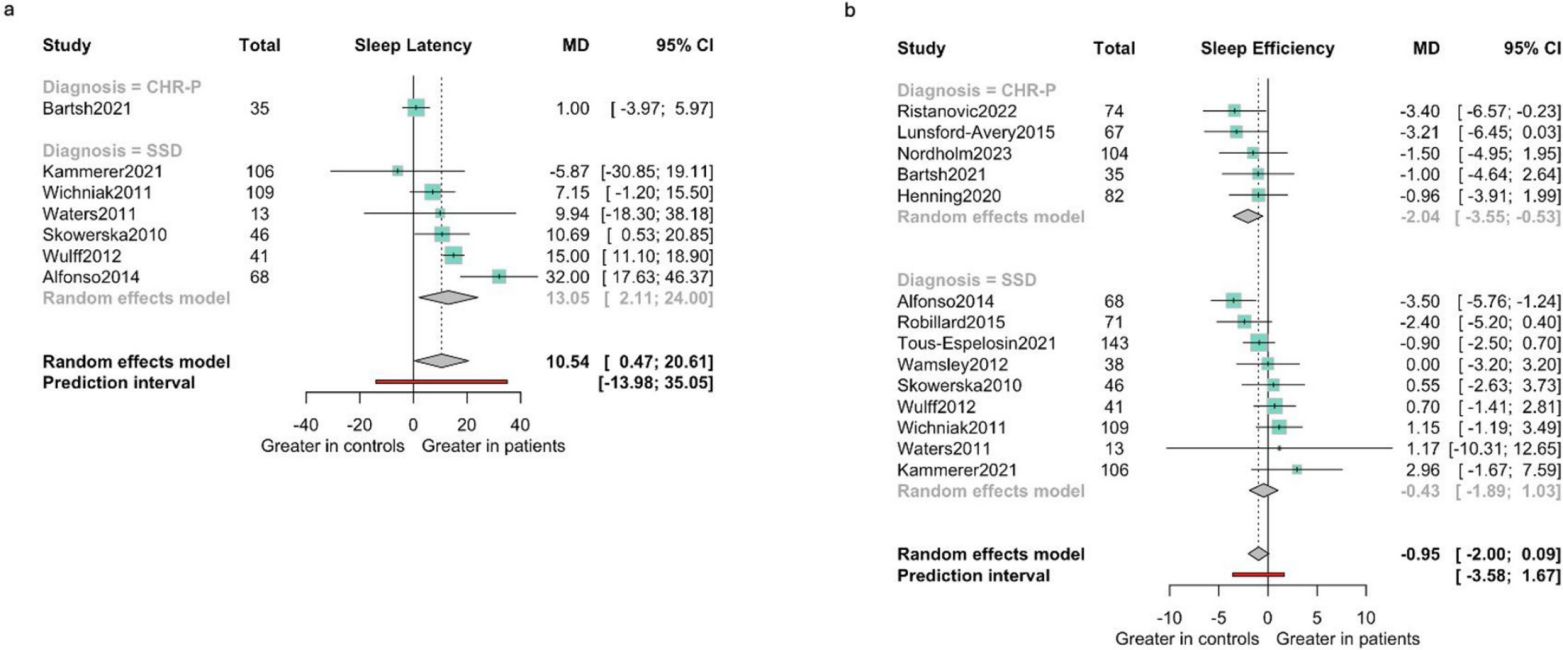
(a) Sleep latency by diagnostic subgroups (mean difference expressed in percentage). (b) Sleep efficiency by diagnostic subgroups (mean difference expressed in minutes).

Only one study was available for individuals at CHR-P (19 individuals CHR-P and 16 HCs), but it yielded inconclusive results (MD 1 min (95% CI −3.97 to 5.97). However, for individuals with SSD (6 studies, 240 SSD and 177 HCs), an increased sleep latency of 13.05 min was found (95% CI 2.11 to 24) with a prediction interval spanning from −9.45 to 35.55 (see figure 3a; more details in online supplemental figures 55-60).

#### Sleep efficiency

Individuals at CHR-P (5 studies, 202 CHR-P, 160 HCs) showed a reduced sleep efficiency by an MD of 2.04% ((95% CI −3.55 to 0.53); prediction interval (−4.41 to 0.33)). No difference was found for individuals with SSD (9 studies, 387 SSD and 248 HCs) (MD −0.43% (95% CI −1.89 to 1.03)) (see figure 3b; more details in online supplemental figures 61-66).

#### Wake after sleep onset (WASO)

**Figure 4:**
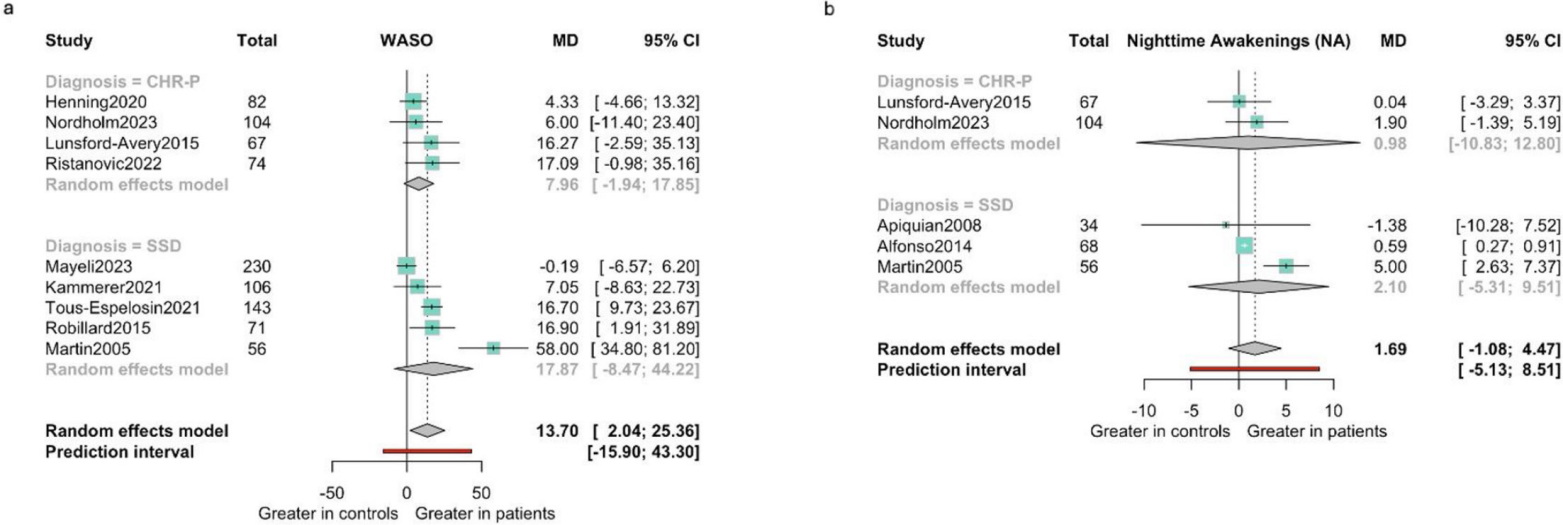
(a) Wake after sleep onset by diagnostic subgroups (mean difference expressed in minutes). (b) Nighttime awakenings by diagnostic subgroups (mean difference expressed in episodes).

There was no difference in terms of WASO for individuals at CHR-P (MD 7.96 min (95% CI −7 to 22.91); 4 studies, 183 CHR-P and 144 HCs), or for individuals with SSD (MD 17.87 min (95% CI −8.7 to 44.22); 5 studies, 360 SSD and 46 HCs) (see figure 4a; more details in online supplemental figures 67-72).

#### Nighttime awakenings (NA)

No differences in NA were found for individuals at CHR-P (2 studies, 104 CHR-P, 67 HCs) (MD 0.98 awakenings (95% CI −10.83 to 12.80)), or for individuals with SSD (3 studies, 79 SSD and 79 HCs) (MD 13.7 awakenings (95% CI −5.31 to 9.51)) (see figure 4b; more details in online supplemental figures 73-78).

#### Self-reported assessments of sleep (PSQI and AIS)

For individuals at CHR-P, data were available from only one study (41 CHR-P and 41 HCs), which showed inconclusive results (SMD 0.34 (95% CI −0.61 to 1.30)). We found no difference in the self-reported sleep assessments for individuals with SSD (3 studies, 60 SSD and 62 HCs), with an SMD of 0.28 (95% CI −0.43 to 0.99) (more details in online supplemental figures 79-84).

### Additional analyses for our primary outcome

We did not find differences in our sensitivity and meta-regression analyses within the two diagnostic subgroups. However, for people with SSD, subgroup analyses showed that studies using actigraphy with more frequent epochs (<60 s) have consistent results and minimal heterogeneity (MD 119.58 min (95% CI 97.61 to 141.55); prediction interval (85.36 to 153.81)). Finally, meta-regression analyses including all individuals (SSD and CHR-P) showed that the between-studies heterogeneity in TST can be explained by moderators, such as the percentage of individuals taking antipsychotics (pseudo-R^2^=88.14%; β=141.65), age (pseudo-R^2^=38.89%; β=3.26) and gender (pseudo-R^2^=26.29%; β=−217.49) (see online supplemental figures 9-11).

## Conclusion and clinical implications

We carried out a systematic review and meta-analysis to identify sleep abnormalities in individuals with SSD and those at CHR-P using wrist actigraphy.

We found that individuals with SSD reported an increase in TST compared with HCs, while those at CHR-P produced inconclusive results. We also showed that individuals with SSD spend more TIB and have increased sleep latency, whereas those at CHR-P exhibit reduced sleep efficiency.

The interplay between schizophrenia and sleep is complex.^26^ It has been suggested that dopaminergic pathways may play a crucial role in this relationship. Specifically, hyperactivity of D2 receptors in the striatum, which contributes to the positive symptoms of schizophrenia, may also influence wakefulness and lead to sleep abnormalities.^27^ This hypothesis is supported by murine models showing altered Rapid Eye Movement (REM)-like electrophysiological patterns,^27^ as well as human studies indicating that dopamine-antagonist medications can improve sleep.^28 29^ In our study, a meta-regression model revealed that the percentage of individuals taking antipsychotics greatly explained the variability in TST across individuals with SSD and CHR-P: a higher percentage of individuals receiving antipsychotic treatment was associated with greater TST. Although the literature suggests that approximately 40% of CHR-P receive antipsychotic treatment,^30^ only a small proportion of the individuals included in our meta-analyses was treated with antipsychotics. In contrast, consistent with the literature, the majority of SSD in the included studies was treated with one or more antipsychotics,^31^ which can impact sleep in various ways.^32^ A recent nationwide study^33^ found that antipsychotics, as a drug class, generally improve sleep abnormalities in patients with SSD, but they may also contribute to increase TST. However, the effects of antipsychotics on sleep vary depending on their specific affinities for dopaminergic, histaminergic, noradrenergic, muscarinic and cholinergic receptors.^34^ For example, clozapine is strongly associated with longer TST and improved sleep quality,^35^ whereas aripiprazole and quetiapine are linked to poorer sleep quality and sleep disruption.^36 37^ Unfortunately, only a few of the included studies provided information on the specific antipsychotic treatments taken by participants. As a result, we were unable to stratify the analysis by specific antipsychotic treatments or subgroup classifications (eg, typical vs atypical antipsychotics).

In addition to antipsychotic treatment, age and gender also contributed to the variability observed across CHR-P and SSD individuals. Mean age influenced TST, with older individuals experiencing a greater increase in TST compared with HCs. Since individuals at CHR-P are generally younger than SSD,^31^ they may exhibit milder clinical manifestations, including less severe sleep abnormalities.^20 31^ Higher percentage of women were associated with a reduced TST. It has been proposed that oestrogen and other hormones may play a role on the overall course of the disease, potentially dampening the severity of psychotic symptoms.^38^ This may partly explain why higher percentages of women were associated to less severe disruptions in TST in our study population, where the mean age was 34 years.

The agreement between actigraphy and PSG sleep continuity measures is known to decrease in populations with more fragmented and irregular sleep-wake patterns, such as SSD individuals. Therefore, the agreement between these methods in this population is typically defined as low to moderate.^39^ Our findings align with a previous meta-analysis of actigraphy studies in individuals with remitted schizophrenia,^21^ which reported increased TST, TIB and sleep latency. However, two meta-analyses of PSG studies produced opposite results for TST and TIB,^18 19^ highlighting potential discrepancies between these methodologies. Such differences may arise from the contexts in which data are collected: PSG is often conducted in clinical settings, where anxiety or unfamiliarity with the environment may alter sleep patterns,^40^ whereas actigraphy is recorded in naturalistic home settings over multiple days, potentially providing more representative estimates.^14^ Comparative studies of actigraphy and PSG have reported variable results. While many suggest comparable sleep estimates,^41^ others indicate that actigraphy may overestimate TST and underestimate sleep latency,^42^ or alternatively, underestimate TST and overestimate sleep latency.^43^ Including sleep diaries or self-report measures alongside actigraphy improves agreement with PSG, with some studies demonstrating non-inferiority.^41^ Despite these limitations, actigraphy remains a valuable tool for studying sleep in SSD due to its low cost, high acceptability and feasibility for longitudinal monitoring.^14 17^

For our secondary research question, we aimed to assess potential differences in the direction of effect between sleep continuity parameters, as assessed by wrist actigraphy, and self-reported assessments of sleep, the current gold standard. However, the limited number of studies providing self-reported data led to uncertain results. Previous research has compared self-reported sleep with actigraphy data in individuals with schizophrenia,^44^ revealing that sleep diaries often overestimate TST, sleep latency and sleep efficiency. However, no studies have specifically examined these discrepancies in individuals with CHR-P and SSD.

Our study has some limitations. According to the ROBINS-E tool, nearly 25% of the included studies were judged to be at high risk of bias, while the remaining 75% were assessed as having some concerns, with issues being identified mainly at domains 3 and 7. We found a *low risk* of bias in domain 2 (risk of bias arising from measurement of the exposure) and domain 6 (risk of bias arising from measurement of the outcome) across all studies. In domain 3 (risk of bias in the selection of participants), studies involving individuals at CHR-P consistently showed a *low risk* of bias, while those involving individuals with SSD raised *some concerns*. For the study by Mayeli *et al*,^45^ we identified a *high risk* of bias in domain 5 (risk of bias due to missing data). However, we acknowledged the authors’ efforts to mitigate this bias through transparent methods that provided justification for their missing data and included appropriate sensitivity analyses. As a result, we upgraded the overall risk of bias for this study to *some concerns*. To assess whether studies at higher risk of bias could influence the pooled effect sizes, we conducted sensitivity analyses excluding studies with an overall high risk of bias. These analyses showed no differences across all results, except for sleep latency, where the CIs crossed the line of no effect, rendering this result uncertain (see online supplemental figures 85-90).

While using actigraphy has its advantages,^14 17^ the lack of standardisation in study design and device selection introduces heterogeneity,^46^ which limits the interpretability of findings. In line with this, our analyses revealed that studies employing actigraphy devices with higher sampling frequencies (epochs <60 s) produced more consistent results and lower heterogeneity across all examined parameters. Furthermore, the use of varying algorithms for data processing across studies raises concerns about data comparability and external validity.^43^ A recent paper by Neishabouri *et al*^47^ highlighted that achieving shared interoperability of algorithms and consistency in data interpretation is essential for ensuring precise results and advancing the clinical application of wearable actigraphy, which is still limited.

Despite its limitations, our study highlights the potential of wrist actigraphy in future research.^17^ As more data become available, this will enable validation of our findings and potentially aid identification of digital phenotypes of sleep^48^ across different subgroups of psychosis, which could inform detection strategies and putative treatment target. Finally, we advocate for collaborative decision-making among patients, clinicians and regulatory agencies to establish standardised characteristics for actigraphy devices used in clinical settings and research.^46^ Future studies should address how intrinsic variability in sleep continuity parameters might affect the generalisation of results,^49^ enhancing the precision of outcome analyses and enabling accurate stratification of patient subgroups. Such personalised strategies will support the transition from a one-size-fits-all model to more individualised care.^50^

## supplementary material

10.1136/bmjment-2024-301337 online supplemental file 1

## Data Availability

All data relevant to the study are included in the article or uploaded as supplementary information.
