# Supplementary material for: Digital sleep phenotype and wrist actigraphy in individuals at clinical high risk for psychosis and people with schizophrenia spectrum disorders: a systematic review and meta-analysis
Source: BMJ Ment Health. 2025 Feb 10;28(1):e301337. doi: 10.1136/bmjment-2024-301337 (PMC11815447; doi:10.1136/bmjment-2024-301337)
Supplement: online supplemental file 1 [file bmjment-28-1-s001.pdf]

SUPPLEMENTAL METERIALS

SUPPLEMENTAL METERIALS 1

SEARCH STRING 3

LIST OF ARTICLES INCLUDED IN THE SYSTEMATIC REVIEW 3
- CHR-P 3
- SSD 3

TABLE OF INCLUDED ARTICLES 5

ROBINS-E 12

RESULTS TST 13
- All studies 13
  - Forest plot 13
  - Funnel plot 14
  - Outliers 15
  - Meta-regression 19
  - Subgroup analyses 29
  - Sensitive analyses 33
- SSD studies 37
  - Forest plot 37
  - Funnel plot 38
  - Outliers 39
  - Meta-regressions 42
  - Subgroup analyses 51
- CHR-P studies 55
  - Forest Plot 55
  - Funnel plot 56
  - Outliers 56
  - Meta-regressions 58

RESULTS TIB 62
- Forest plot – all studies 62
- Funnel plot 63
- Eggers' test of the intercept 63
- Sensitivity analyses 63
  - SSD studies 63
- CHR-P studies 65
  - Forest plot 65
- Outliers and heterogeneity 65
- Influence analyses 67
  - Baujat plot 67
  - Influence diagnostics 67

RESULTS SLEEP LATENCY 68
- Forest plot – all studies 68
- Funnel plot 70
  - Eggers' test of the intercept 70
- SSD studies 71
  - Forest plot 71
- CHR-P studies 72

Forest plot 72
Outliers and heterogeneity 72
Influence analyses 74
Baujat plot 74
Influence diagnostics 74

RESULTS SLEEP EFFICIENCY 75
Forest plot – all studies 75
Funnel plot 76
Linear regression test of funnel plot asymmetry 76
Eggers' test of the intercept 77
SSD studies 77
Forest plot 77
CHR-P studies 78
Forest plot 78
Outliers and heterogeneity 79
Influence analyses 79
Baujat plot 79
Influence diagnostics 79

RESULTS WASO 80
Forest plot – all studies 80
Funnel plot 81
Eggers' test of the intercept 82
Sensitivity analyses 82
SSD studies 82
CHR-P studies 83
Outliers and heterogeneity 84
Influence analyses 85
Baujat plot 85
Influence diagnostics 86

RESULTS Nighttime Awakenings 87
Forest plot – all studies 87
Funnel plot 88
Eggers' test of the intercept 88
SSD studies 89
Forest plot 89
CHR-P studies 89
Forest plot 89
Outliers and heterogeneity 90
Influence analyses 92
Baujat plot 92
Influence diagnostics 93

QUALITY OF SLEEP – SMD PSQI and AIS 93
Forest plot – all studies 93
Funnel plot 94
Eggers' test 95
Baujat Plot 96
Influence analyses 96
SSD studies 97
CHR-P 98

PRISMA 2020 Checklists 98
PRISMA 2020 Main Checklist 98
PRISMA Abstract Checklist 102

Sensitivity analyses accounting for RoB (excluding studies with a high risk of bias) **103**
Total sleep time 103
Time in bed 105
Sleep Efficiency 106
Sleep Latency 108
Wakefulness After Sleep Onset 109
Nighttime awakenings 110

SEARCH STRING

The query string used for the search was based on the combinations of the following terms: ((psychotic disorders[MeSH Terms]) OR (psychosis[MeSH Terms]) OR (schizophrenia[MeSH Terms]) OR high-risk mental stat* OR high-risk psycho* OR high risk psycho* OR ultra-high-risk psychos* OR at-risk mental state OR psychosis proneness OR liability for schizophrenia OR clinical-high-risk psychosis OR CHR-P OR CHR OR UHR OR first-episode psychosis OR FEP OR schizotyp*) AND (sleep* [All Fields] OR insomnia [All Fields]) AND (("actigraph*"[All Fields]) OR ("actimetr*"[All Fields]) OR ("acceleromet*"[All Fields]) OR ("wearables"[All Fields]) OR (smartwatch[All Fields])).

LIST OF ARTICLES INCLUDED IN THE SYSTEMATIC REVIEW

CHR-P

1. Ullrich Bartsch, Laura J Corbin, Charlotte Hellmich, Michelle Taylor, Kayleigh E Easey, Claire Durant, Hugh M Marston, Nicholas J Timpson, Matthew W Jones, Schizophrenia-associated variation at *ZNF804A* correlates with altered experience-dependent dynamics of sleep slow waves and spindles in healthy young adults, *Sleep*, Volume 44, Issue 12, December 2021, zsab191, https://doi.org/10.1093/sleep/zsab191
2. Hennig T, Schlier B, Lincoln TM. Sleep and psychotic symptoms: An actigraphy and diary study with young adults with low and elevated psychosis proneness. Schizophr Res. 2020 Jul;221:12-19. doi: 10.1016/j.schres.2019.09.012. Epub 2019 Nov 30. PMID: 31796308.
3. Lunsford-Avery JR, LeBourgeois MK, Gupta T, Mittal VA. Actigraphic-measured sleep disturbance predicts increased positive symptoms in adolescents at ultra high-risk for psychosis: A longitudinal study. Schizophr Res. 2015 May;164(1-3):15-20. doi: 10.1016/j.schres.2015.03.013. Epub 2015 Mar 26. PMID: 25818627; PMCID: PMC4409558.
4. Nordholm D, Jensen MA, Glenthøj LB, Kristensen TD, Wenneberg C, Garde AH, Nordentoft M. Sleep disturbances and the association with attenuated psychotic symptoms in individuals at ultra high-risk of psychosis. J Psychiatr Res. 2023 Feb;158:143-149. doi: 10.1016/j.jpsychires.2022.12.041. Epub 2022 Dec 23. PMID: 36584492.
5. Ristanovic I, Haase CM, Lunsford-Avery JR, Mittal VA. The relationship between stress responding in family context and stress sensitivity with sleep dysfunction in individuals at clinical high-risk for psychosis. J Psychiatr Res. 2022 May;149:194-200. doi: 10.1016/j.jpsychires.2022.02.038. Epub 2022 Mar 2. PMID: 35287048; PMCID: PMC9176292.

SSD

6. Afonso P, Figueira ML, Paiva T. Sleep-promoting action of the endogenous melatonin in schizophrenia compared to healthy controls. Int J Psychiatry Clin Pract. 2011 Nov;15(4):311-5. doi: 10.3109/13651501.2011.605954. Epub 2011 Aug 28. PMID: 22122006.

7. Apiquian, R., Fresán, A., Muñoz-Delgado, J., Kiang, M., Ulloa, R. E., & Kapur, S. (2008). Variations of rest-activity rhythm and sleep-wake in schizophrenic patients versus healthy subjects: An actigraphic comparative study. Biological Rhythm Research, 39(1), 69–78. https://doi.org/10.1080/09291010701318253
8. Kammerer MK, Mehl S, Ludwig L, Lincoln TM. Sleep and circadian rhythm disruption predict persecutory symptom severity in day-to-day life: A combined actigraphy and experience sampling study. J Abnorm Psychol. 2021 Jan;130(1):78-88. doi: 10.1037/abn0000645. Epub 2020 Nov 19. PMID: 33211503.
9. Lauerma H, Niskanen L, Lehtinen I, Holmström R. Abnormal lateralization of motor activity during sleep in schizophrenia. Schizophr Res. 1994 Dec;14(1):65-71. doi: 10.1016/0920-9964(94)90010-8. PMID: 7893623.
10. Martin JL, Jeste DV, Ancoli-Israel S. Older schizophrenia patients have more disrupted sleep and circadian rhythms than age-matched comparison subjects. J Psychiatr Res. 2005 May;39(3):251-9. doi: 10.1016/j.jpsychires.2004.08.011. PMID: 15725423.
11. Mayeli A, LaGoy AD, Smagula SF, Wilson JD, Zarbo C, Rocchetti M, Starace F, Zamparini M, Casiraghi L, Calza S, Rota M, D'Agostino A, de Girolamo G; DiAPAson Consortium; Ferrarelli F. Shared and distinct abnormalities in sleep-wake patterns and their relationship with the negative symptoms of Schizophrenia Spectrum Disorder patients. Mol Psychiatry. 2023 May;28(5):2049-2057. doi: 10.1038/s41380-023-02050-x. Epub 2023 Apr 14. PMID: 37055512.
12. Robillard R, Hermens DF, Naismith SL, White D, Rogers NL, Ip TK, Mullin SJ, Alvares GA, Guastella AJ, Smith KL, Rong Y, Whitwell B, Southan J, Glozier N, Scott EM, Hickie IB. Ambulatory sleep-wake patterns and variability in young people with emerging mental disorders. J Psychiatry Neurosci. 2015 Jan;40(1):28-37. doi: 10.1503/jpn.130247. PMID: 25203899; PMCID: PMC4275328.
13. Skeldon AC, Dijk DJ, Meyer N, Wulff K. Extracting Circadian and Sleep Parameters from Longitudinal Data in Schizophrenia for the Design of Pragmatic Light Interventions. Schizophr Bull. 2022 Mar 1;48(2):447-456. doi: 10.1093/schbul/sbab124. PMID: 34757401; PMCID: PMC8886588.
14. Skowerska A, Wichniak A, Skalski M. Zaburzenia snu i rytmu okołodobowego w schizofrenii [Sleep and circadian rhythm disturbances in schizophrenia]. Psychiatr Pol. 2010 Sep-Oct;44(5):621-31. Polish. PMID: 21452499.
15. Tous-Espelosin M, de Azua SR, Iriarte-Yoller N, MartínezAguirre-Betolaza A, Sanchez PM, Corres P, Arratibel-Imaz I, Sampedro A, Peña J, Maldonado-Martín S. Clinical, physical, physiological, and cardiovascular risk patterns of adults with schizophrenia: CORTEX-SP study: Characterization of adults with schizophrenia. Psychiatry Res. 2021 Jan;295:113580. doi: 10.1016/j.psychres.2020.113580. Epub 2020 Nov 18. PMID: 33246589.
16. Wamsley EJ, Tucker MA, Shinn AK, Ono KE, McKinley SK, Ely AV, Goff DC, Stickgold R, Manoach DS. Reduced sleep spindles and spindle coherence in schizophrenia: mechanisms of impaired memory consolidation? Biol Psychiatry. 2012 Jan 15;71(2):154-61. doi: 10.1016/j.biopsych.2011.08.008. Epub 2011 Oct 2. PMID: 21967958; PMCID: PMC3561714.
17. Waters F, Sinclair C, Rock D, Jablensky A, Foster RG, Wulff K. Daily variations in sleep-wake patterns and severity of psychopathology: a pilot study in community-dwelling individuals with chronic schizophrenia. Psychiatry Res. 2011 May 15;187(1-2):304-6. doi: 10.1016/j.psychres.2011.01.006. Epub 2011 Jan 26. PMID: 21272939.
18. Wichniak A, Skowerska A, Chojnacka-Wójtowicz J, Tafliński T, Wierzbicka A, Jernajczyk W, Jarema M. Actigraphic monitoring of activity and rest in schizophrenic patients treated with olanzapine or risperidone. J Psychiatr Res. 2011 Oct;45(10):1381-6. doi: 10.1016/j.jpsychires.2011.05.009. Epub 2011 Jun 15. PMID: 21679968.
19. Wulff K, Dijk DJ, Middleton B, Foster RG, Joyce EM. Sleep and circadian rhythm disruption in schizophrenia. Br J Psychiatry. 2012 Apr;200(4):308-16. doi: 10.1192/bjp.bp.111.096321. Epub 2011 Dec 22. PMID: 22194182; PMCID: PMC3317037.

TABLE OF INCLUDED ARTICLES

Table 1. Table of the included studies, organised by diagnostic subgroups.

Study ID	Diagnosis	Study sample, N (Men/Women, age ± SD)	Symptoms severity (Scale used, mean score ± SD)	Medications for individuals with exposure	Actigraphic device	Epochs	Follow-up time (max)	Main results
Alfonso et al., 2014	SSD	Individuals with exposure:<br>34 (22/12, 33.88 ± 8.6);<br><br>HC: 34(19/15, 34.7 ± 8.3).	PANSS General, 66.9 ± 17.1;<br><br>PANSS Positive subscale,<br>13.6 ± 5;<br><br>PANSS Negative subscale,<br>17.2 ± 6.2.	100% on antipsychotics	SOMNOwatch	1 second	7 days	SSD patients have increased **total sleep time** (mean 09:57h, SD 01:43h) compared to HC (07:26 h, SD 00:43 min);<br><br>SSD patients have increased **sleep latency** (mean 47 min, SD 42 min) compared to HC (mean 15 mins, SD 9 mins);<br><br>SSD patients have decreased **sleep efficiency** (92.8%, SD 6.01%), compared to HC (96.3%, SD 3);<br><br>SSD patients have increased **nighttime awakenings** (mean 1.28, SD 0.86) compared to HC (mean 0.69, SD 0.41).
Apiquian et al., 2008	SSD	Individuals with exposure:<br>20 (10/10, 28.5 ± 7.2);<br><br>HC:<br>20 (6/14, 30.3±5.8).	NR	75% (9) on atypical antipsychotics; 25% (3) on typical antipsychotics.	Actiwatch-16, Mini Mitter Company, Bend, OR	60 seconds	6 days	SSD patients have increased **total sleep time** (mean 06:40 h, SD 01:41h) compared to HC (05:57 h, SD 02:09h);<br><br>No difference in **nighttime awakenings** the two groups.

Kammerer et al., 2021	SSD	Individuals with exposure: 67(38/29, 38.0 ±12.3);<br><br>HC: 39(21/18 37.9±13.8).	PANSS Total, 33.87 ± 6.79;<br><br>PANSS Positive, 16.66 ± 3.96;<br><br>PANSS Negative, 15.78 ± 4.73.	84% on antipsychotics	NR	NR	6 days	SSD patients have increased **total sleep time** (mean 08:22h, SD 02:35h) compared to HC (06:55h, SD 01:42h);<br><br>SSD patients have decreased **sleep latency** (36.16mins, SD 49.17mins) compared to HC (42.03mins, SD 70.2mins);<br><br>SSD patients have increased **wake after sleep onset** (49.53mins, SD 44.06mins) compared to HC (42.48mins, SD 36.97mins);<br><br>SSD patients have increased **sleep efficiency** (82.82%, SD 11.34%) compared to HC (79.86%, SD 11.96%).
Lauerma et al., 1994	SSD	Individuals with exposure: 13(5/8, 41.5±10.5);<br><br>HC: 17 (10/7, 29.5 ±8.0).	NR	100% on antipsychotics	Gaehwiler Electronics, Switzerland	NR	1 day	SSD patients have increased **total sleep time** (mean 08:42h, SD 00:38h) compared to HC (06:52h, SD 00:26h);<br><br>No difference in **nighttime awakenings** between the two groups.
Martin et al., 2005	SSD: Chronic Schizophrenia	Individuals with exposure: 28(14/14,58.3 ±9.8);<br><br>HC:28(14/14,57.3 ±9.2).	NR	NR	Actillume wrist actigraph	60 seconds	3 days	No difference in **total sleep time** between the two groups;<br><br>SSD patients spent more **time in bed** (mean 09:46h, SD 00:96h) compared to HC (07:53h, SD 00:55h);<br><br>SSD patients have increased **wake after sleep onset** (80mins, SD 60mins) compared to HC (22mins, SD 18mins);

								SSD patients have a increased **number of nighttime awakenings** (8.00, SD 5.00) compared to HC (3.00, SD 4.00).
Mayeli et al. 2023	SSD	Individuals with exposure:<br>122 (77/45);<br><br>HC: 108 (66/42, 41.5 ± 10.1).	BNSS (Inpatient), 24.1 ± 14.9;<br><br>BNSS (Outpatient), 17.2 ± 13.9	Inpatients: 100% on antipsychotics, 27.9% on mood stabilizers, 32.3% on antidepressants, 69.1% on benzodiazepines;<br><br>Outpatients: 98.1% on antipsychotics, 9.3% on mood stabilizers, 40.7% on antidepressants, 35.2% on benzodiazepines.	ActiGraph GT9X-Pensacola	NR	7 days	SSD patients have increased **total sleep time** (494.38 min, SD 87.89 min) compared to HC (369.3 min, 60.07 min);<br><br>No difference in **nighttime awakenings** between the two groups.
Robillard et al., 2015	SSD	Individuals with exposure:<br>30(22.5±5.1);<br><br>HC: 41(25.3±5.8).	NR	70% on antipsychotics	Actiwatch-64/L/2, Philips Respironics	60 Seconds	22 days	SSD patients have increased **total sleep time** (08:07h, SD 01:31h) compared to HC HC (07:21h, SD 00:53h)
Skeldon et al., 2021	SSD	Individuals with exposure:	NR	100% on antipsychotics	Actiwatch-L (Cambridge Neurotechnol	120 seconds	42 days	SSD patients have increased **total sleep time** (mean 07:30h SD 01:18h) compared to HC (5:06h, SD 1:40h).

		20(15/5, 38.8 ±8.6);<br><br>HC: 21(13/8, 37.5±9.6).			ogy Ltd, Cambridge, UK			
Skowerska et al., 2010	SSD	Individuals with exposure: 23 (40.5 ±13.7);<br><br>HC: 23 (40.5 ± 13.7).	PANSS Total, 24.2 ± 5.4	100% on antipsychotics	NR	NR	NR	SSD patients have increased **total sleep time** (mean 07:49h, SD 01:09h) compared to HC (06:31h, SD 00:55h);<br><br>SSD patients spent more **time in bed** (09:11, SD 01:24) compared to HC (07:40 SD 00:56h);<br><br>SSD have increased **sleep latency** (32.95mins, SD 22.09mins) compared to HC (22.26mins, SD 11.41mins);<br><br>No difference in **efficiency** between the two groups.
Tous-Espelosin et al., 2021	SSD	Individuals with exposure: 126(105/21,41.6± 10.3);<br><br>HC: 30(12/18, 40.00±9.0).	NR	100% on antipsychotics	ActiGraph GT3X+, Pensacola, Florida, USA	60 seconds	8 days	No difference in **total sleep time, wake after sleep onset and efficiency** between the two groups.
Wamsley et al., 2012	SSD: chronic schizophrenia	Individuals with exposure: 21(17/4, 34±9);<br><br>HC: 17(14/3, 36±7).	PANSS Total, 28 ± 10	100% on antipsychotics	The Mini-Mitter Actiwatch	15 seconds	5 days	No difference in **total sleep time, time in bed and efficiency** between the two groups.

Waters et al., 2011	SSD: chronic schizophrenia	Individuals with exposure: 6 (5/1, 44.33± 4.96);<br><br>HC: 7(4/3, 42.71±7.52).	BPRS, 38.33 ± 8.04	100% on antipsychotics (Clozapine)	Actiwatch 2 (Philips Respironics)	60 seconds	28 days	SSD patients have increased **total sleep time** (mean 09:46h, SD 02:07h) compared to HC (07:50h, SD 01:11h)
Wichniak et al., 2011	SSD	Individuals with exposure: 54(32/22, 30.2±11.0);<br><br>HC: 19(21/15, 30.1 ± 10.4).	PANSS Total, 42.2 ±10.4	100% on antipsychotics (Olanzapine or Risperidone)	Actiwatch AW4, (Cambridge Neurotechnology Inc., UK)	30 seconds	7 days	SSD patients have increased **total sleep time** (mean 502.45min, SD 59.5min) compared to HC (381.6min, SD 42.3min);<br><br>SSD patients spend more **time in bed** (mean 590.30min, SD 66.30min) compared to HC (454.4min, SD 48.2min);<br><br>SSD participants have increased **sleep latency** (mean 29.45min, SD 17.75) compared to HC (mean 22.3min, SD 22.3min);<br><br>SSD participants have increased **sleep efficiency** (mean 85.25%, SD 5.35%) compared to HC (mean 84.1%, SD 6.1%).
Wulff et al., 2012	SSD	Individuals with exposure: 20(15/5, 38.8 ±8.6);<br><br>HC: 21(13/8, 37.5±9.6).	NR	60% on antipsychotics	Actiwatch-L (Cambridge Neurotechnology Ltd, Cambridge, UK)	120 seconds	42 days	SSD patients have increased **total sleep time** (493.20±451.2–535.2 (95% CI)) compared to HC (364.80 ±346.8-381.6 (95% CI));<br><br>SSD patients have increased **sleep latency** (34mins, 26-42 (CI)) compared to HC (19mins, 15-24 (CI));<br><br>No difference in **sleep efficiency** between the two groups.

Bartsh et al., 2021	CHR-P: genetic vulnerability	Individuals with exposure: 25 (25/0, age 21-23);<br><br>HC: 22 (22/0, age 21-23).	NR	NR	MotionWatch 8, CamNtech, UK	NR	14 days	No difference in **total sleep time, time in bed, sleep latency and sleep efficiency** between the two groups.
Henning et al., 2020	CHR-P	Individuals with exposure: 41(15/26 21.15±1.54);<br><br>HC: 41(14/27, 21.34±1.56).	CAPE total, 44.98 ± 9.55<br><br>CAPE positive, 3.17 ± 1.07<br><br>CAPE negative, 17.21 ± 6.2	NR	Actiwatch 2 (Philips Respironic)	30 seconds	14 days	No difference in **total sleep time**, **sleep efficiency** and **wake after sleep onset**
Lunsford-Avery et al., 2015	CHR-P: ultra-high risk	Individuals with exposure: 38 (19/19, 18.7 ±1.9);<br><br>HC: 31(16/15, 17.9 ±2.6).	SIPS positive exposure, 11.81 ± 4.67<br><br>SIPS negative exposure, 9.28 ± 6.72	6% on antipsychotics	ActiSleep monitors (ActiGraph; Pensacola, FL)	60 seconds	5 days	No difference in **total sleep time** between the two groups.<br><br>Ultra-high-risk participants have increased **wake after sleep onset** (72.05mins, SD 52.1mins) compared to HC (55.78mins, SD 23.07mins);<br><br>Ultra-high-risk participants have reduced **sleep efficiency** (mean 84.78%, SD 8.82%) compared to HC (87.99%±4.20%);<br><br>No difference in **nighttime awakenings time** between the two groups.
Nordholm et al., 2023	CHR-P: ultra-high risk	Individuals with exposure: 72 (30/42, 23.9± 3.9);<br><br>HC: 36 (19/17,23.8 ± 2.5).	CAARMS, 49.5 ± 15.7	33% on antipsychotics	ActiGraph wGT3X-BT	60 seconds	1 day	No difference in **total sleep time**, **time in bed**, **wake after sleep onset, sleep efficiency** and **nighttime awakenings** between the two groups.

Ristanovic et al., 2022	CHR-P	Individuals with exposure: 57(23/34,18.89±1.82);<br><br>HC: 61(33/28, 18.34 ±2.41).	NR	NR	ActiSleep monitors (ActiGraph; Pensacola, FL)	60 Seconds	5 days	No difference in **total sleep time** between the two groups.<br><br>CHR-P patients have decreased **sleep efficiency** (mean 84.7%, SD 8.79%) compared to HC (88.1%, SD 4.61);<br><br>CHR-P patients have increased **wake after sleep onset** (71.3mins, SD 51.35mins) compared to HC (54.21mins, SD 23.68mins);

ROBINS-E

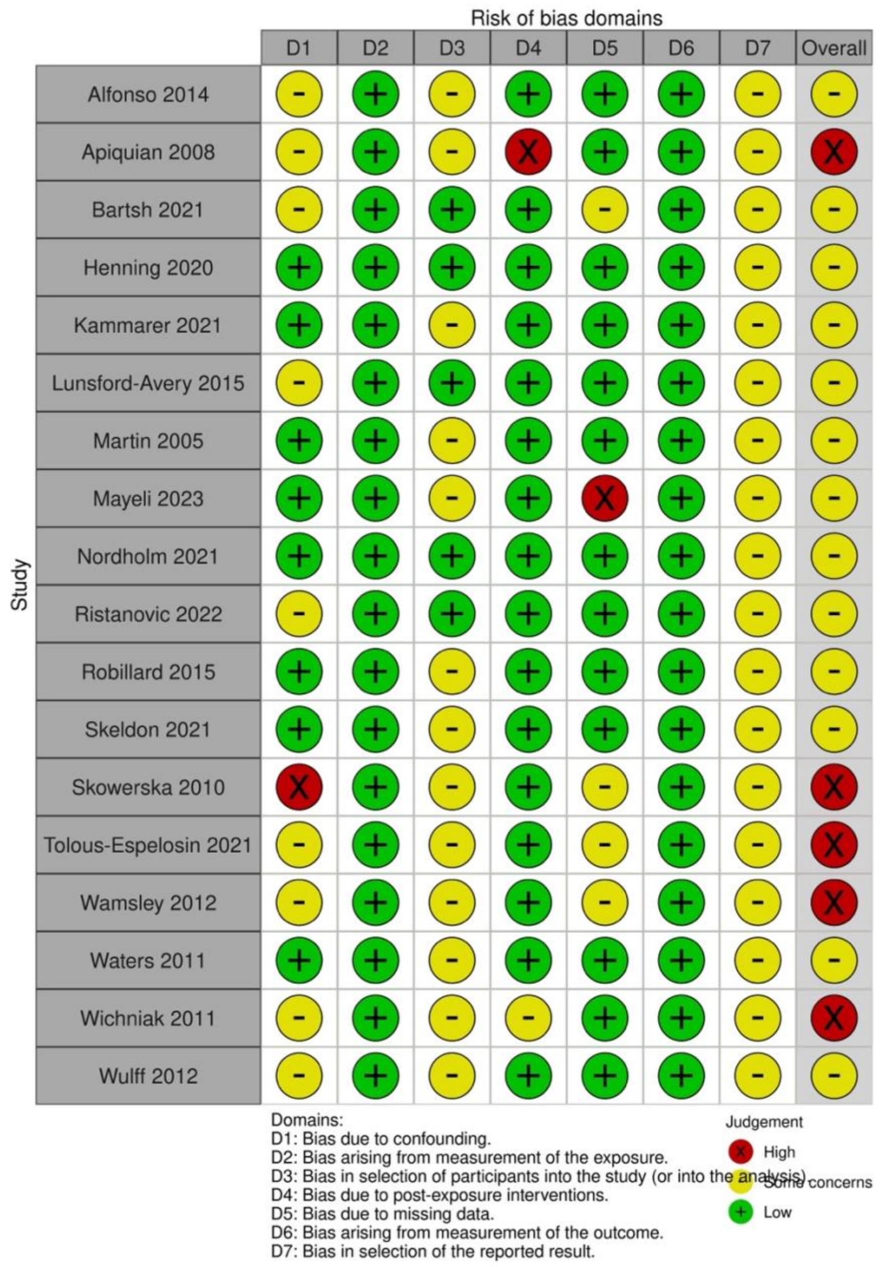

Figure 1

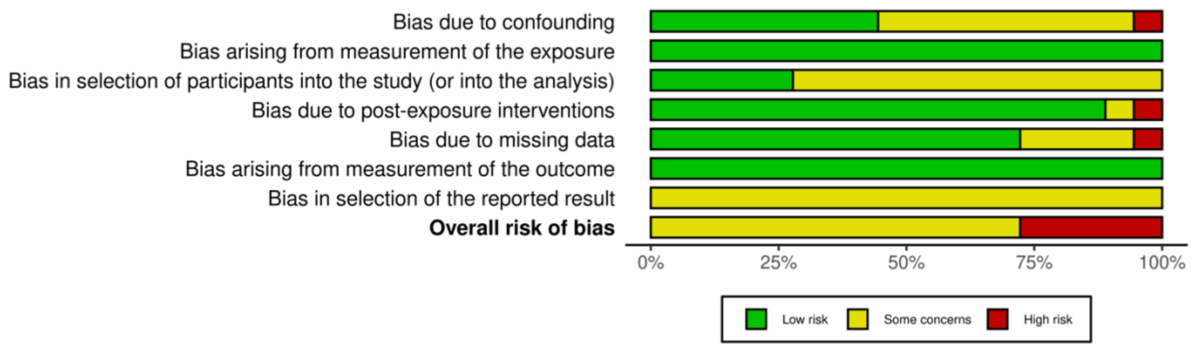

Figure 2

RESULTS TST

All studies

Forest plot

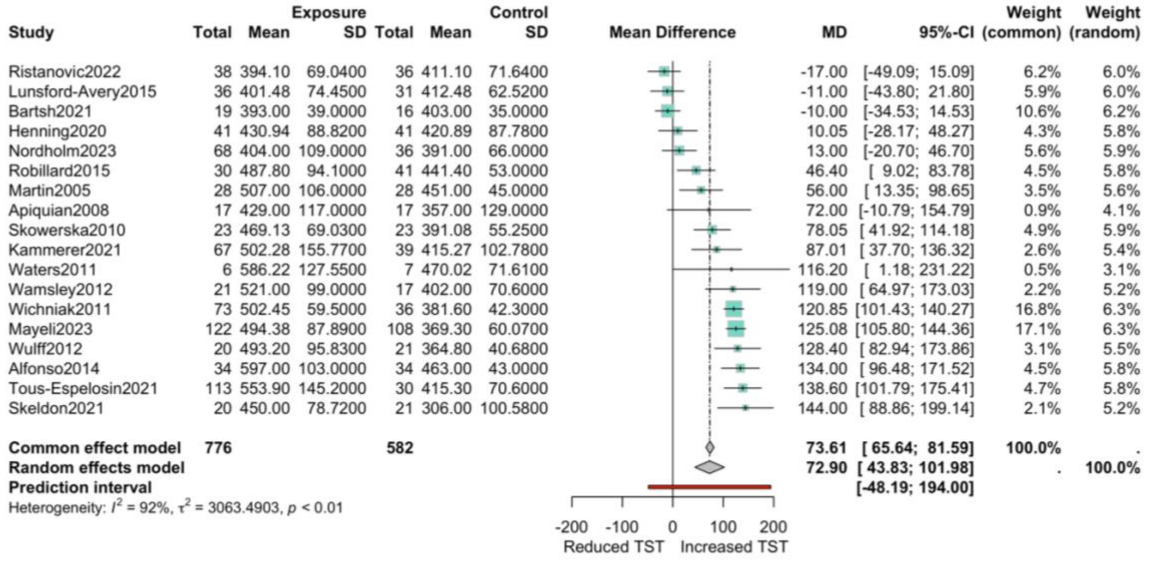

Figure 3

	MD	95%-CI	%W(common)	%W(random)
Alfonso2014	134.0000	[96.4826; 171.5174]	4.5	5.8
Apiquian2008	72.0000	[-10.7866; 154.7866]	0.9	4.1
Bartsh2021	-10.0000	[-34.5282; 14.5282]	10.6	6.2
Henning2020	10.0500	[-28.1743; 48.2743]	4.3	5.8
Kammerer2021	87.0100	[37.6976; 136.3224]	2.6	5.4
Lunsford-Avery2015	-11.0000	[-43.7997; 21.7997]	5.9	6.0
Mayeli2023	125.0800	[105.8037; 144.3563]	17.1	6.3
Martin2005	56.0000	[13.3463; 98.6537]	3.5	5.6
Nordholm2023	13.0000	[-20.7046; 46.7046]	5.6	5.9
Ristanovic2022	-17.0000	[-49.0859; 15.0859]	6.2	6.0

Robillard2015	46.4000 [9.0231; 83.7769]	4.5	5.8
Skeldon2021	144.0000 [88.8566; 199.1434]	2.1	5.2
Skowerska2010	78.0500 [41.9154; 114.1846]	4.9	5.9
Tous-Espelosin2021	138.6000 [101.7902; 175.4098]	4.7	5.8
Wamsley2012	119.0000 [64.9707; 173.0293]	2.2	5.2
Waters2011	116.2000 [1.1772; 231.2228]	0.5	3.1
Wichniak2011	120.8500 [101.4276; 140.2724]	16.8	6.3
Wulff2012	128.4000 [82.9401; 173.8599]	3.1	5.5

Number of studies: k = 18
Number of observations: o = 1358 (o.e = 776, o.c = 582)

	MD	95%-CI	z|t	p-value
Common effect model	73.6149	[65.6436; 81.5862]	18.10	< 0.0001
Random effects model	72.9037	[43.8311; 101.9762]	5.29	< 0.0001
Prediction interval		[-48.1910; 193.9983]		

Quantifying heterogeneity:
tau^2 = 3063.4903 [1482.4852; 6976.0096]; tau = 55.3488 [38.5031; 83.5225]
I^2 = 92.1% [88.9%; 94.3%]; H = 3.55 [3.01; 4.19]

Test of heterogeneity:

Q	d.f.	p-value
214.03	17	< 0.0001

Details on meta-analytical method:
- Inverse variance method
- Restricted maximum-likelihood estimator for tau^2
- Q-Profile method for confidence interval of tau^2 and tau
- Hartung-Knapp adjustment for random effects model (df = 17)
- Prediction interval based on t-distribution (df = 16)

Funnel plot

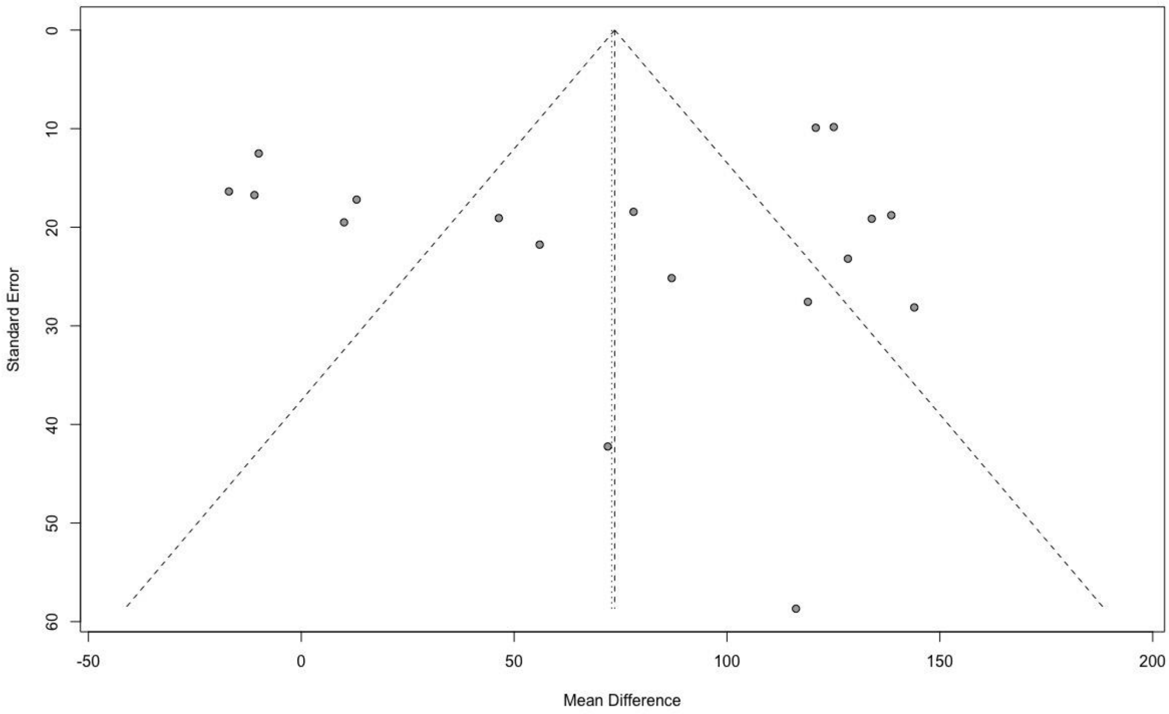

Figure 4

Linear regression test of funnel plot asymmetry

Test result: t = -0.29, df = 16, p-value = 0.7733
Bias estimate: -0.6685 (SE = 2.2816)

Details:
- multiplicative residual heterogeneity variance (tau^2 = 13.3054)
- predictor: standard error
- weight: inverse variance
- reference: Egger et al. (1997), BMJ

Eggers' test of the intercept

intercept	95% CI	t	p
-0.669	-5.14 - 3.8	-0.293	0.7732816

Eggers' test does not indicate the presence of funnel plot asymmetry.

Outliers

Identified outliers (fixed-effect model)

"Alfonso2014", "Bartsh2021", "Henning2020", "Lunsford-Avery2015", "Mayeli2023", "Nordholm2023", "Ristanovic2022", "Skeldon2021", "Tous-Espelosin2021", "Wichniak2011", "Wulff2012"

Results with outliers removed

Review: Total Sleep time (TST)

Number of studies: k = 7
Number of observations: o = 364 (o.e = 192, o.c = 172)

```
                      MD            95%-CI  z|t  p-value
Common effect model  73.0405 [54.8032;  91.2779] 7.85 < 0.0001
Random effects model 73.9256 [50.2659;  97.5854] 7.65   0.0003
Prediction interval          [37.0142; 110.8371]

Quantifying heterogeneity:
 tau^2 = 100.9057 [0.0000; >1009.0565]; tau = 10.0452 [0.0000; >31.7656]
 I^2 = 4.3% [0.0%; 72.1%]; H = 1.02 [1.00; 1.89]

Test of heterogeneity:
    Q d.f. p-value
 6.27    6  0.3939
```

Details on meta-analytical method:
- Inverse variance method
- Restricted maximum-likelihood estimator for tau^2
- Q-Profile method for confidence interval of tau^2 and tau
- Hartung-Knapp adjustment for random effects model (df = 6)
- Prediction interval based on t-distribution (df = 5)

Identified outliers (random-effects model)

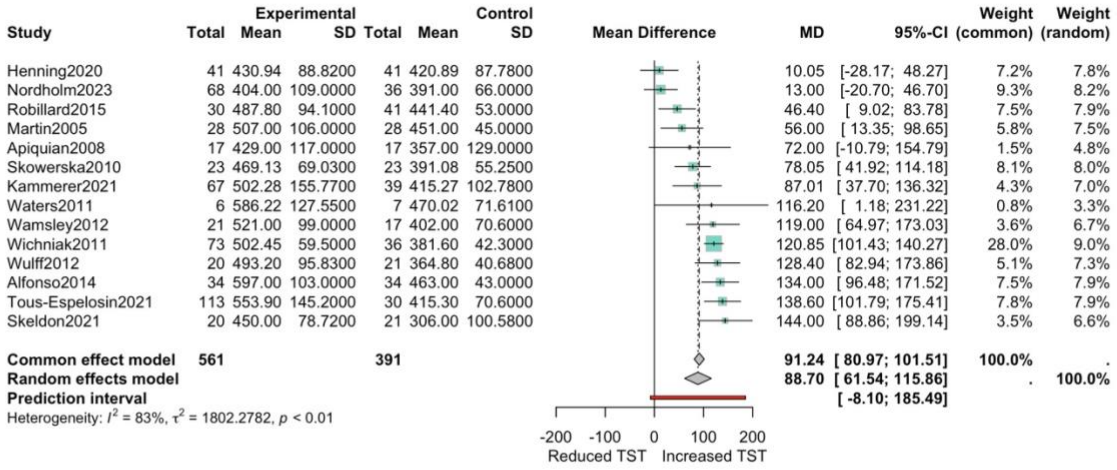

Figure 5

"Bartsh2021", "Lunsford-Avery2015", "Mayeli2023", "Ristanovic2022"

Results with outliers removed

Review: Total Sleep time (TST)

Number of studies: k = 14
Number of observations: o = 952 (o.e = 561, o.c = 391)

```
                     MD            95%-CI  z|t  p-value
Common effect model  91.2398 [80.9710; 101.5085] 17.41 < 0.0001
Random effects model 88.6958 [61.5357; 115.8559]  7.06 < 0.0001
Prediction interval          [-8.0982; 185.4899]
```

Quantifying heterogeneity:
 tau^2 = 1802.2782 [668.2415; 4918.8877]; tau = 42.4532 [25.8504; 70.1348]
 I^2 = 82.5% [71.9%; 89.1%]; H = 2.39 [1.89; 3.04]

Test of heterogeneity:
```
    Q d.f.  p-value
 74.49   13 < 0.0001
```

Details on meta-analytical method:
- Inverse variance method
- Restricted maximum-likelihood estimator for tau^2
- Q-Profile method for confidence interval of tau^2 and tau
- Hartung-Knapp adjustment for random effects model (df = 13)
- Prediction interval based on t-distribution (df = 12)

Influence analyses

Baujat plot

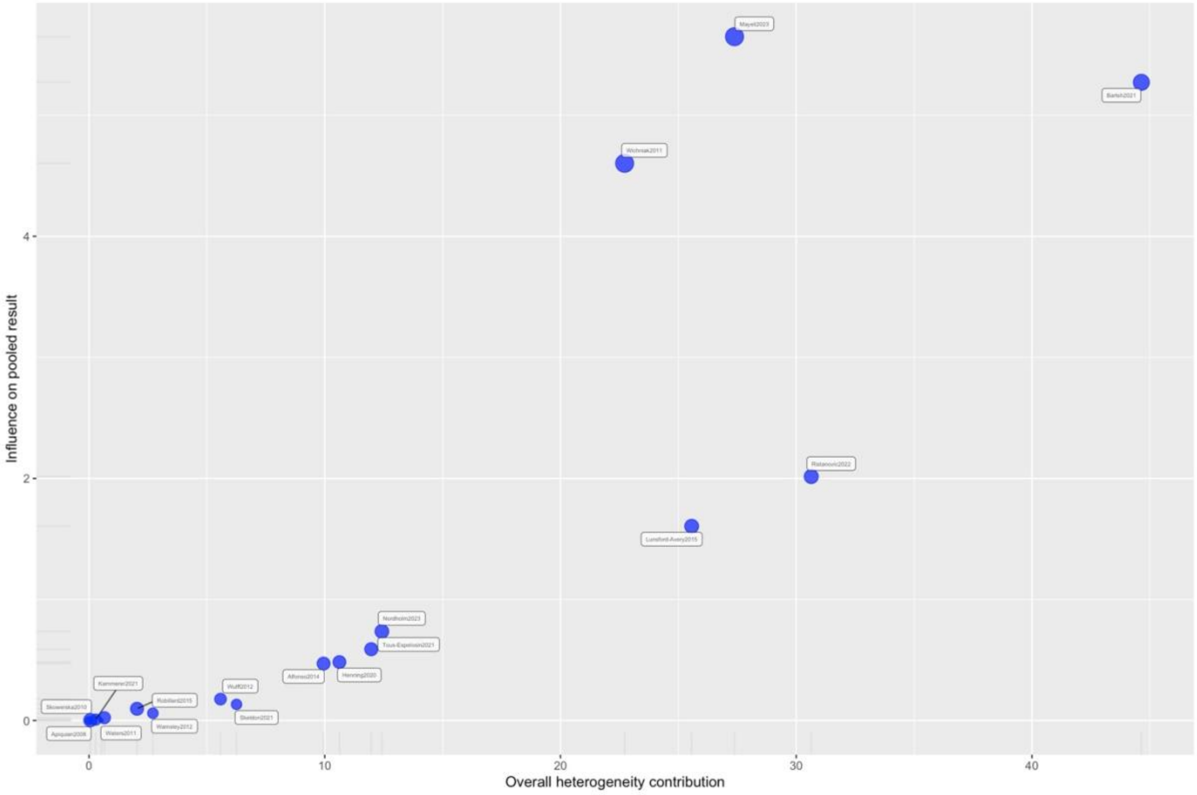

Figure 6

Influence diagnostics

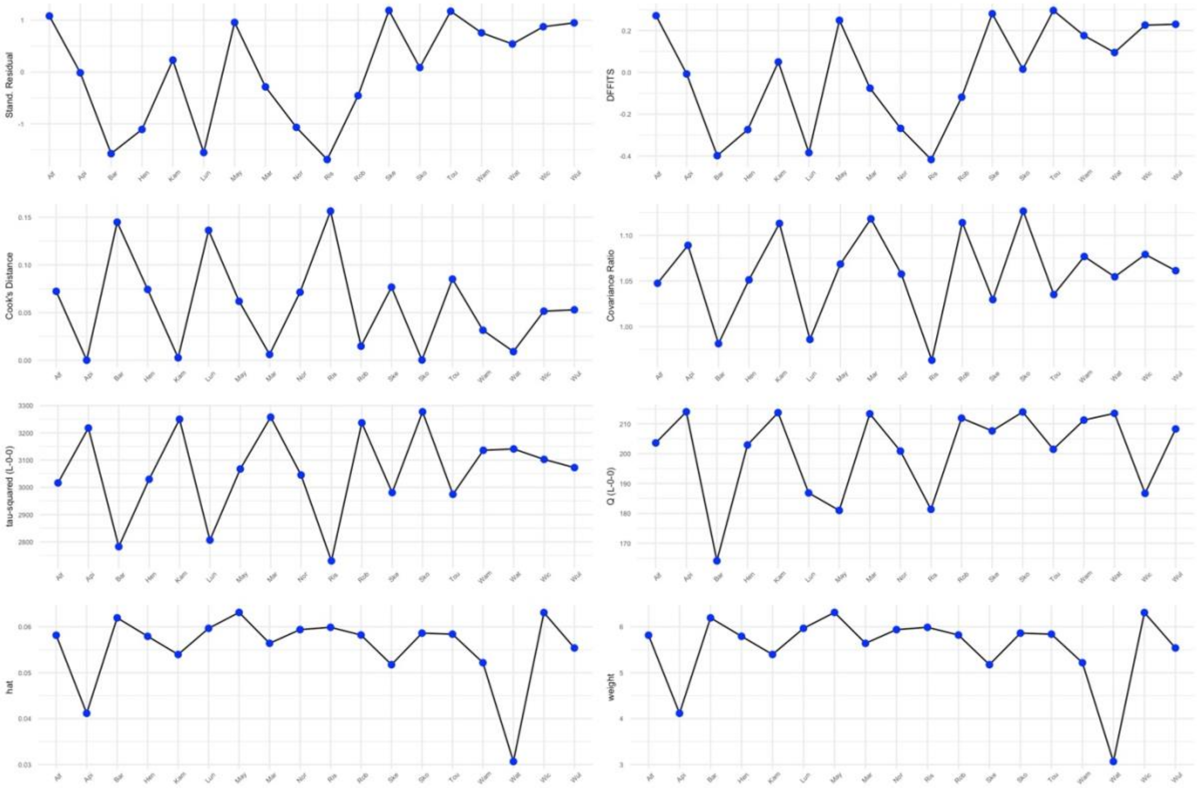

Figure 7

Meta-regression

Year

Mixed-Effects Model (k = 18; tau^2 estimator: REML)

tau^2 (estimated amount of residual heterogeneity): 3101.2172 (SE = 1273.8088)
tau (square root of estimated tau^2 value): 55.6886
I^2 (residual heterogeneity / unaccounted variability): 90.46%
H^2 (unaccounted variability / sampling variability): 10.48
R^2 (amount of heterogeneity accounted for): 0.00%

Test for Residual Heterogeneity:
QE(df = 16) = 203.6156, p-val < .0001

Test of Moderators (coefficient 2):
F(df1 = 1, df2 = 16) = 0.7894, p-val = 0.3874

Model Results:

	estimate	se	tval	df	pval	ci.lb	ci.ub
intrcpt	4554.6189	5044.1183	0.9030	16	0.3799	-6138.4343	15247.6721
year	-2.2229	2.5019	-0.8885	16	0.3874	-7.5266	3.0808

Signif. codes: 0 ‘***’ 0.001 ‘**’ 0.01 ‘*’ 0.05 ‘.’ 0.1 ‘ ’ 1

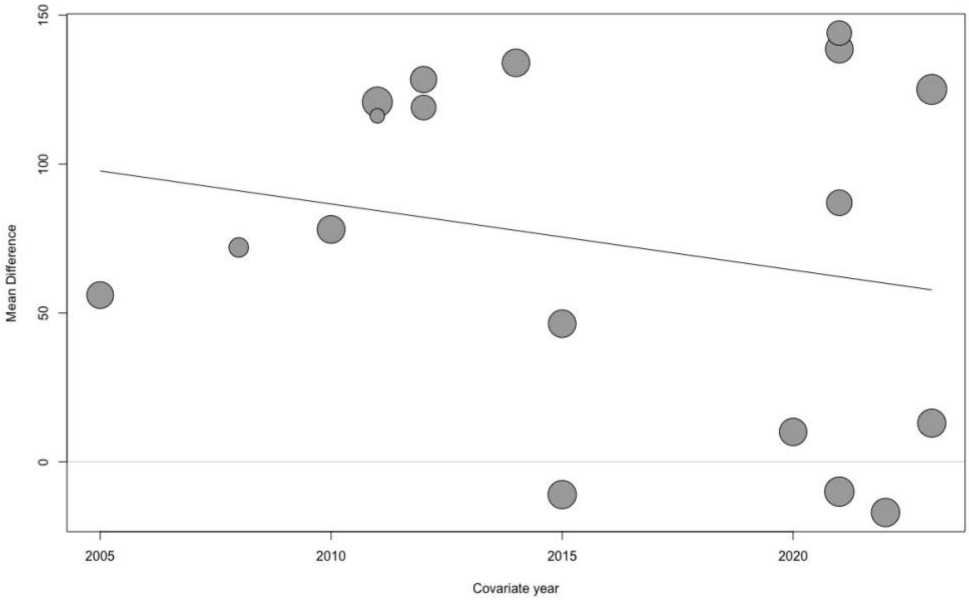

Figure 8

Percentage of treatment with antipsychotics

Mixed-Effects Model (k = 13; tau^2 estimator: REML)

tau^2 (estimated amount of residual heterogeneity): 273.9015 (SE = 267.1576)
tau (square root of estimated tau^2 value): 16.5500
I^2 (residual heterogeneity / unaccounted variability): 46.29%
H^2 (unaccounted variability / sampling variability): 1.86
R^2 (amount of heterogeneity accounted for): 88.14%

Test for Residual Heterogeneity:
QE(df = 11) = 18.9248, p-val = 0.0625

Test of Moderators (coefficient 2):
F(df1 = 1, df2 = 11) = 37.2345, p-val < .0001

Model Results:

	estimate	se	tval	df	pval	ci.lb	ci.ub	
intrcpt	-20.3243	19.8486	-1.0240	11	0.3278	-64.0106	23.3621	
Percentage	141.6507	23.2138	6.1020	11	<.0001	90.5575	192.7438	***

Signif. codes: 0 ‘***’ 0.001 ‘**’ 0.01 ‘*’ 0.05 ‘.’ 0.1 ‘ ’ 1

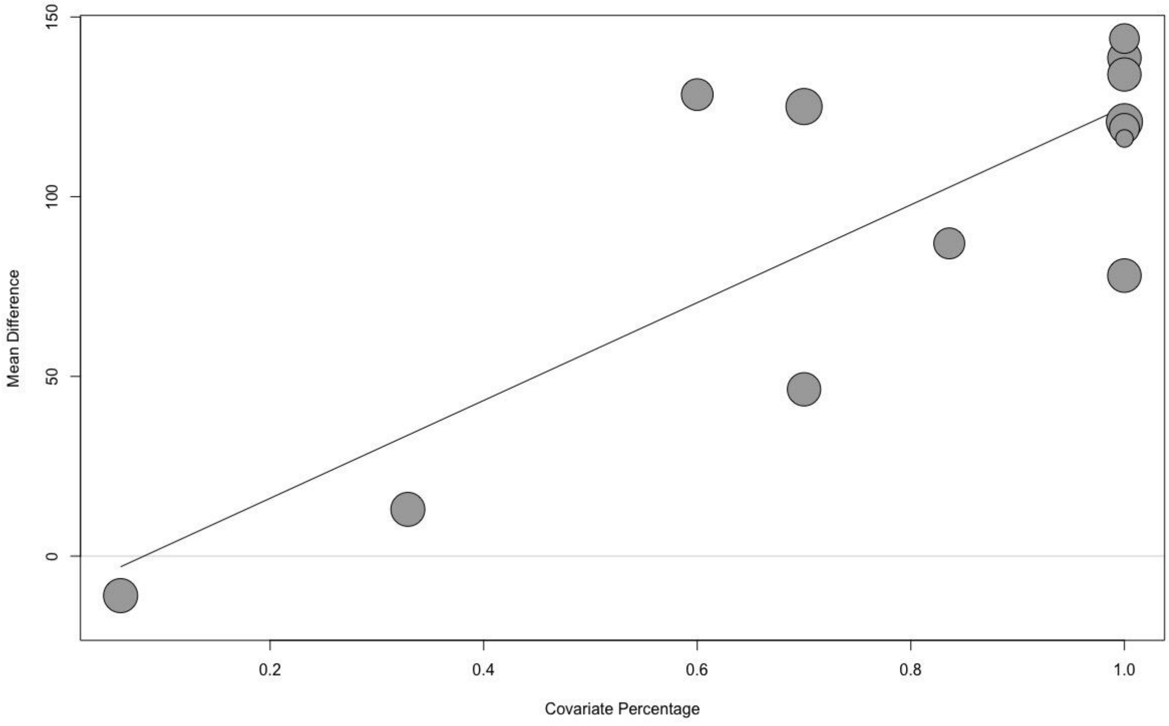

Figure 9

Age

Mixed-Effects Model (k = 17; tau^2 estimator: REML)

tau^2 (estimated amount of residual heterogeneity): 1714.0963 (SE = 803.2642)
tau (square root of estimated tau^2 value): 41.4016
I^2 (residual heterogeneity / unaccounted variability): 83.55%
H^2 (unaccounted variability / sampling variability): 6.08
R^2 (amount of heterogeneity accounted for): 38.39%

Test for Residual Heterogeneity:
QE(df = 15) = 94.6219, p-val < .0001

Test of Moderators (coefficient 2):
F(df1 = 1, df2 = 15) = 9.4568, p-val = 0.0077

Model Results:

	estimate	se	tval	df	pval	ci.lb	ci.ub	
intrcpt	-30.4437	36.8891	-0.8253	15	0.4221	-109.0709	48.1835	
age_mean_e	3.2613	1.0605	3.0752	15	0.0077	**1.0009**	**5.5217**	**

Signif. codes: 0 ‘***’ 0.001 ‘**’ 0.01 ‘*’ 0.05 ‘.’ 0.1 ‘ ’ 1

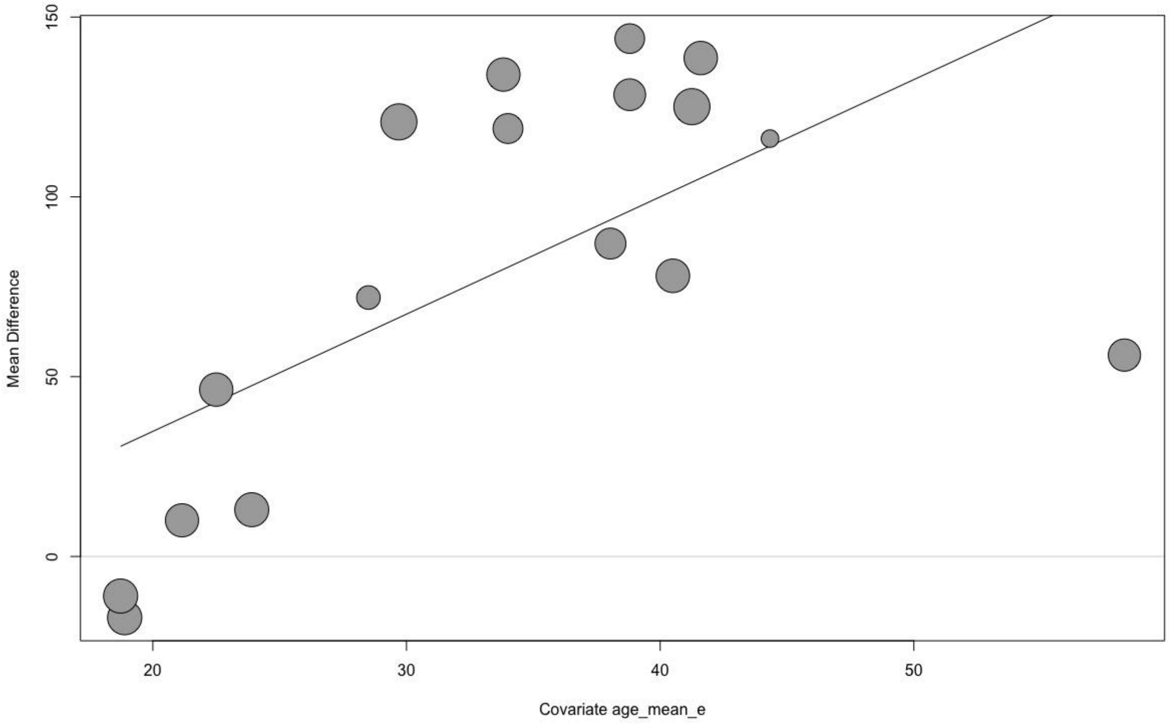

Figure 10

Gender_female

Mixed-Effects Model (k = 17; tau^2 estimator: REML)

tau^2 (estimated amount of residual heterogeneity): 2050.6898 (SE = 928.3807)
tau (square root of estimated tau^2 value): 45.2845
I^2 (residual heterogeneity / unaccounted variability): 86.22%
H^2 (unaccounted variability / sampling variability): 7.26
R^2 (amount of heterogeneity accounted for): 26.29%

Test for Residual Heterogeneity:
QE(df = 15) = 116.8958, p-val < .0001

Test of Moderators (coefficient 2):
F(df1 = 1, df2 = 15) = 6.0850, p-val = 0.0262

Model Results:

	estimate	se	tval	df	pval	ci.lb	ci.ub	
intrcpt	166.7873	37.9309	4.3971	15	0.0005	85.9394	247.6352	***
female_percentage_e	-217.4858	88.1660	-2.4668	15	0.0262	**-405.4073**	**-29.5644**	*

Signif. codes: 0 '***' 0.001 '**' 0.01 '*' 0.05 '.' 0.1 ' ' 1

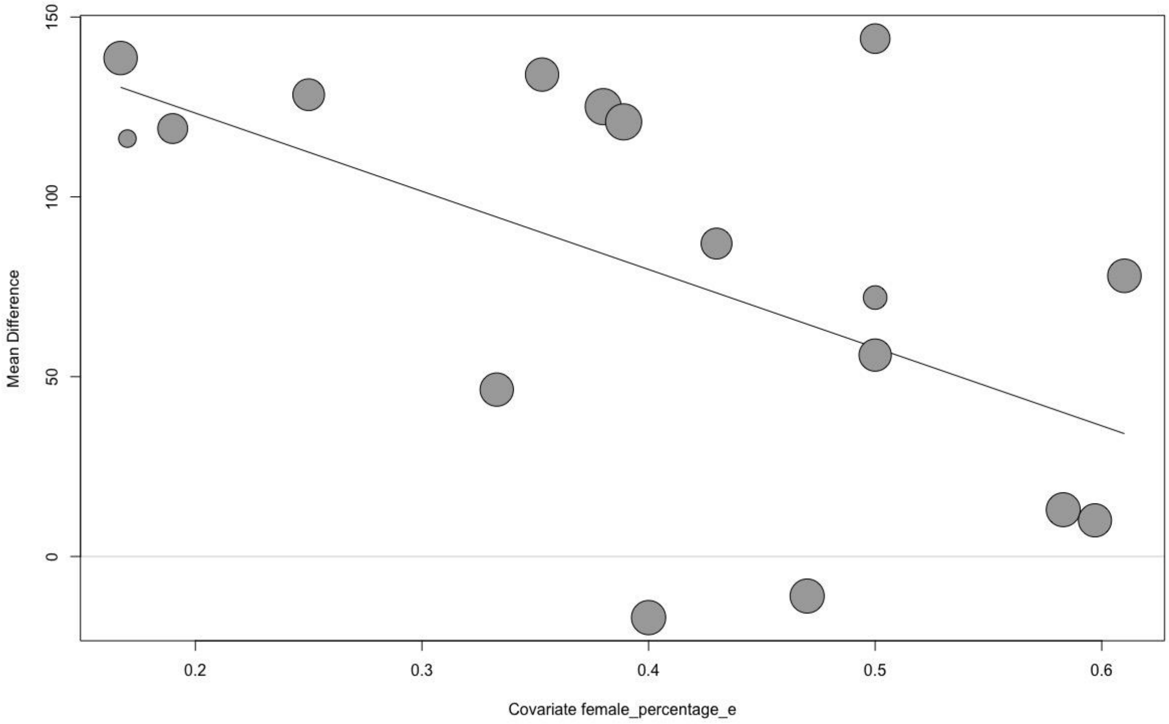

Figure 11

PANSS Total score

```
Mixed-Effects Model (k = 5; tau^2 estimator: REML)

tau^2 (estimated amount of residual heterogeneity):     0 (SE = 217.8419)
tau (square root of estimated tau^2 value):             0
I^2 (residual heterogeneity / unaccounted variability): 0.00%
H^2 (unaccounted variability / sampling variability):   1.00
R^2 (amount of heterogeneity accounted for):            100.00%

Test for Residual Heterogeneity:
QE(df = 3) = 2.4951, p-val = 0.4762

Test of Moderators (coefficient 2):
F(df1 = 1, df2 = 3) = 4.8623, p-val = 0.1146

Model Results:

            estimate      se    tval  df    pval    ci.lb     ci.ub
intrcpt      65.1629  22.7228  2.8677   3  0.0642  -7.1510  137.4769  .
PANSS_mean    1.1668   0.5292  2.2051   3  0.1146  -0.5172    2.8509

---
Signif. codes:  0 '***' 0.001 '**' 0.01 '*' 0.05 '.' 0.1 ' ' 1
```

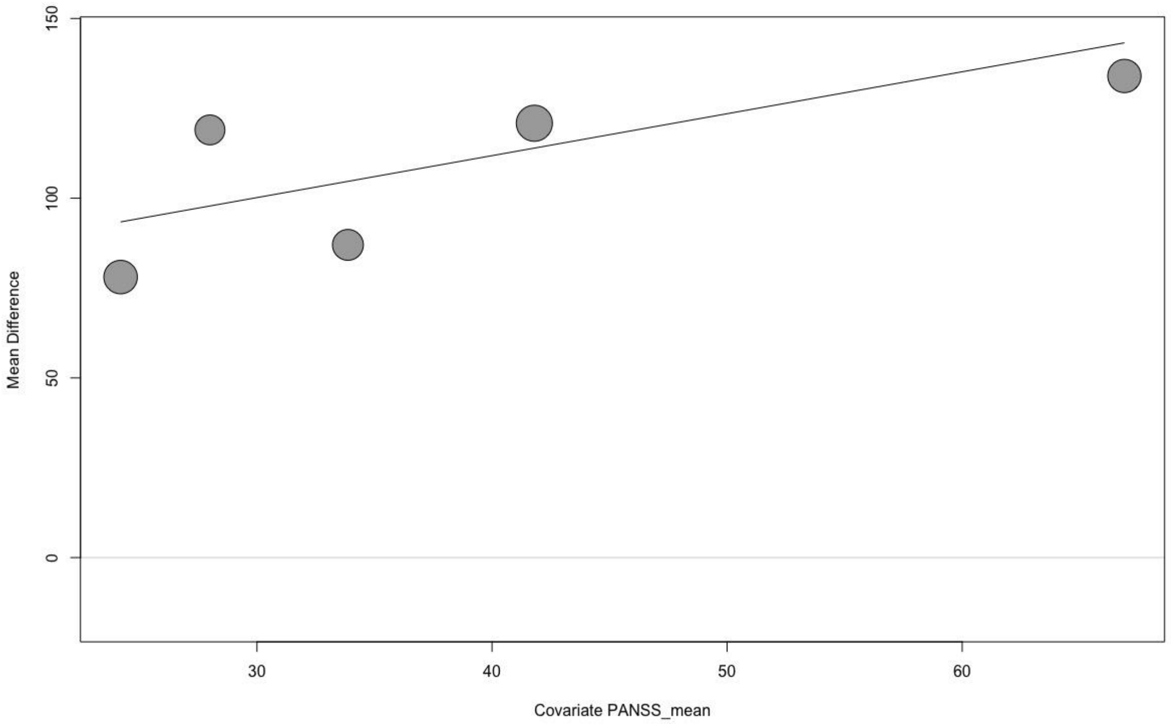

Figure 12

Study sample

Mixed-Effects Model (k = 18; tau^2 estimator: REML)

tau^2 (estimated amount of residual heterogeneity): 2951.6572 (SE = 1222.7229)
tau (square root of estimated tau^2 value): 54.3292
I^2 (residual heterogeneity / unaccounted variability): 89.82%
H^2 (unaccounted variability / sampling variability): 9.83
R^2 (amount of heterogeneity accounted for): 3.65%

Test for Residual Heterogeneity:
QE(df = 16) = 146.9108, p-val < .0001

Test of Moderators (coefficient 2):
F(df1 = 1, df2 = 16) = 1.1975, p-val = 0.2900

Model Results:

	estimate	se	tval	df	pval	ci.lb	ci.ub	
intrcpt	51.7839	23.6324	2.1912	16	0.0436	1.6854	101.8824	*
n_e	0.4644	0.4243	1.0943	16	0.2900	-0.4352	1.3639	

Signif. codes: 0 '***' 0.001 '**' 0.01 '*' 0.05 '.' 0.1 ' ' 1

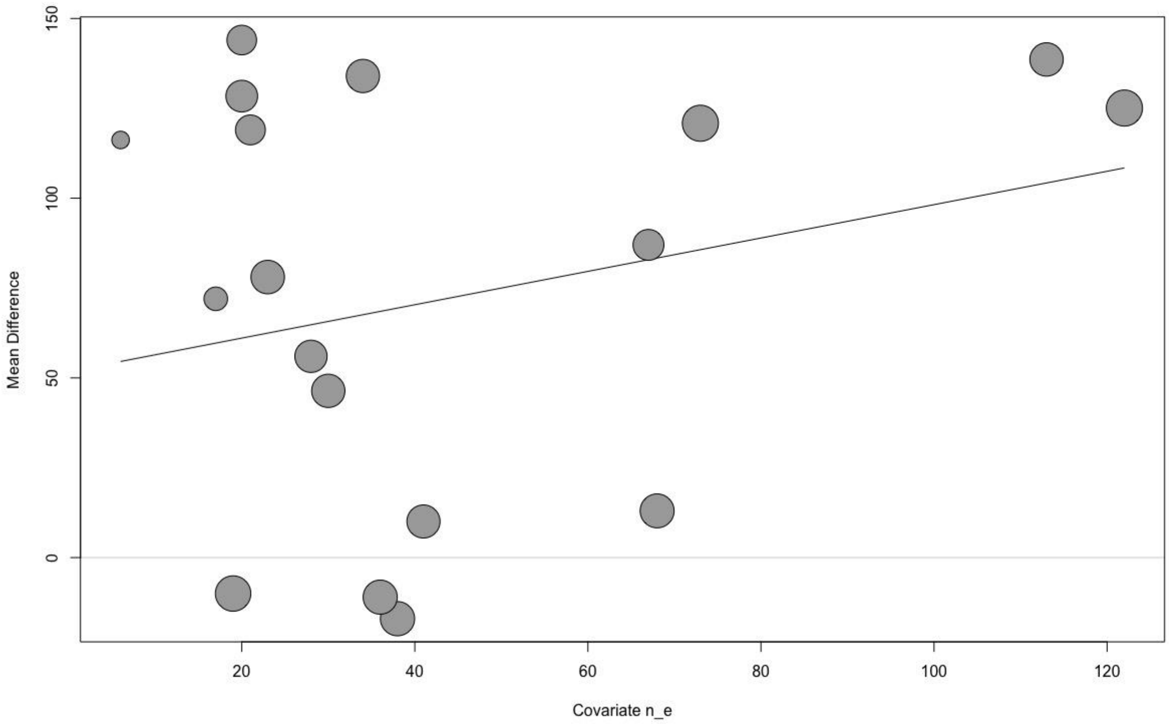

Figure 13

Geography

Mixed-Effects Model (k = 18; tau^2 estimator: REML)

tau^2 (estimated amount of residual heterogeneity): 2934.4425 (SE = 1247.9344)
tau (square root of estimated tau^2 value): 54.1705
I^2 (residual heterogeneity / unaccounted variability): 90.46%
H^2 (unaccounted variability / sampling variability): 10.48
R^2 (amount of heterogeneity accounted for): 4.21%

Test for Residual Heterogeneity:
QE(df = 15) = 168.7911, p-val < .0001

Test of Moderators (coefficients 2:3):
F(df1 = 2, df2 = 15) = 1.1614, p-val = 0.3397

Model Results:

	estimate	se	tval	df	pval	ci.lb	ci.ub
intrcpt	70.1901	45.9358	1.5280	15	0.1473	-27.7197	168.1000
ContinentEurope	17.0032	48.9937	0.3470	15	0.7334	-87.4243	121.4308
ContinentNorth_America	-30.7647	52.9499	-0.5810	15	0.5699	-143.6248	82.0953

Signif. codes: 0 ‘***’ 0.001 ‘**’ 0.01 ‘*’ 0.05 ‘.’ 0.1 ‘ ’ 1

Epochs

Mixed-Effects Model (k = 15; tau^2 estimator: REML)

tau^2 (estimated amount of residual heterogeneity): 3301.7466 (SE = 1521.3485)
tau (square root of estimated tau^2 value): 57.4608
I^2 (residual heterogeneity / unaccounted variability): 89.45%
H^2 (unaccounted variability / sampling variability): 9.48
R^2 (amount of heterogeneity accounted for): 0.00%

Test for Residual Heterogeneity:
QE(df = 13) = 137.8708, p-val < .0001

Test of Moderators (coefficient 2):
F(df1 = 1, df2 = 13) = 0.0399, p-val = 0.8447

Model Results:

	estimate	se	tval	df	pval	ci.lb	ci.ub	
intrcpt	69.7987	29.9964	2.3269	13	0.0368	4.9955	134.6019	*
Epochs_seconds	0.0957	0.4787	0.1999	13	0.8447	-0.9385	1.1298	

Signif. codes: 0 ‘***’ 0.001 ‘**’ 0.01 ‘*’ 0.05 ‘.’ 0.1 ‘ ’ 1

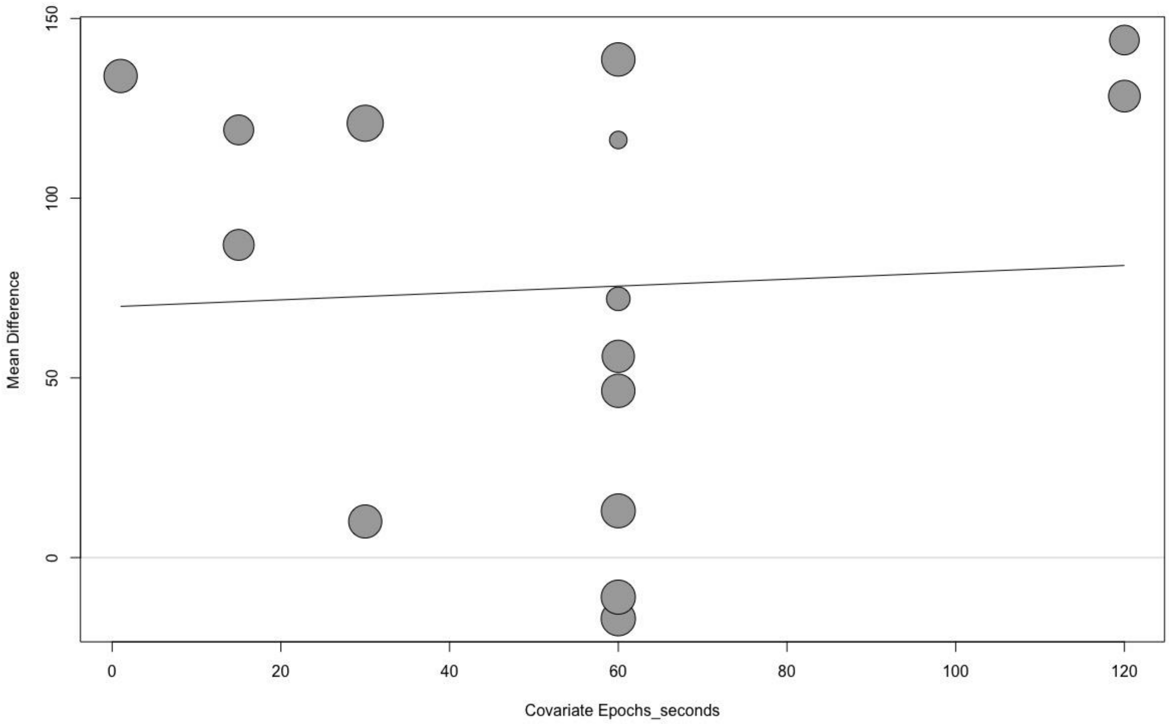

Figure 14

Follow-up_mean

Mixed-Effects Model (k = 17; tau^2 estimator: REML)

tau^2 (estimated amount of residual heterogeneity): 3141.2595 (SE = 1330.3453)
tau (square root of estimated tau^2 value): 56.0469
I^2 (residual heterogeneity / unaccounted variability): 91.18%
H^2 (unaccounted variability / sampling variability): 11.34
R^2 (amount of heterogeneity accounted for): 4.15%

Test for Residual Heterogeneity:
QE(df = 15) = 211.6633, p-val < .0001

Test of Moderators (coefficient 2):
F(df1 = 1, df2 = 15) = 1.9130, p-val = 0.1869

Model Results:

	estimate	se	tval	df	pval	ci.lb	ci.ub	
intrcpt	54.3284	19.4271	2.7965	15	0.0136	12.9206	95.7363	*
Follow_up_actigraphy_days_mean	1.6194	1.1709	1.3831	15	0.1869	-0.8762	4.1151	

Signif. codes: 0 '***' 0.001 '**' 0.01 '*' 0.05 '.' 0.1 ' ' 1

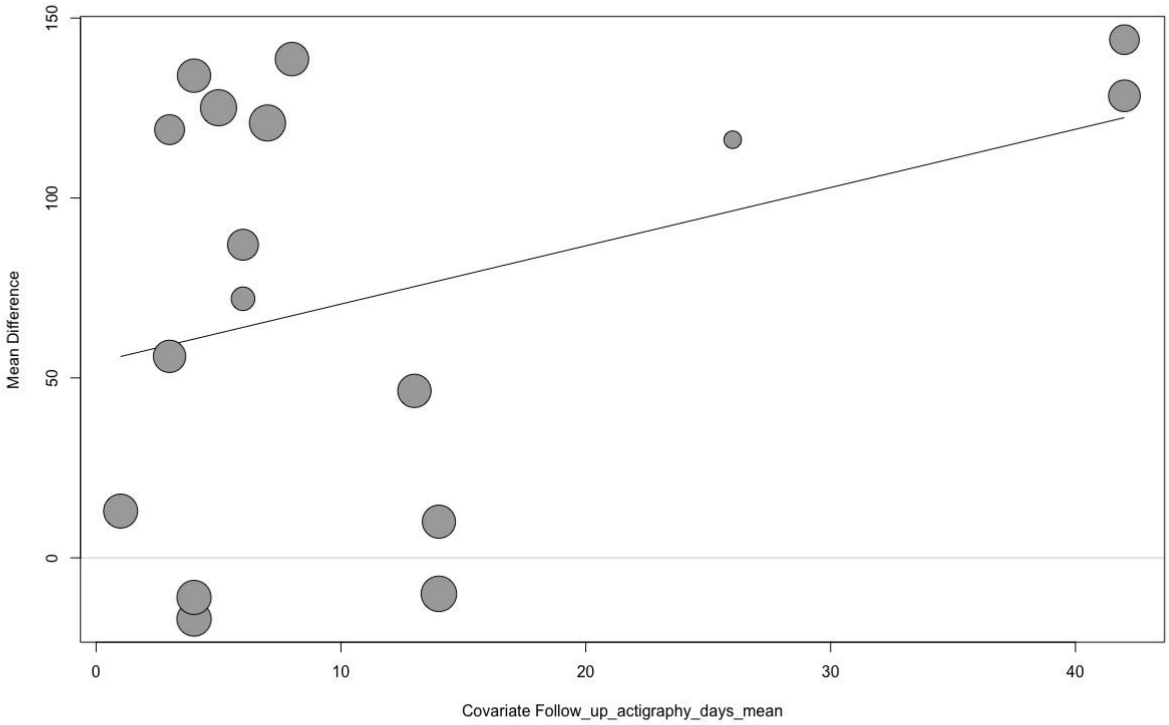

Figure 15

Follow-up_max

Mixed-Effects Model (k = 17; tau^2 estimator: REML)

tau^2 (estimated amount of residual heterogeneity):   3139.3833 (SE = 1329.8440)
tau (square root of estimated tau^2 value):   56.0302
I^2 (residual heterogeneity / unaccounted variability): 91.18%
H^2 (unaccounted variability / sampling variability):   11.33
R^2 (amount of heterogeneity accounted for):   4.21%

Test for Residual Heterogeneity:
QE(df = 15) = 211.1214, p-val < .0001

Test of Moderators (coefficient 2):
F(df1 = 1, df2 = 15) = 1.9181, p-val = 0.1863

Model Results:

	estimate	se	tval	df	pval	ci.lb	ci.ub	
intrcpt	52.4401	20.3653	2.5750	15	0.0211	9.0326	95.8476	*
Follow_up_actigraphy_days_max	1.6177	1.1681	1.3850	15	0.1863	-0.8720	4.1074	

Signif. codes: 0 ‘***’ 0.001 ‘**’ 0.01 ‘*’ 0.05 ‘.’ 0.1 ‘ ’ 1

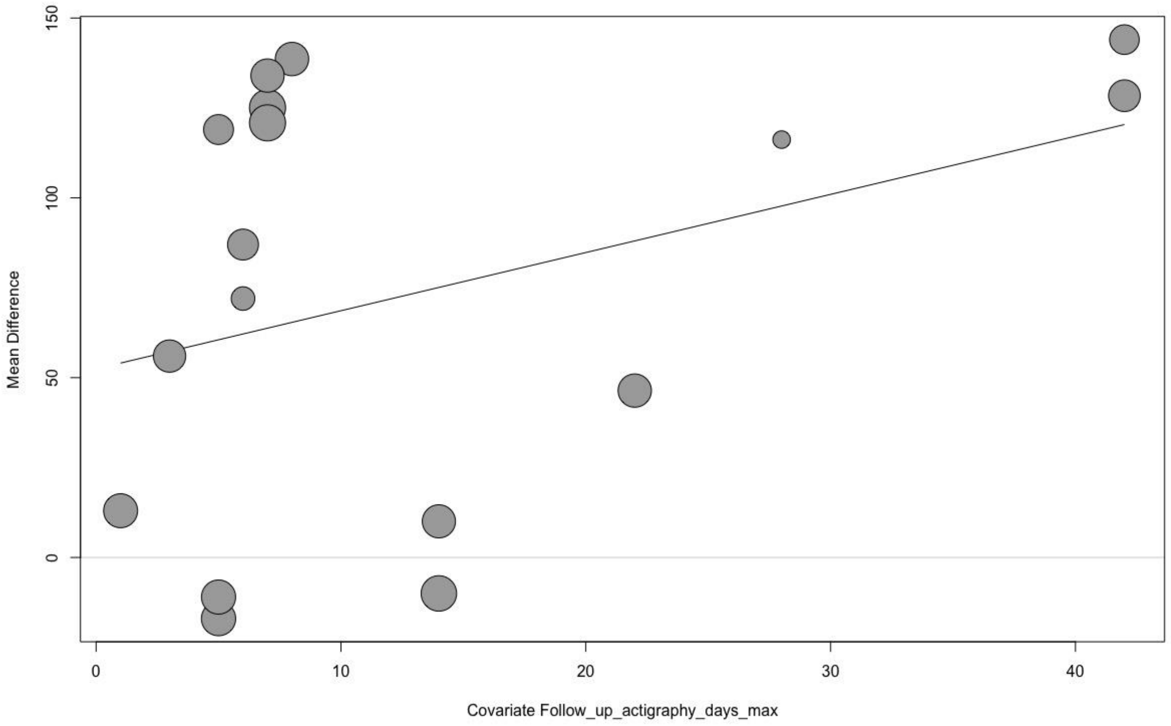

Figure 16

Subgroup analyses

Epochs < 60 seconds

Forest Plot

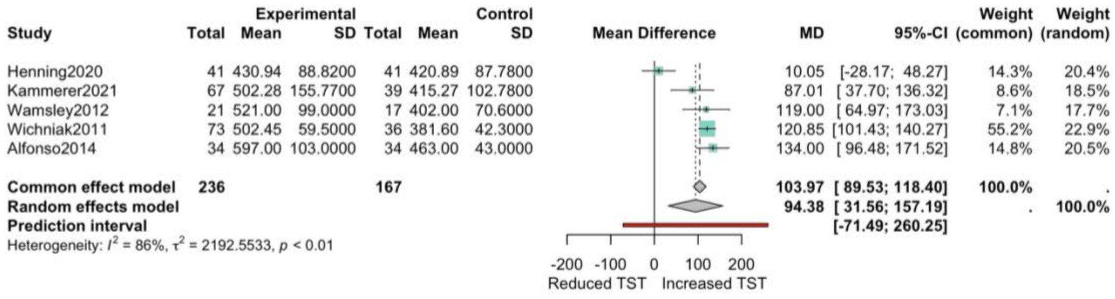

Figure 17

Number of studies: k = 5
Number of observations: o = 403 (o.e = 236, o.c = 167)

	MD	95%-CI	z|t	p-value
Common effect model	103.9650	[89.5306; 118.3994]	14.12	< 0.0001

Random effects model 94.3759 [31.5600; 157.1919] 4.17   0.0140
Prediction interval   [-71.4933; 260.2451]

Quantifying heterogeneity:
tau^2 = 2192.5533 [533.4927; 20348.4291]; tau = 46.8247 [23.0975; 142.6479]
I^2 = 86.4% [70.3%; 93.7%]; H = 2.71 [1.83; 4.00]

Test of heterogeneity:
Q d.f. p-value
29.31   4 < 0.0001

Details on meta-analytical method:
- Inverse variance method
- Restricted maximum-likelihood estimator for tau^2
- Q-Profile method for confidence interval of tau^2 and tau
- Hartung-Knapp adjustment for random effects model (df = 4)
- Prediction interval based on t-distribution (df = 3)

Epochs > = 60 seconds

Forest Plot

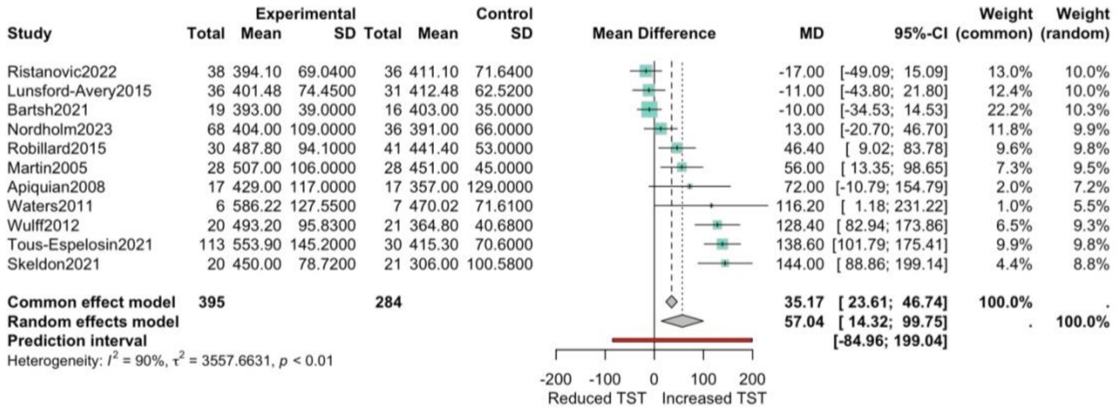

Figure 18

Number of studies: k = 11
Number of observations: o = 679 (o.e = 395, o.c = 284)

MD   95%-CI z|t p-value
Common effect model 35.1749 [23.6133; 46.7366] 5.96 < 0.0001
Random effects model 57.0372 [14.3196; 99.7547] 2.98   0.0139
Prediction interval   [-84.9620; 199.0364]

Quantifying heterogeneity:
tau^2 = 3557.6631 [1422.0318; 11557.2306]; tau = 59.6461 [37.7098; 107.5046]
I^2 = 89.8% [83.7%; 93.6%]; H = 3.13 [2.48; 3.95]

Test of heterogeneity:
    Q d.f. p-value
 97.84   10 < 0.0001

Details on meta-analytical method:
- Inverse variance method
- Restricted maximum-likelihood estimator for tau^2
- Q-Profile method for confidence interval of tau^2 and tau
- Hartung-Knapp adjustment for random effects model (df = 10)
- Prediction interval based on t-distribution (df = 9)

Follow-up >= 7 days

Forest Plot

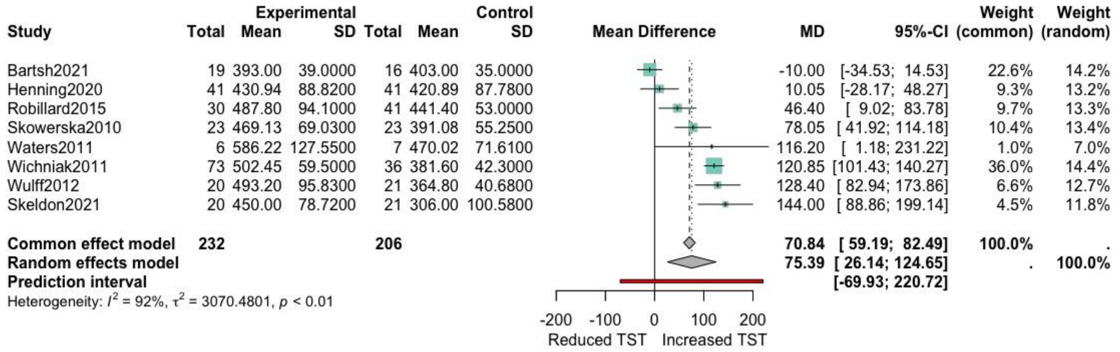

Figure 19

Number of studies: k = 8
Number of observations: o = 438 (o.e = 232, o.c = 206)

	MD	95%-CI	z|t	p-value
Common effect model	70.8355	[59.1851; 82.4859]	11.92	< 0.0001
Random effects model	75.3946	[26.1405; 124.6488]	3.62	0.0085
Prediction interval		[-69.9293; 220.7186]		

Quantifying heterogeneity:
 tau^2 = 3070.4801 [1055.5061; 13406.7611]; tau = 55.4119 [32.4886; 115.7876]
 I^2 = 92.4% [87.4%; 95.4%]; H = 3.63 [2.82; 4.68]

Test of heterogeneity:
    Q d.f. p-value
 92.22   7 < 0.0001

Details on meta-analytical method:
- Inverse variance method
- Restricted maximum-likelihood estimator for tau^2

- Q-Profile method for confidence interval of tau^2 and tau
- Hartung-Knapp adjustment for random effects model (df = 7)
- Prediction interval based on t-distribution (df = 6)

Follow-up < 7 days

Forest Plot

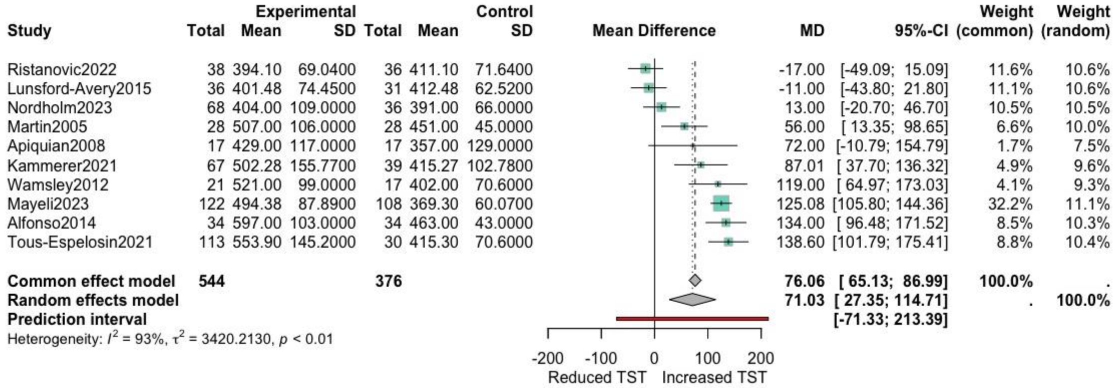

Figure 20

Number of studies: k = 10
Number of observations: o = 920 (o.e = 544, o.c = 376)

```
                     MD             95%-CI   z|t  p-value
Common effect model  76.0613 [ 65.1310;  86.9916] 13.64 < 0.0001
Random effects model 71.0295 [ 27.3519; 114.7071]  3.68   0.0051
Prediction interval          [-71.3279; 213.3869]
```

Quantifying heterogeneity:
tau^2 = 3420.2130 [1375.4626; 11559.5847]; tau = 58.4826 [37.0872; 107.5155]
I^2 = 92.6% [88.4%; 95.2%]; H = 3.67 [2.94; 4.59]

Test of heterogeneity:

```
     Q d.f.  p-value
 121.39    9 < 0.0001
```

Details on meta-analytical method:
- Inverse variance method
- Restricted maximum-likelihood estimator for tau^2
- Q-Profile method for confidence interval of tau^2 and tau
- Hartung-Knapp adjustment for random effects model (df = 9)
- Prediction interval based on t-distribution (df = 8)

Sensitive analyses

Three-arms study (Wichniak2011), only Olanzapine arm

Forest Plot, all studies

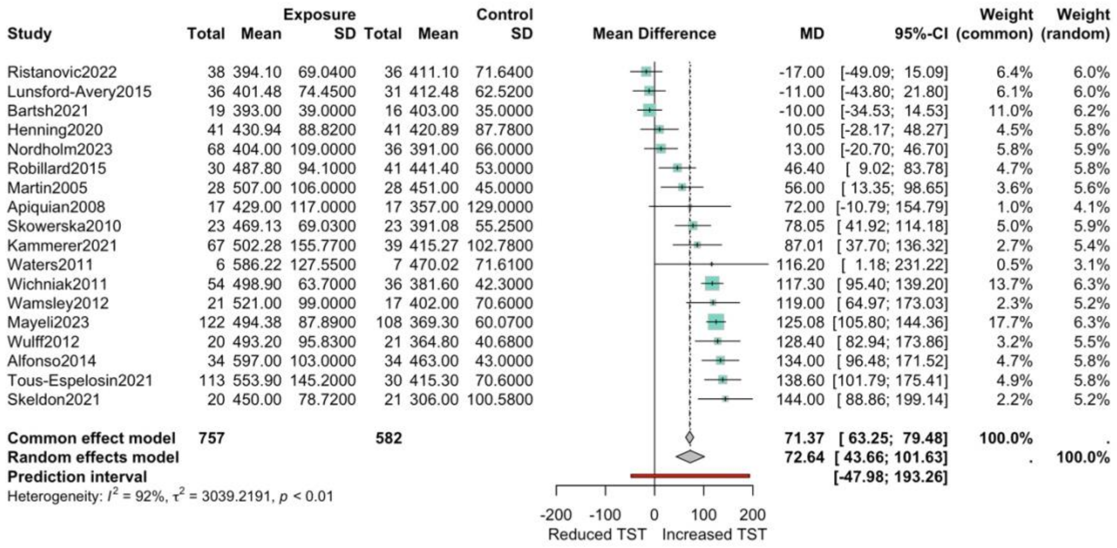

Figure 21

Review: Total Sleep time (TST)

```
                    MD             95%-CI %W(common) %W(random)
Alfonso2014        134.0000 [ 96.4826; 171.5174]        4.7        5.8
Apiquian2008        72.0000 [-10.7866; 154.7866]        1.0        4.1
Bartsh2021         -10.0000 [-34.5282;  14.5282]       11.0        6.2
Henning2020         10.0500 [-28.1743;  48.2743]        4.5        5.8
Kammerer2021        87.0100 [ 37.6976; 136.3224]        2.7        5.4
Lunsford-Avery2015 -11.0000 [-43.7997;  21.7997]        6.1        6.0
Mayeli2023         125.0800 [105.8037; 144.3563]       17.7        6.3
Martin2005          56.0000 [ 13.3463;  98.6537]        3.6        5.6
Nordholm2023        13.0000 [-20.7046;  46.7046]        5.8        5.9
Ristanovic2022     -17.0000 [-49.0859;  15.0859]        6.4        6.0
Robillard2015       46.4000 [  9.0231;  83.7769]        4.7        5.8
Skeldon2021        144.0000 [ 88.8566; 199.1434]        2.2        5.2
Skowerska2010       78.0500 [ 41.9154; 114.1846]        5.0        5.9
Tous-Espelosin2021 138.6000 [101.7902; 175.4098]        4.9        5.8
Wamsley2012        119.0000 [ 64.9707; 173.0293]        2.3        5.2
Waters2011         116.2000 [  1.1772; 231.2228]        0.5        3.1
Wichniak2011       117.3000 [ 95.4005; 139.1995]       13.7        6.3
Wulff2012          128.4000 [ 82.9401; 173.8599]        3.2        5.5
```

Number of studies: k = 18

Number of observations: o = 1339 (o.e = 757, o.c = 582)

```
                    MD             95%-CI  z|t  p-value
```

Common effect model 71.3655 [63.2469; 79.4841] 17.23 < 0.0001
Random effects model 72.6445 [43.6597; 101.6293] 5.29 < 0.0001
Prediction interval   [-47.9753; 193.2643]

Quantifying heterogeneity:
 tau^2 = 3039.2191 [1468.3390; 6930.7238]; tau = 55.1291 [38.3189; 83.2510]
 I^2 = 91.8% [88.5%; 94.1%]; H = 3.48 [2.95; 4.12]

Test of heterogeneity:
      Q d.f. p-value
 206.30   17 < 0.0001

Details on meta-analytical method:
- Inverse variance method
- Restricted maximum-likelihood estimator for tau^2
- Q-Profile method for confidence interval of tau^2 and tau
- Hartung-Knapp adjustment for random effects model (df = 17)
- Prediction interval based on t-distribution (df = 16)

Forest Plot, SSD studies

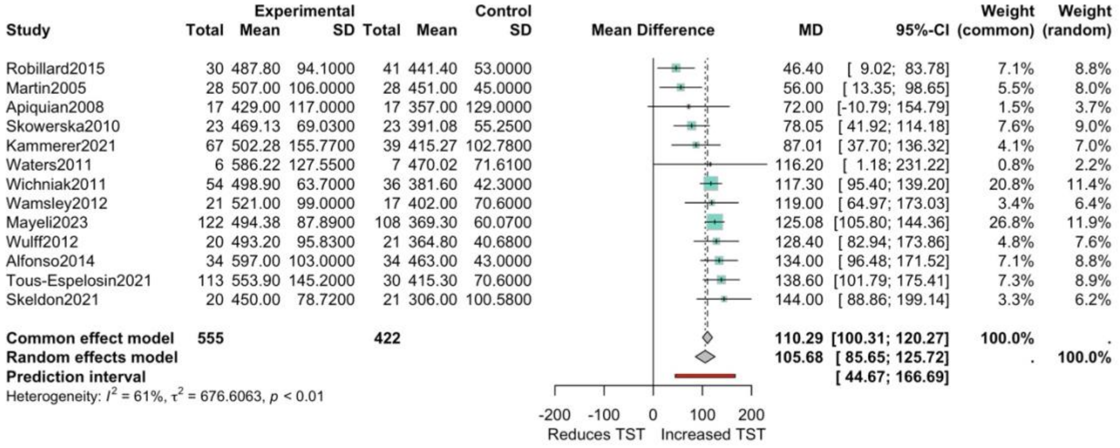

Figure 22

Review: CHR_TST

Number of studies: k = 13
Number of observations: o = 977 (o.e = 555, o.c = 422)

                  MD   95%-CI   z|t p-value
Common effect model 110.2913 [100.3133; 120.2693] 21.66 < 0.0001
Random effects model 105.6846 [85.6532; 125.7159] 11.50 < 0.0001
Prediction interval   [44.6748; 166.6943]

Quantifying heterogeneity:

tau^2 = 676.6063 [110.7701; 2392.3155]; tau = 26.0117 [10.5247; 48.9113]
I^2 = 61.0% [28.6%; 78.7%]; H = 1.60 [1.18; 2.17]

Test of heterogeneity:

Q	d.f.	p-value
30.80	12	0.0021

Details on meta-analytical method:
- Inverse variance method
- Restricted maximum-likelihood estimator for tau^2
- Q-Profile method for confidence interval of tau^2 and tau
- Hartung-Knapp adjustment for random effects model (df = 12)
- Prediction interval based on t-distribution (df = 11)

Three-arms study (Wichniak2011), only Risperidone arm

Forest Plot, all studies

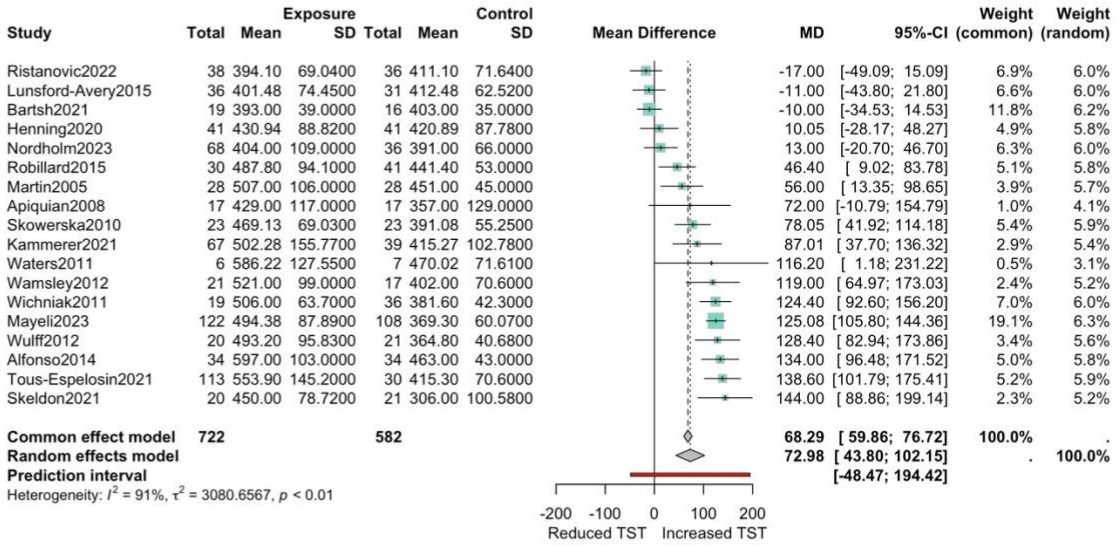

Figure 23

	MD	95%-CI	%W(common)	%W(random)
Alfonso2014	134.0000	[96.4826; 171.5174]	5.0	5.8
Apiquian2008	72.0000	[-10.7866; 154.7866]	1.0	4.1
Bartsh2021	-10.0000	[-34.5282; 14.5282]	11.8	6.2
Henning2020	10.0500	[-28.1743; 48.2743]	4.9	5.8
Kammerer2021	87.0100	[37.6976; 136.3224]	2.9	5.4
Lunsford-Avery2015	-11.0000	[-43.7997; 21.7997]	6.6	6.0
Mayeli2023	125.0800	[105.8037; 144.3563]	19.1	6.3
Martin2005	56.0000	[13.3463; 98.6537]	3.9	5.7
Nordholm2023	13.0000	[-20.7046; 46.7046]	6.3	6.0
Ristanovic2022	-17.0000	[-49.0859; 15.0859]	6.9	6.0
Robillard2015	46.4000	[9.0231; 83.7769]	5.1	5.8

Skeldon2021   144.0000 [88.8566; 199.1434]   2.3   5.2
Skowerska2010   78.0500 [41.9154; 114.1846]   5.4   5.9
Tous-Espelosin2021 138.6000 [101.7902; 175.4098]   5.2   5.9
Wamsley2012   119.0000 [64.9707; 173.0293]   2.4   5.2
Waters2011   116.2000 [1.1772; 231.2228]   0.5   3.1
Wichniak2011   124.4000 [92.5987; 156.2013]   7.0   6.0
Wulff2012   128.4000 [82.9401; 173.8599]   3.4   5.6

Number of studies: k = 18
Number of observations: o = 1304 (o.e = 722, o.c = 582)

MD   95%-CI   z|t p-value
Common effect model 68.2865 [59.8577; 76.7153] 15.88 < 0.0001
Random effects model 72.9753 [43.7963; 102.1542] 5.28 < 0.0001
Prediction interval   [-48.4671; 194.4176]

Quantifying heterogeneity:
tau^2 = 3080.6567 [1486.2668; 7015.6245]; tau = 55.5037 [38.5521; 83.7593]
I^2 = 91.5% [88.0%; 93.9%]; H = 3.43 [2.89; 4.06]

Test of heterogeneity:
Q d.f. p-value
199.57   17 < 0.0001

Details on meta-analytical method:
- Inverse variance method
- Restricted maximum-likelihood estimator for tau^2
- Q-Profile method for confidence interval of tau^2 and tau
- Hartung-Knapp adjustment for random effects model (df = 17)
- Prediction interval based on t-distribution (df = 16)

Forest Plot, SSD studies

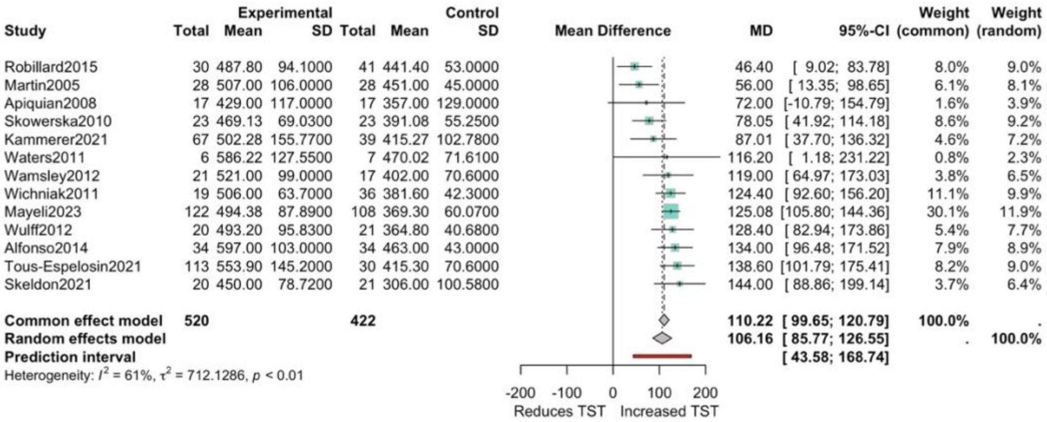

Figure 24

Number of studies: k = 13
Number of observations: o = 942 (o.e = 520, o.c = 422)

	MD	95%-CI	z|t	p-value
Common effect model	110.2172	[99.6456; 120.7888]	20.43	< 0.0001
Random effects model	106.1570	[85.7684; 126.5456]	11.34	< 0.0001
Prediction interval		[43.5752; 168.7388]		

Quantifying heterogeneity:
tau^2 = 712.1286 [118.0713; 2444.7301]; tau = 26.6857 [10.8661; 49.4442]
I^2 = 61.5% [29.6%; 78.9%]; H = 1.61 [1.19; 2.18]

Test of heterogeneity:

Q	d.f.	p-value
31.16	12	0.0019

Details on meta-analytical method:
- Inverse variance method
- Restricted maximum-likelihood estimator for tau^2
- Q-Profile method for confidence interval of tau^2 and tau
- Hartung-Knapp adjustment for random effects model (df = 12)
- Prediction interval based on t-distribution (df = 11)

SSD studies

Forest plot

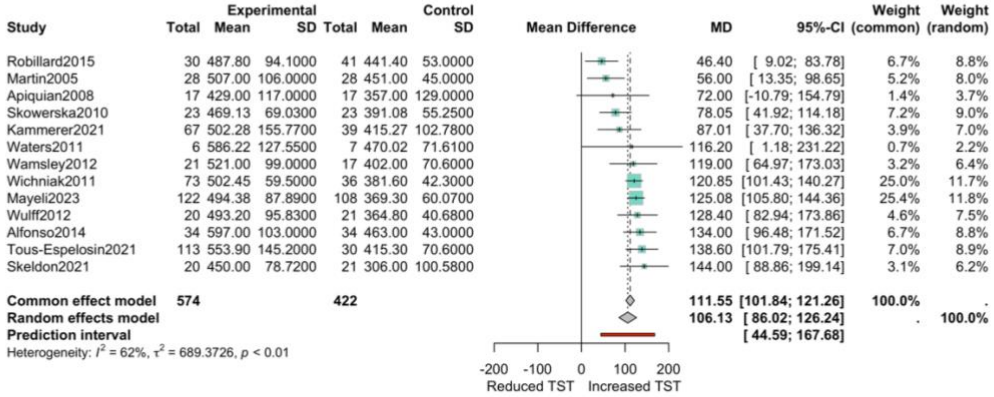

Figure 25

Number of studies: k = 13
Number of observations: o = 996 (o.e = 574, o.c = 422)

	MD	95%-CI	z|t	p-value
Common effect model	111.5520	[101.8437; 121.2603]	22.52	< 0.0001

Random effects model 106.1319 [86.0219; 126.2420] 1 1.50 < 0.0001
Prediction interval [44.5885; 167.6754]

Quantifying heterogeneity:
tau^2 = 689.3726 [119.0859; 2418.5656]; tau = 26.2559 [10.9127; 49.1789]
I^2 = 61.9% [30.4%; 79.1%]; H = 1.62 [1.20; 2.19]

Test of heterogeneity:
Q d.f. p-value
31.48 12 0.0017

Details on meta-analytical method:
- Inverse variance method
- Restricted maximum-likelihood estimator for tau^2
- Q-Profile method for confidence interval of tau^2 and tau
- Hartung-Knapp adjustment for random effects model (df = 12)
- Prediction interval based on t-distribution (df = 11)

Funnel plot

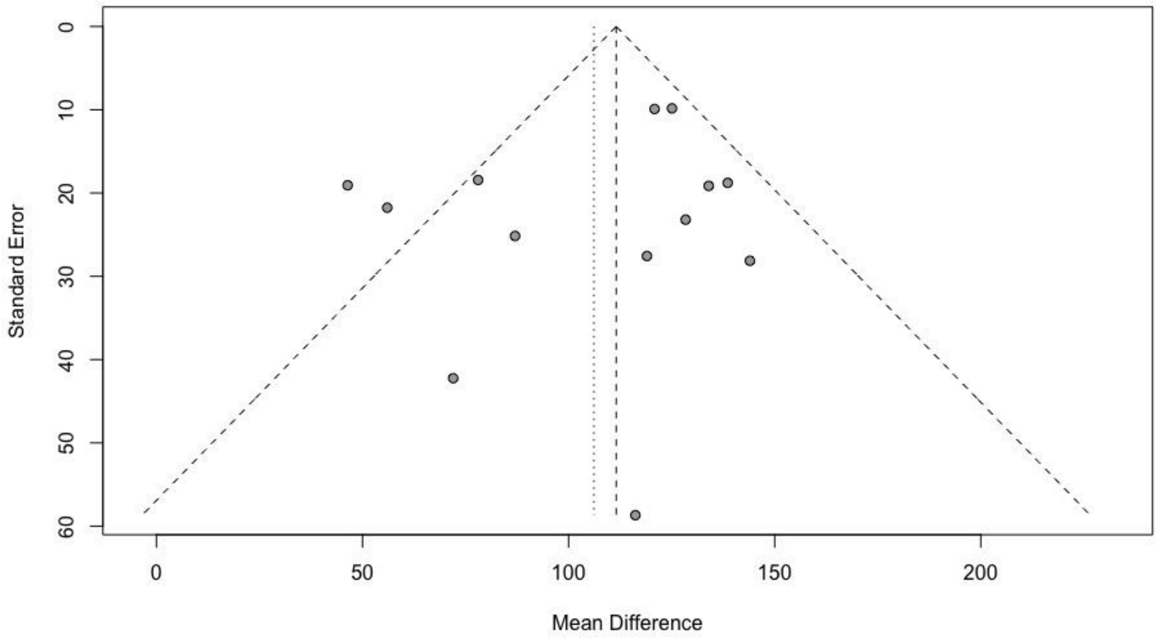

Figure 26

Linear regression test of funnel plot asymmetry

Test result: t = -1.01, df = 11, p-value = 0.3362
Bias estimate: -1.0426 (SE = 1.0367)

Details:
- multiplicative residual heterogeneity variance (tau^2 = 2.6206)

- predictor: standard error
- weight: inverse variance
- reference: Egger et al. (1997), BMJ

Eggers' test of the intercept
==============================
intercept   95% CI   t   p
-1.043 -3.07 - 0.99 -1.006 0.336159

Eggers' test does not indicate the presence of funnel plot asymmetry.

Outliers

Identified outliers (fixed-effect model)

"Martin2005", "Robillard2015"

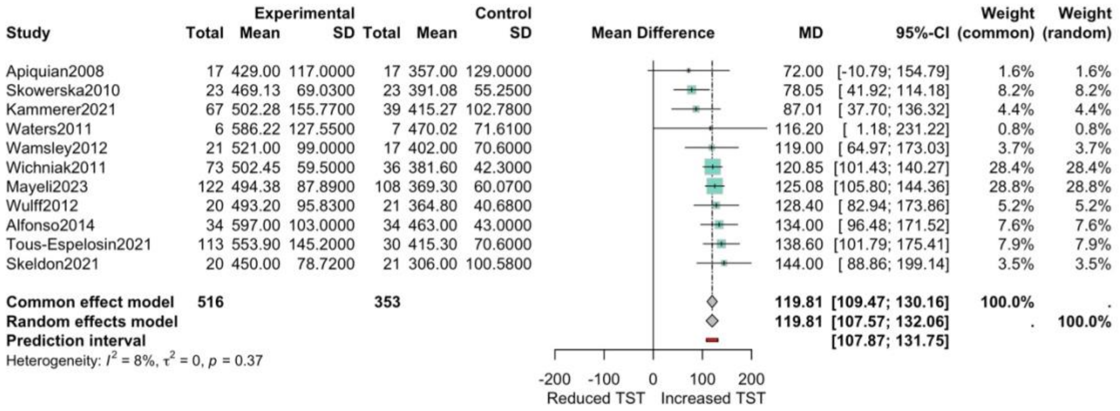

Figure 27

Results with outliers removed

Review: SSD_TST

Number of studies: k = 11
Number of observations: o = 869 (o.e = 516, o.c = 353)

	MD	95%-CI	z|t	p-value
Common effect model	119.8103	[109.4656; 130.1551]	22.70	< 0.0001
Random effects model	119.8103	[107.5656; 132.0551]	21.80	< 0.0001
Prediction interval		[107.8706; 131.7501]		

Quantifying heterogeneity:
tau^2 = 0 [0.0000; >100.0000]; tau = 0 [0.0000; >10.0000]
I^2 = 7.8% [0.0%; 63.3%]; H = 1.04 [1.00; 1.65]

Test of heterogeneity:
Q d.f. p-value
10.84 10 0.3700

Details on meta-analytical method:
- Inverse variance method
- Restricted maximum-likelihood estimator for tau^2
- Q-Profile method for confidence interval of tau^2 and tau
- Hartung-Knapp adjustment for random effects model (df = 10)
- Prediction interval based on t-distribution (df = 9)

Identified outliers (random-effects model)

"Robillard2015"

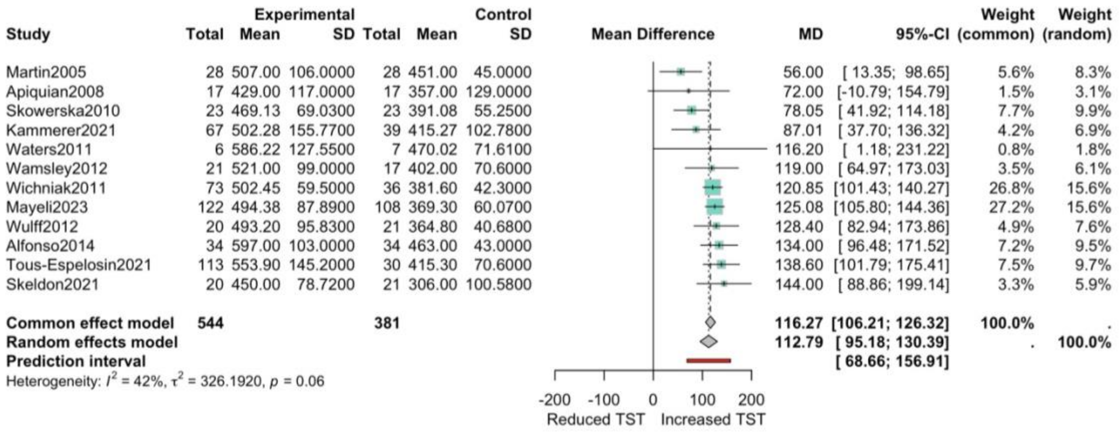

Figure 28

Results with outliers removed

Review: SSD_TST

Number of studies: k = 12
Number of observations: o = 925 (o.e = 544, o.c = 381)

	MD	95%-CI	z|t	p-value
Common effect model	116.2655	[106.2122; 126.3188]	22.67	< 0.0001
Random effects model	112.7857	[95.1798; 130.3915]	14.10	< 0.0001
Prediction interval		[68.6580; 156.9133]		

Quantifying heterogeneity:
tau^2 = 326.1920 [0.0000; 1810.2295]; tau = 18.0608 [0.0000; 42.5468]
I^2 = 42.0% [0.0%; 70.5%]; H = 1.31 [1.00; 1.84]

Test of heterogeneity:

Q	d.f.	p-value
18.96	11	0.0618

Details on meta-analytical method:
- Inverse variance method
- Restricted maximum-likelihood estimator for tau^2
- Q-Profile method for confidence interval of tau^2 and tau
- Hartung-Knapp adjustment for random effects model (df = 11)
- Prediction interval based on t-distribution (df = 10)

Baujat Plot

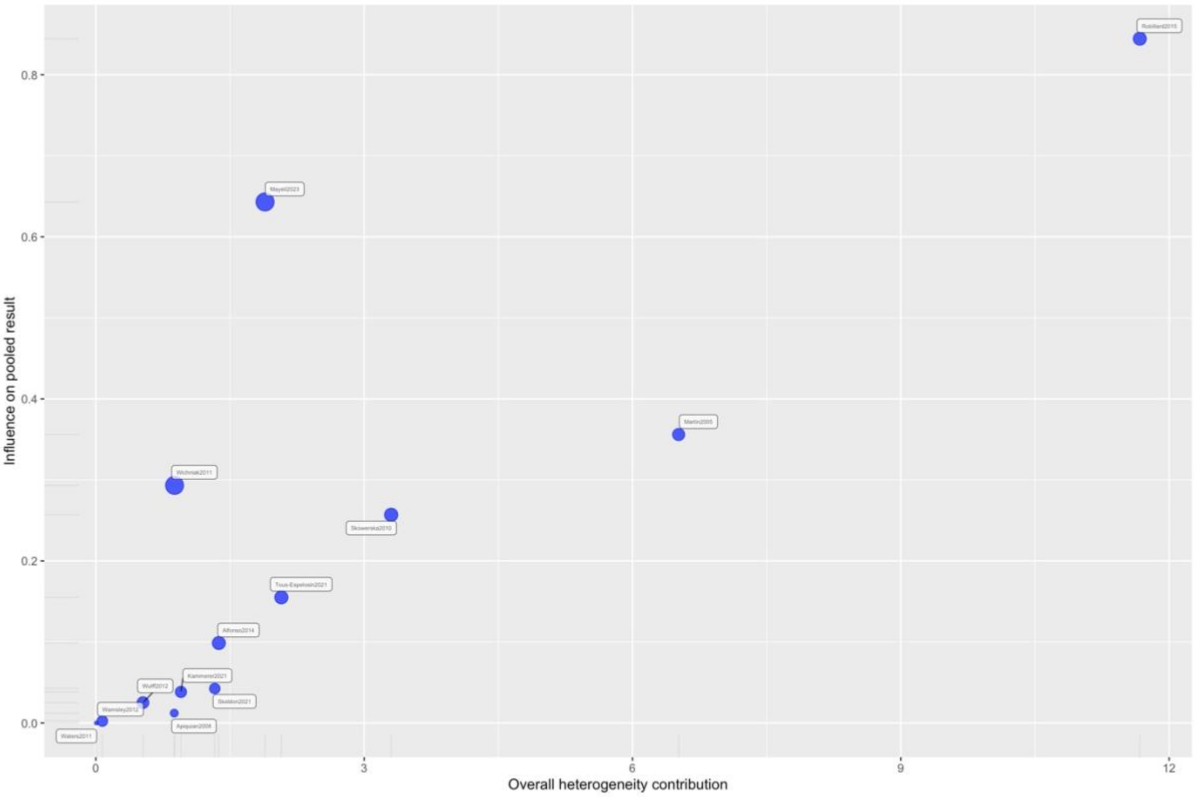

Figure 29

Influence Analyses

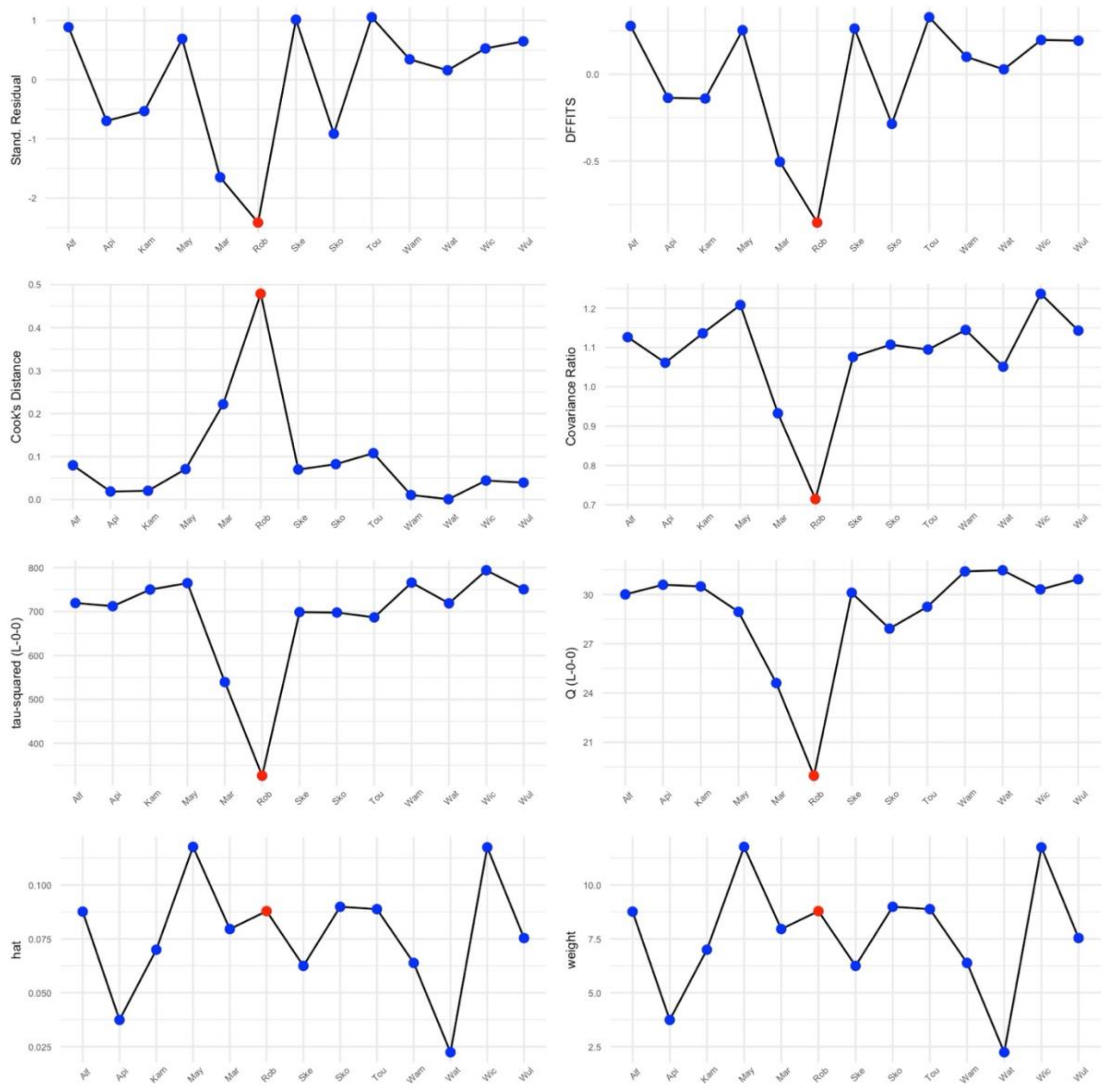

Figure 30

Meta-regressions

Year

Mixed-Effects Model (k = 13; tau^2 estimator: REML)

tau^2 (estimated amount of residual heterogeneity): 555.4561 (SE = 435.3325)
tau (square root of estimated tau^2 value): 23.5681
I^2 (residual heterogeneity / unaccounted variability): 58.27%
H^2 (unaccounted variability / sampling variability): 2.40
R^2 (amount of heterogeneity accounted for): 19.43%

Test for Residual Heterogeneity:
QE(df = 11) = 25.5465, p-val = 0.0076

Test of Moderators (coefficient 2):
F(df1 = 1, df2 = 11) = 3.2111, p-val = 0.1007

Model Results:

	estimate	se	tval	df	pval	ci.lb	ci.ub
intrcpt	-5367.1163	3054.4692	-1.7571	11	0.1067	-12089.9578	1355.7251
year	2.7167	1.5161	1.7920	11	0.1007	-0.6201	6.0535

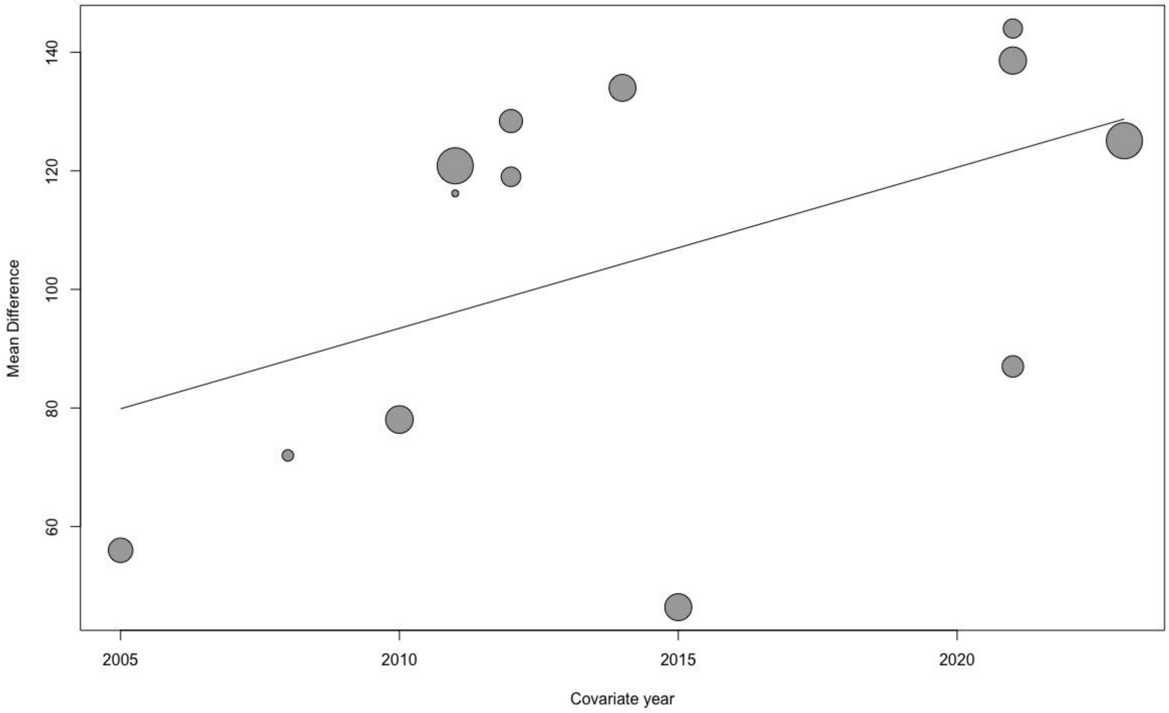

Figure 31

Percentage treated with antipsychotics

Mixed-Effects Model (k = 11; tau^2 estimator: REML)

tau^2 (estimated amount of residual heterogeneity): 418.8128 (SE = 379.3428)
tau (square root of estimated tau^2 value): 20.4649
I^2 (residual heterogeneity / unaccounted variability): 57.04%
H^2 (unaccounted variability / sampling variability): 2.33
R^2 (amount of heterogeneity accounted for): 24.06%

Test for Residual Heterogeneity:
QE(df = 9) = 17.7691, p-val = 0.0379

Test of Moderators (coefficient 2):

F(df1 = 1, df2 = 9) = 2.1765, p-val = 0.1742

Model Results:

	estimate	se	tval	df	pval	ci.lb	ci.ub
intrcpt	24.7289	59.9494	0.4125	9	0.6896	-110.8861	160.3439
Percentage	94.6196	64.1360	1.4753	9	0.1742	-50.4660	239.7053

Signif. codes: 0 ‘***’ 0.001 ‘**’ 0.01 ‘*’ 0.05 ‘.’ 0.1 ‘ ’ 1

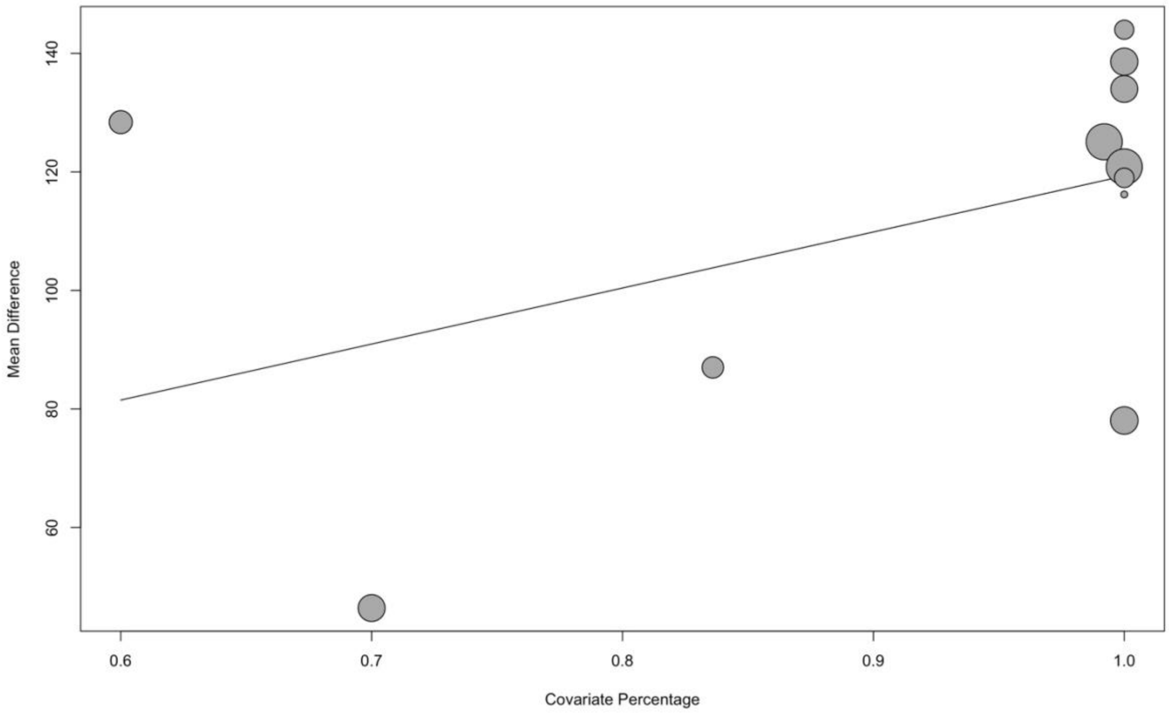

Figure 32

Age

Mixed-Effects Model (k = 13; tau^2 estimator: REML)

tau^2 (estimated amount of residual heterogeneity): 797.2596 (SE = 546.9101)
tau (square root of estimated tau^2 value): 28.2358
I^2 (residual heterogeneity / unaccounted variability): 68.40%
H^2 (unaccounted variability / sampling variability): 3.16
R^2 (amount of heterogeneity accounted for): 0.00%

Test for Residual Heterogeneity:
QE(df = 11) = 31.4750, p-val = 0.0009

Test of Moderators (coefficient 2):
F(df1 = 1, df2 = 11) = 0.0001, p-val = 0.9924

Model Results:

	estimate	se	tval	df	pval	ci.lb	ci.ub	
intrcpt	105.5906	43.8417	2.4084	11	0.0347	9.0956	202.0856	*
age_mean_e	0.0111	1.1404	0.0097	11	0.9924	-2.4988	2.5210	

Signif. codes: 0 '***' 0.001 '**' 0.01 '*' 0.05 '.' 0.1 ' ' 1

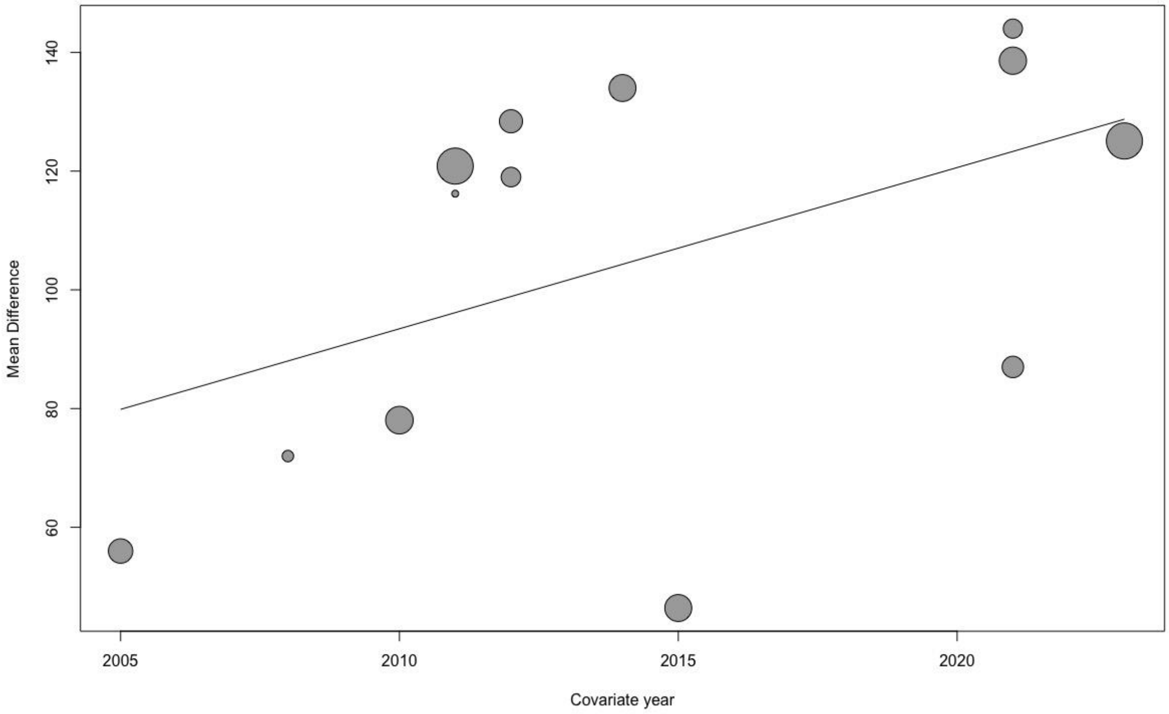

Figure 33

Gender_female

Mixed-Effects Model (k = 13; tau^2 estimator: REML)

tau^2 (estimated amount of residual heterogeneity): 551.9245 (SE = 424.7593)
tau (square root of estimated tau^2 value): 23.4931
I^2 (residual heterogeneity / unaccounted variability): 61.66%
H^2 (unaccounted variability / sampling variability): 2.61
R^2 (amount of heterogeneity accounted for): 19.94%

Test for Residual Heterogeneity:
QE(df = 11) = 25.5560, p-val = 0.0076

Test of Moderators (coefficient 2):

F(df1 = 1, df2 = 11) = 2.7746, p-val = 0.1240

Model Results:

	estimate	se	tval	df	pval	ci.lb	ci.ub	
intrcpt	148.6589	26.8141	5.5441	11	0.0002	89.6414	207.6764	***
female_percentage_e	-112.8103	67.7246	-1.6657	11	0.1240	-261.8712	36.2506	

Signif. codes: 0 '***' 0.001 '**' 0.01 '*' 0.05 '.' 0.1 ' ' 1

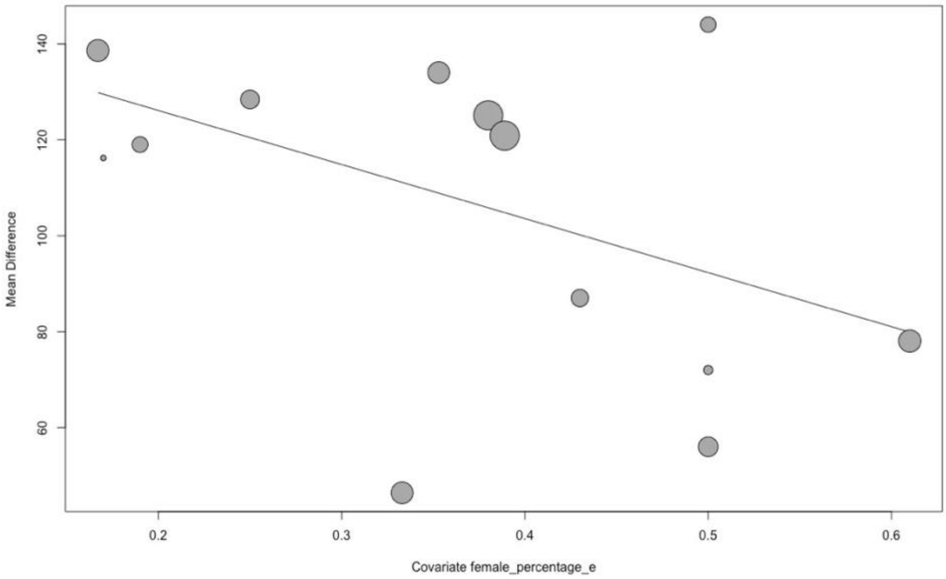

Figure 34

PANSS score

Mixed-Effects Model (k = 5; tau^2 estimator: REML)

tau^2 (estimated amount of residual heterogeneity): 0 (SE = 217.8419)
tau (square root of estimated tau^2 value): 0
I^2 (residual heterogeneity / unaccounted variability): 0.00%
H^2 (unaccounted variability / sampling variability): 1.00
R^2 (amount of heterogeneity accounted for): 100.00%

Test for Residual Heterogeneity:
QE(df = 3) = 2.4951, p-val = 0.4762

Test of Moderators (coefficient 2):
F(df1 = 1, df2 = 3) = 4.8623, p-val = 0.1146

Model Results:

```
            estimate      se    tval  df    pval    ci.lb     ci.ub
intrcpt     65.1629  22.7228  2.8677   3  0.0642  -7.1510  137.4769  .
PANSS_mean    1.1668   0.5292  2.2051   3  0.1146  -0.5172    2.8509
```

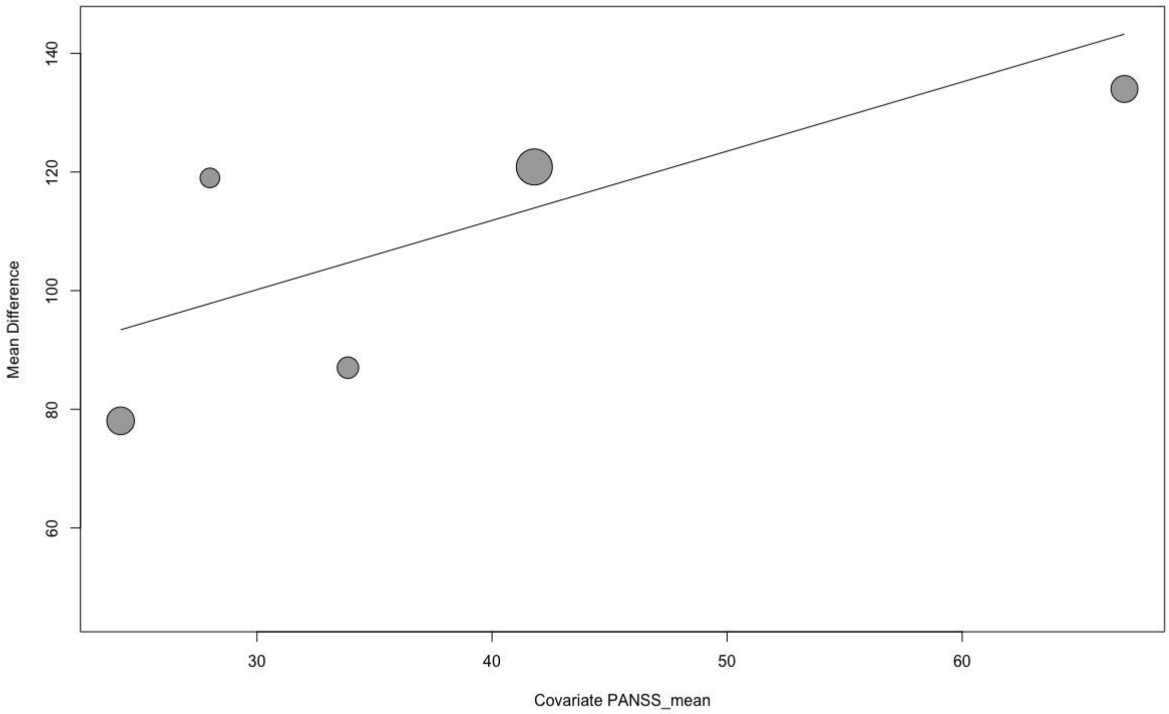

Figure 35

Study sample

Mixed-Effects Model (k = 13; tau^2 estimator: REML)

tau^2 (estimated amount of residual heterogeneity): 559.5066 (SE = 439.7281)
tau (square root of estimated tau^2 value): 23.6539
I^2 (residual heterogeneity / unaccounted variability): 59.35%
H^2 (unaccounted variability / sampling variability): 2.46
R^2 (amount of heterogeneity accounted for): 18.84%

Test for Residual Heterogeneity:
QE(df = 11) = 23.7226, p-val = 0.0140

Test of Moderators (coefficient 2):
F(df1 = 1, df2 = 11) = 1.9947, p-val = 0.1855

Model Results:

estimate se tval df pval ci.lb ci.ub
intrcpt 89.0138 15.1199 5.8872 11 0.0001 55.7351 122.2925 ***
n_e 0.3247 0.2299 1.4123 11 0.1855 -0.1813 0.8306

Signif. codes: 0 ‘***’ 0.001 ‘**’ 0.01 ‘*’ 0.05 ‘.’ 0.1 ‘ ’ 1

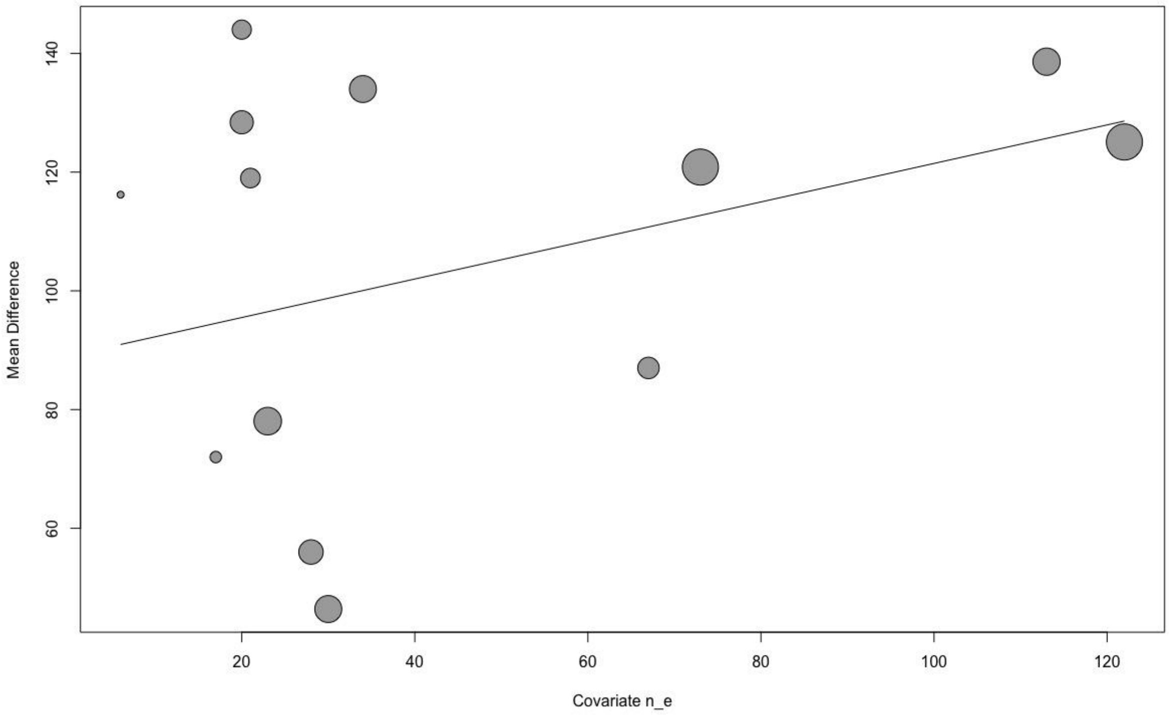

Figure 36

Epochs

Mixed-Effects Model (k = 11; tau^2 estimator: REML)

tau^2 (estimated amount of residual heterogeneity): 950.3904 (SE = 719.5626)
tau (square root of estimated tau^2 value): 30.8284
I^2 (residual heterogeneity / unaccounted variability): 67.68%
H^2 (unaccounted variability / sampling variability): 3.09
R^2 (amount of heterogeneity accounted for): 0.00%

Test for Residual Heterogeneity:
QE(df = 9) = 26.1523, p-val = 0.0019

Test of Moderators (coefficient 2):
F(df1 = 1, df2 = 9) = 0.0630, p-val = 0.8075

Model Results:

estimate se tval df pval ci.lb ci.ub
intrcpt 102.4333 19.1843 5.3394 9 0.0005 59.0353 145.8312 ***
Epochs_seconds 0.0745 0.2968 0.2509 9 0.8075 -0.5970 0.7459

Signif. codes: 0 '***' 0.001 '**' 0.01 '*' 0.05 '.' 0.1 ' ' 1

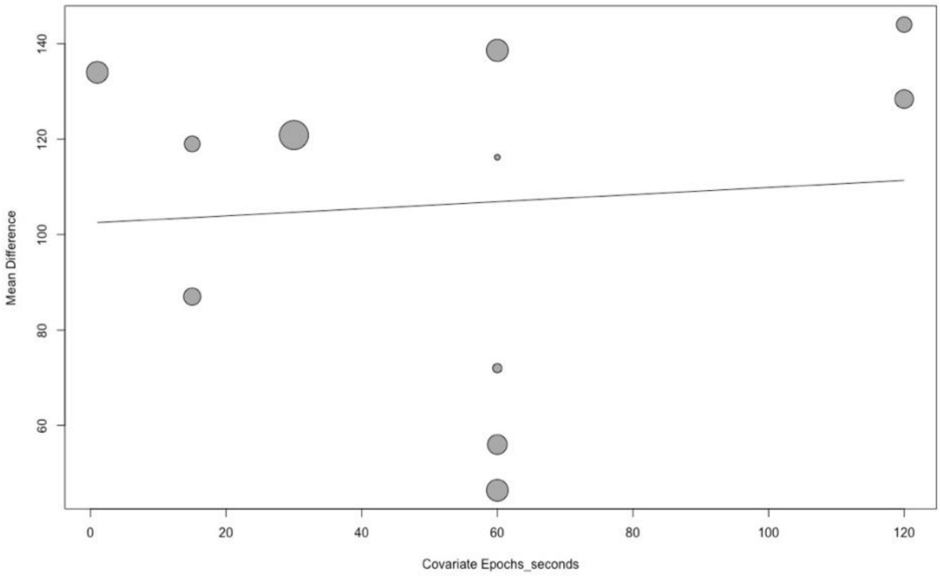

Figure 37

Follow-up time - mean

Mixed-Effects Model (k = 12; tau^2 estimator: REML)

tau^2 (estimated amount of residual heterogeneity): 759.4413 (SE = 553.5469)
tau (square root of estimated tau^2 value): 27.5580
I^2 (residual heterogeneity / unaccounted variability): 68.83%
H^2 (unaccounted variability / sampling variability): 3.21
R^2 (amount of heterogeneity accounted for): 0.00%

Test for Residual Heterogeneity:
QE(df = 10) = 27.4541, p-val = 0.0022

Test of Moderators (coefficient 2):
F(df1 = 1, df2 = 10) = 0.8639, p-val = 0.3745

Model Results:

estimate se tval df pval ci.lb ci.ub
intrcpt 100.4468 13.2884 7.5590 10 <.0001 70.8385 130.0552
Follow_up_actigraphy_days_mean 0.6834 0.7353 0.9295 10 0.3745 -0.9549
2.3218

intrcpt   ***
Follow_up_actigraphy_days_mean

Signif. codes: 0 ‘***’ 0.001 ‘**’ 0.01 ‘*’ 0.05 ‘.’ 0.1 ‘ ’ 1

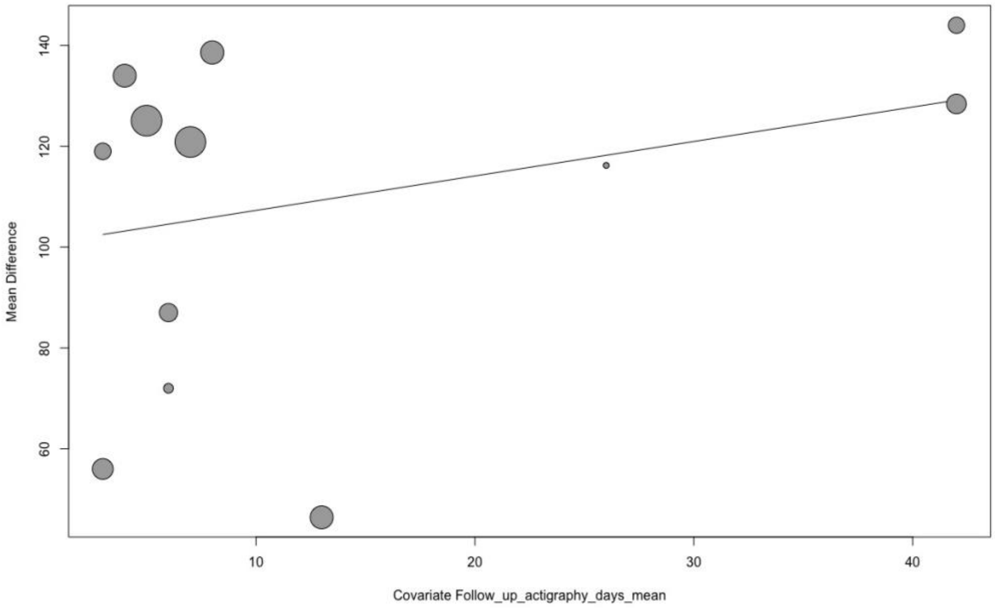

Figure 38

Follow-up time - max

Mixed-Effects Model (k = 12; tau^2 estimator: REML)

tau^2 (estimated amount of residual heterogeneity):   804.0527 (SE = 576.9404)
tau (square root of estimated tau^2 value):   28.3558
I^2 (residual heterogeneity / unaccounted variability): 69.91%
H^2 (unaccounted variability / sampling variability):   3.32
R^2 (amount of heterogeneity accounted for):   0.00%

Test for Residual Heterogeneity:
QE(df = 10) = 27.9167, p-val = 0.0019

Test of Moderators (coefficient 2):
F(df1 = 1, df2 = 10) = 0.3778, p-val = 0.5525

Model Results:

	estimate	se	tval	df	pval	ci.lb	ci.ub
intrcpt	102.3188	14.4798	7.0663	10	<.0001	70.0558	134.5818
Follow_up_actigraphy_days_max	0.4633	0.7537	0.6147	10	0.5525	-1.2160	2.1426

intrcpt   ***
Follow_up_actigraphy_days_max

Signif. codes: 0 ‘***’ 0.001 ‘**’ 0.01 ‘*’ 0.05 ‘.’ 0.1 ‘ ’ 1

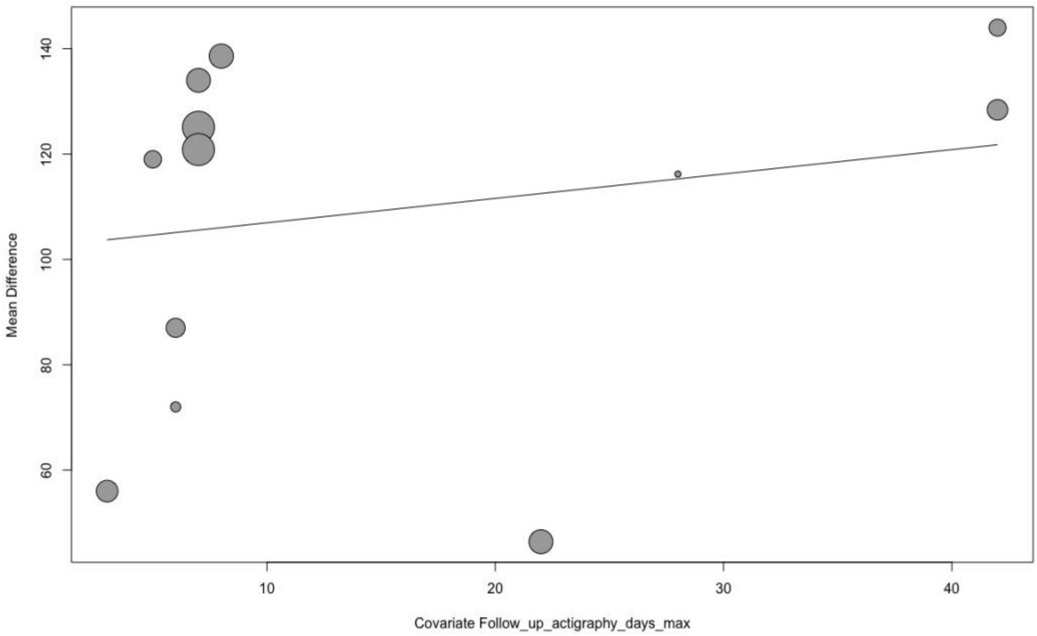

Figure 39

Subgroup analyses

SSD_Epochs < 60 sec

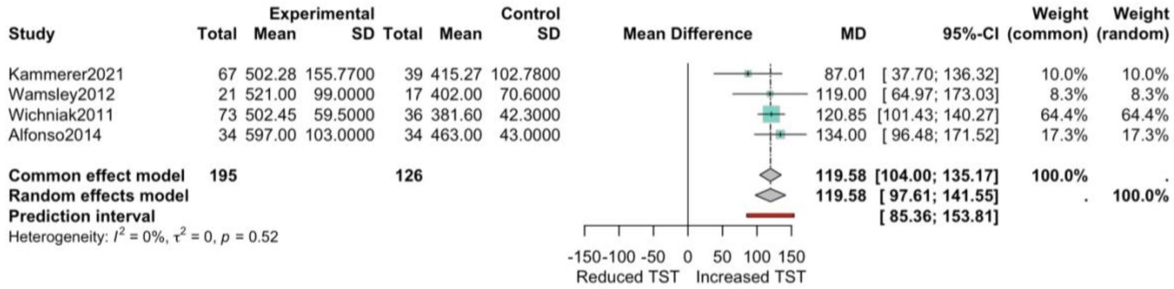

Figure 40

Number of studies: k = 4
Number of observations: o = 321 (o.e = 195, o.c = 126)

MD 95%-CI z|t p-value
Common effect model 119.5846 [103.9960; 135.1732] 15.04 < 0.0001
Random effects model 119.5846 [97.6147; 141.5544] 17.32 0.0004
Prediction interval [85.3634; 153.8057]

Quantifying heterogeneity:
tau^2 = 0 [0.0000; >100.0000]; tau = 0 [0.0000; >10.0000]
I^2 = 0.0% [0.0%; 84.7%]; H = 1.00 [1.00; 2.56]

Test of heterogeneity:
Q d.f. p-value
2.26 3 0.5202

Details on meta-analytical method:
- Inverse variance method
- Restricted maximum-likelihood estimator for tau^2
- Q-Profile method for confidence interval of tau^2 and tau
- Hartung-Knapp adjustment for random effects model (df = 3)
- Prediction interval based on t-distribution (df = 2)

SSD_Epochs > or = 60 sec

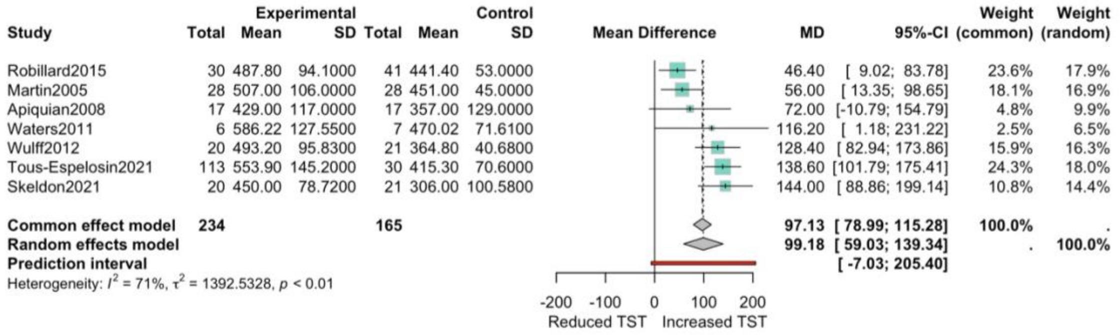

Figure 41

Number of studies: k = 7
Number of observations: o = 399 (o.e = 234, o.c = 165)

MD 95%-CI z|t p-value
Common effect model 97.1319 [78.9882; 115.2756] 10.49 < 0.0001
Random effects model 99.1846 [59.0340; 139.3353] 6.04 0.0009
Prediction interval [-7.0345; 205.4037]

Quantifying heterogeneity:
tau^2 = 1392.5328 [186.1690; 7518.2374]; tau = 37.3167 [13.6444; 86.7078]
I^2 = 70.8% [36.4%; 86.6%]; H = 1.85 [1.25; 2.73]

Test of heterogeneity:
Q d.f. p-value
20.58 6 0.0022

Details on meta-analytical method:
- Inverse variance method
- Restricted maximum-likelihood estimator for tau^2
- Q-Profile method for confidence interval of tau^2 and tau
- Hartung-Knapp adjustment for random effects model (df = 6)
- Prediction interval based on t-distribution (df = 5)

SSD_Follow-up: < 7 days

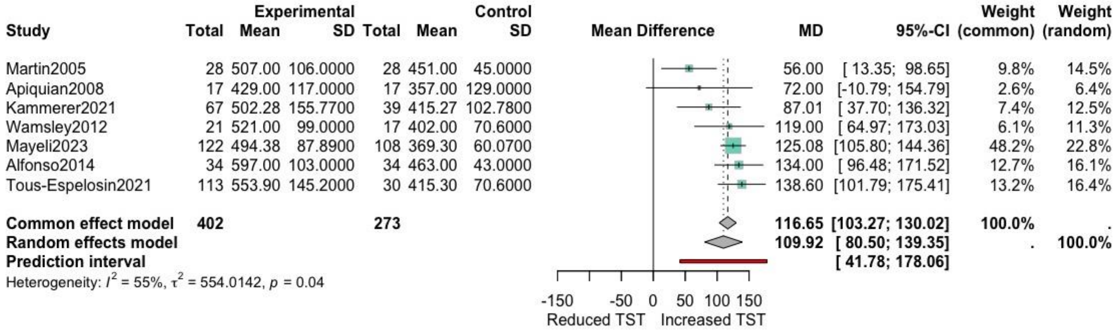

Figure 42

Number of studies: k = 7
Number of observations: o = 675 (o.e = 402, o.c = 273)

	MD	95%-CI	z|t	p-value
Common effect model	116.6461	[103.2700; 130.0222]	17.09	< 0.0001
Random effects model	109.9215	[80.4970; 139.3460]	9.14	< 0.0001
Prediction interval		[41.7843; 178.0586]		

Quantifying heterogeneity:
tau^2 = 554.0142 [0.0000; 4544.9766]; tau = 23.5375 [0.0000; 67.4164]
I^2 = 54.6% [0.0%; 80.5%]; H = 1.48 [1.00; 2.27]

Test of heterogeneity:
Q d.f. p-value
13.20 6 0.0399

Details on meta-analytical method:
- Inverse variance method
- Restricted maximum-likelihood estimator for tau^2
- Q-Profile method for confidence interval of tau^2 and tau
- Hartung-Knapp adjustment for random effects model (df = 6)
- Prediction interval based on t-distribution (df = 5)

SSD_Follow-up: > or = 7 days

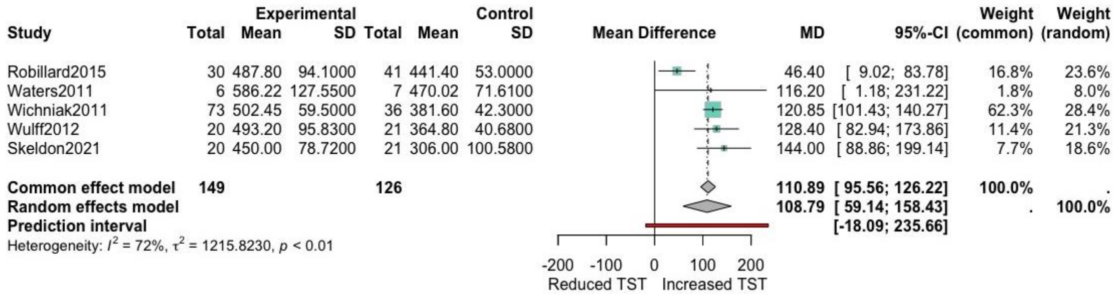

Figure 43

Number of studies: k = 5
Number of observations: o = 275 (o.e = 149, o.c = 126)

	MD	95%-CI	z|t	p-value
Common effect model	110.8909	[95.5607; 126.2211]	14.18	< 0.0001
Random effects model	108.7862	[59.1409; 158.4314]	6.08	0.0037
Prediction interval		[-18.0879; 235.6602]		

Quantifying heterogeneity:
tau^2 = 1215.8230 [99.7574; 11283.4944]; tau = 34.8687 [9.9879; 106.2238]
I^2 = 72.2% [30.2%; 89.0%]; H = 1.90 [1.20; 3.01]

Test of heterogeneity:

Q	d.f.	p-value
14.41	4	0.0061

Details on meta-analytical method:
- Inverse variance method
- Restricted maximum-likelihood estimator for tau^2
- Q-Profile method for confidence interval of tau^2 and tau
- Hartung-Knapp adjustment for random effects model (df = 4)
- Prediction interval based on t-distribution (df = 3)

SSD – chronic schizophrenia

Forest Plot

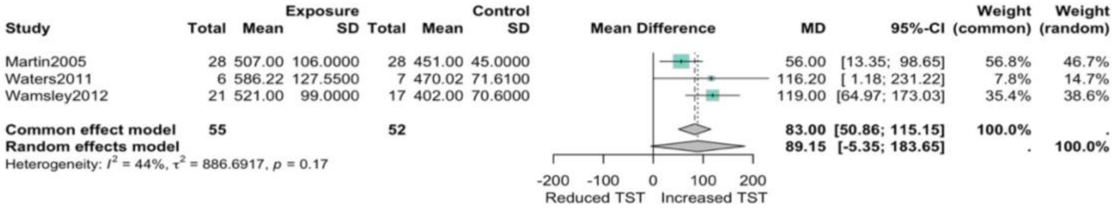

Figure 44

MD 95%-CI %W(common) %W(random)
Martin2005 56.0000 [13.3463; 98.6537] 56.8 46.7
Wamsley2012 119.0000 [64.9707; 173.0293] 35.4 38.6
Waters2011 116.2000 [1.1772; 231.2228] 7.8 14.7

Number of studies: k = 3
Number of observations: o = 107 (o.e = 55, o.c = 52)

MD 95%-CI z|t p-value
Common effect model 83.0012 [50.8566; 115.1458] 5.06 < 0.0001
Random effects model 89.1529 [-5.3479; 183.6537] 4.06 0.0557

Quantifying heterogeneity:
tau^2 = 886.6917 [0.0000; >8866.9168]; tau = 29.7774 [0.0000; >94.1643]
I^2 = 43.9% [0.0%; 83.2%]; H = 1.34 [1.00; 2.44]

Test of heterogeneity:
Q d.f. p-value
3.56 2 0.1682

Details on meta-analytical method:
- Inverse variance method
- Restricted maximum-likelihood estimator for tau^2
- Q-Profile method for confidence interval of tau^2 and tau
- Hartung-Knapp adjustment for random effects model (df = 2)

CHR-P studies

Forest Plot

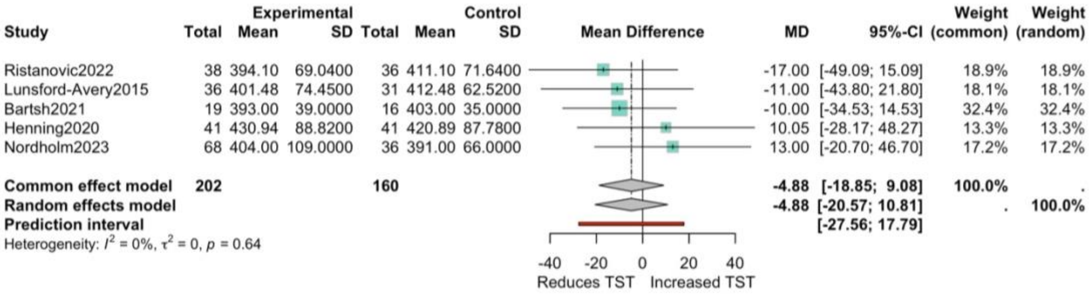

Figure 45

Number of studies: k = 5
Number of observations: o = 362 (o.e = 202, o.c = 160)

MD 95%-CI z|t p-value

Common effect model -4.8827 [-18.8476; 9.0822] -0.69 0.4932
Random effects model -4.8827 [-20.5731; 10.8078] -0.86 0.4363
Prediction interval [-27.5579; 17.7925]

Quantifying heterogeneity:
tau^2 = 0 [0.0000; >100.0000]; tau = 0 [0.0000; >10.0000]
I^2 = 0.0% [0.0%; 79.2%]; H = 1.00 [1.00; 2.19]

Test of heterogeneity:
Q d.f. p-value
2.52 4 0.6417

Details on meta-analytical method:
- Inverse variance method
- Restricted maximum-likelihood estimator for tau^2
- Q-Profile method for confidence interval of tau^2 and tau
- Hartung-Knapp adjustment for random effects model (df = 4)
- Prediction interval based on t-distribution (df = 3)

Funnel plot

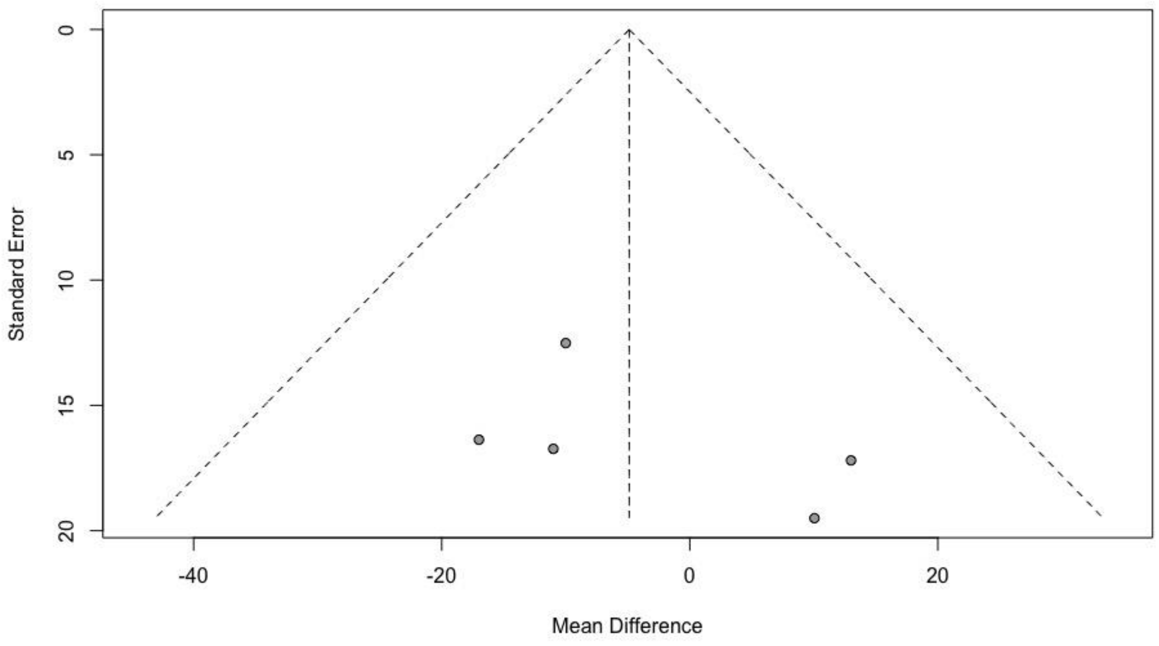

Figure 46

Outliers

No outliers detected (fixed-effect/random-effects model).

Baujat Plot

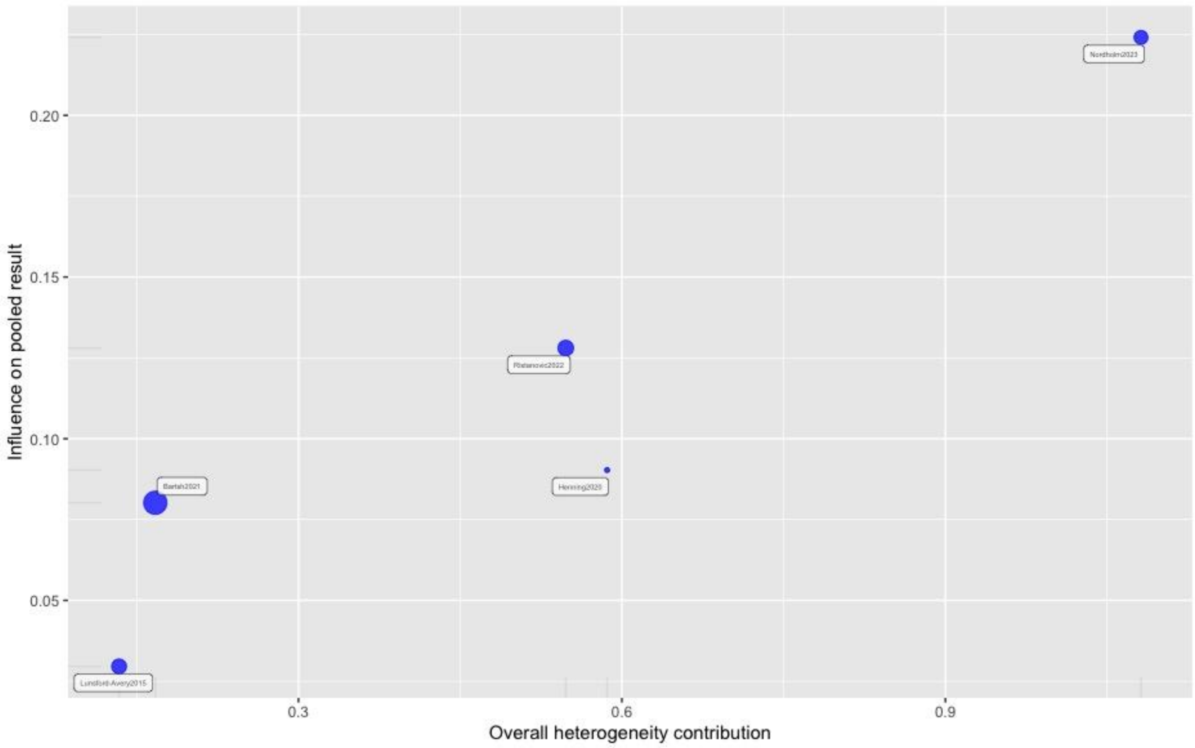

Figure 47

Influence analyses

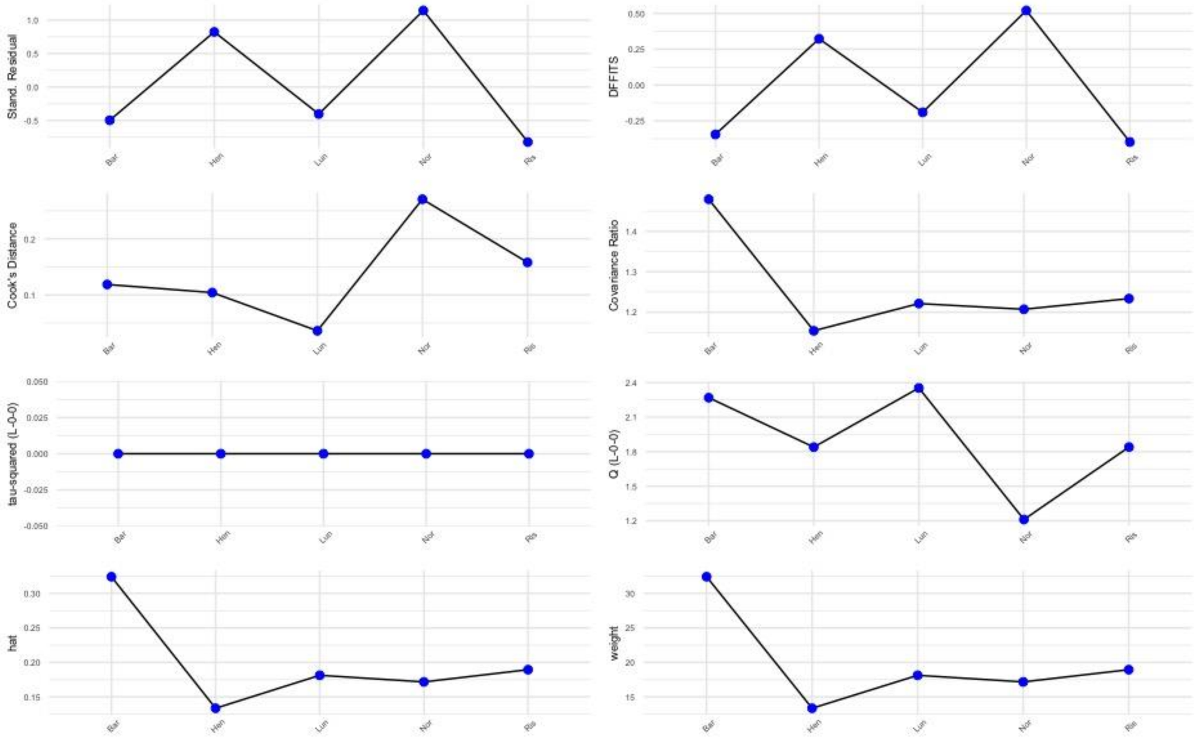

Figure 48

Meta-regressions

Year

Mixed-Effects Model (k = 5; tau^2 estimator: REML)

tau^2 (estimated amount of residual heterogeneity): 0 (SE = 201.2443)
tau (square root of estimated tau^2 value): 0
I^2 (residual heterogeneity / unaccounted variability): 0.00%
H^2 (unaccounted variability / sampling variability): 1.00
R^2 (amount of heterogeneity accounted for): 0.00%

Test for Residual Heterogeneity:
QE(df = 3) = 2.3146, p-val = 0.5097

Test of Moderators (coefficient 2):
F(df1 = 1, df2 = 3) = 0.2615, p-val = 0.6443

Model Results:

	estimate	se	tval	df	pval	ci.lb	ci.ub
intrcpt	-2438.0965	4758.0895	-0.5124	3	0.6437	-17580.4609	12704.2679
year	1.2044	2.3551	0.5114	3	0.6443	-6.2907	8.6994

Age_mean

Mixed-Effects Model (k = 4; tau^2 estimator: REML)

tau^2 (estimated amount of residual heterogeneity): 0 (SE = 306.8561)
tau (square root of estimated tau^2 value): 0
I^2 (residual heterogeneity / unaccounted variability): 0.00%
H^2 (unaccounted variability / sampling variability): 1.00
R^2 (amount of heterogeneity accounted for): 0.00%

Test for Residual Heterogeneity:
QE(df = 2) = 0.3692, p-val = 0.8314

Test of Moderators (coefficient 2):
F(df1 = 1, df2 = 2) = 10.2901, p-val = 0.0850

Model Results:

	estimate	se	tval	df	pval	ci.lb	ci.ub	
intrcpt	-117.1964	35.9709	-3.2581	2	0.0827	-271.9665	37.5738	.
age_mean_e	5.5805	1.7396	3.2078	2	0.0850	-1.9046	13.0656	.

Signif. codes: 0 ‘***’ 0.001 ‘**’ 0.01 ‘*’ 0.05 ‘.’ 0.1 ‘ ’ 1

Gender_female

Mixed-Effects Model (k = 4; tau^2 estimator: REML)

tau^2 (estimated amount of residual heterogeneity): 0 (SE = 302.9400)
tau (square root of estimated tau^2 value): 0
I^2 (residual heterogeneity / unaccounted variability): 0.00%
H^2 (unaccounted variability / sampling variability): 1.00
R^2 (amount of heterogeneity accounted for): 0.00%

Test for Residual Heterogeneity:
QE(df = 2) = 0.0916, p-val = 0.9552

Test of Moderators (coefficient 2):
F(df1 = 1, df2 = 2) = 47.5214, p-val = 0.0204

Model Results:

	estimate	se	tval	df	pval	ci.lb	ci.ub	
intrcpt	-81.5836	11.6314	-7.0141	2	0.0197	-131.6293	-31.5378	*
female_percentage_e	157.0029	22.7752	6.8936	2	0.0204	59.0089	254.9968	*

Signif. codes: 0 ‘***’ 0.001 ‘**’ 0.01 ‘*’ 0.05 ‘.’ 0.1 ‘ ’ 1

Study_sample

Mixed-Effects Model (k = 5; tau^2 estimator: REML)

tau^2 (estimated amount of residual heterogeneity): 0 (SE = 217.8419)
tau (square root of estimated tau^2 value): 0
I^2 (residual heterogeneity / unaccounted variability): 0.00%
H^2 (unaccounted variability / sampling variability): 1.00
R^2 (amount of heterogeneity accounted for): 100.00%

Test for Residual Heterogeneity:
QE(df = 3) = 2.4951, p-val = 0.4762

Test of Moderators (coefficient 2):
F(df1 = 1, df2 = 3) = 4.8623, p-val = 0.1146

Model Results:

	estimate	se	tval	df	pval	ci.lb	ci.ub	
intrcpt	65.1629	22.7228	2.8677	3	0.0642	-7.1510	137.4769	.
PANSS_mean	1.1668	0.5292	2.2051	3	0.1146	-0.5172	2.8509	

Signif. codes: 0 ‘***’ 0.001 ‘**’ 0.01 ‘*’ 0.05 ‘.’ 0.1 ‘ ’ 1

Epochs

Mixed-Effects Model (k = 4; tau^2 estimator: REML)

tau^2 (estimated amount of residual heterogeneity): 0 (SE = 280.9148)
tau (square root of estimated tau^2 value): 0
I^2 (residual heterogeneity / unaccounted variability): 0.00%
H^2 (unaccounted variability / sampling variability): 1.00
R^2 (amount of heterogeneity accounted for): 0.00%

Test for Residual Heterogeneity:
QE(df = 2) = 1.7588, p-val = 0.4150

Test of Moderators (coefficient 2):
F(df1 = 1, df2 = 2) = 0.5801, p-val = 0.5258

Model Results:

	estimate	se	tval	df	pval	ci.lb	ci.ub
intrcpt	25.5991	37.6863	0.6793	2	0.5670	-136.5517	187.7500
Epochs_seconds	-0.5183	0.6805	-0.7616	2	0.5258	-3.4464	2.4097

Signif. codes: 0 ‘***’ 0.001 ‘**’ 0.01 ‘*’ 0.05 ‘.’ 0.1 ‘ ’ 1

FU_mean

Mixed-Effects Model (k = 5; tau^2 estimator: REML)

tau^2 (estimated amount of residual heterogeneity): 0 (SE = 224.1889)
tau (square root of estimated tau^2 value): 0
I^2 (residual heterogeneity / unaccounted variability): 0.00%
H^2 (unaccounted variability / sampling variability): 1.00
R^2 (amount of heterogeneity accounted for): 0.00%

Test for Residual Heterogeneity:
QE(df = 3) = 2.4944, p-val = 0.4763

Test of Moderators (coefficient 2):
F(df1 = 1, df2 = 3) = 0.0265, p-val = 0.8811

Model Results:

	estimate	se	tval	df	pval	ci.lb	ci.ub
intrcpt	-3.3480	11.4555	-0.2923	3	0.7891	-39.8043	33.1084
Follow_up_actigraphy_days_mean	-0.1904	1.1704	-0.1627	3	0.8811	-3.9151	3.5344

intrcpt
Follow_up_actigraphy_days_mean

Signif. codes: 0 '***' 0.001 '**' 0.01 '*' 0.05 '.' 0.1 ' ' 1

FU_max

Mixed-Effects Model (k = 5; tau^2 estimator: REML)

tau^2 (estimated amount of residual heterogeneity): 0 (SE = 222.9958)
tau (square root of estimated tau^2 value): 0
I^2 (residual heterogeneity / unaccounted variability): 0.00%
H^2 (unaccounted variability / sampling variability): 1.00
R^2 (amount of heterogeneity accounted for): 0.00%

Test for Residual Heterogeneity:
QE(df = 3) = 2.4560, p-val = 0.4833

Test of Moderators (coefficient 2):
F(df1 = 1, df2 = 3) = 0.0738, p-val = 0.8036

Model Results:

	estimate	se	tval	df	pval	ci.lb	ci.ub
intrcpt	-2.0945	12.1225	-0.1728	3	0.8738	-40.6737	36.4848
Follow_up_actigraphy_days_max	-0.3307	1.2175	-0.2716	3	0.8036	-4.2054	3.5441

Signif. codes: 0 '***' 0.001 '**' 0.01 '*' 0.05 '.' 0.1 ' ' 1

RESULTS TIB

Forest plot – all studies

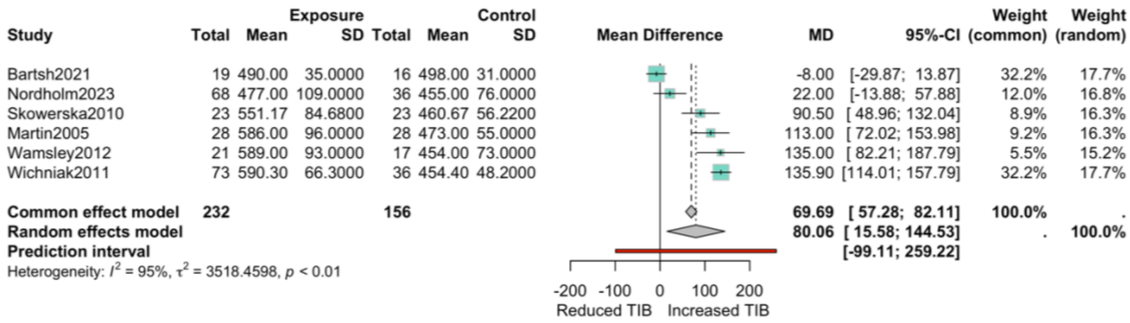

Figure 49

```
                  MD             95%-CI %W(common) %W(random)
Bartsh2021     -8.0000 [-29.8724;  13.8724]       32.2       17.7
Martin2005    113.0000 [ 72.0195; 153.9805]        9.2       16.3
Nordholm2023   22.0000 [-13.8821;  57.8821]       12.0       16.8
Skowerska2010  90.5000 [ 48.9603; 132.0397]        8.9       16.3
Wamsley2012   135.0000 [ 82.2144; 187.7856]        5.5       15.2
Wichniak2011  135.9000 [114.0089; 157.7911]       32.2       17.7

Number of studies: k = 6
Number of observations: o = 388 (o.e = 232, o.c = 156)

                          MD             95%-CI   z|t  p-value
Common effect model  69.6923 [ 57.2770;  82.1077] 11.00 < 0.0001
Random effects model 80.0555 [ 15.5804; 144.5306]  3.19   0.0242
Prediction interval          [-99.1071; 259.2181]

Quantifying heterogeneity:
 tau^2 = 3518.4598 [1183.8058; 21915.7246]; tau = 59.3166 [34.4065; 148.0396]
 I^2 = 95.1% [91.7%; 97.1%]; H = 4.51 [3.47; 5.86]

Test of heterogeneity:
      Q d.f.  p-value
 101.53    5 < 0.0001

Details on meta-analytical method:
- Inverse variance method
- Restricted maximum-likelihood estimator for tau^2
- Q-Profile method for confidence interval of tau^2 and tau
- Hartung-Knapp adjustment for random effects model (df = 5)
```

- Prediction interval based on t-distribution (df = 4)

Funnel plot

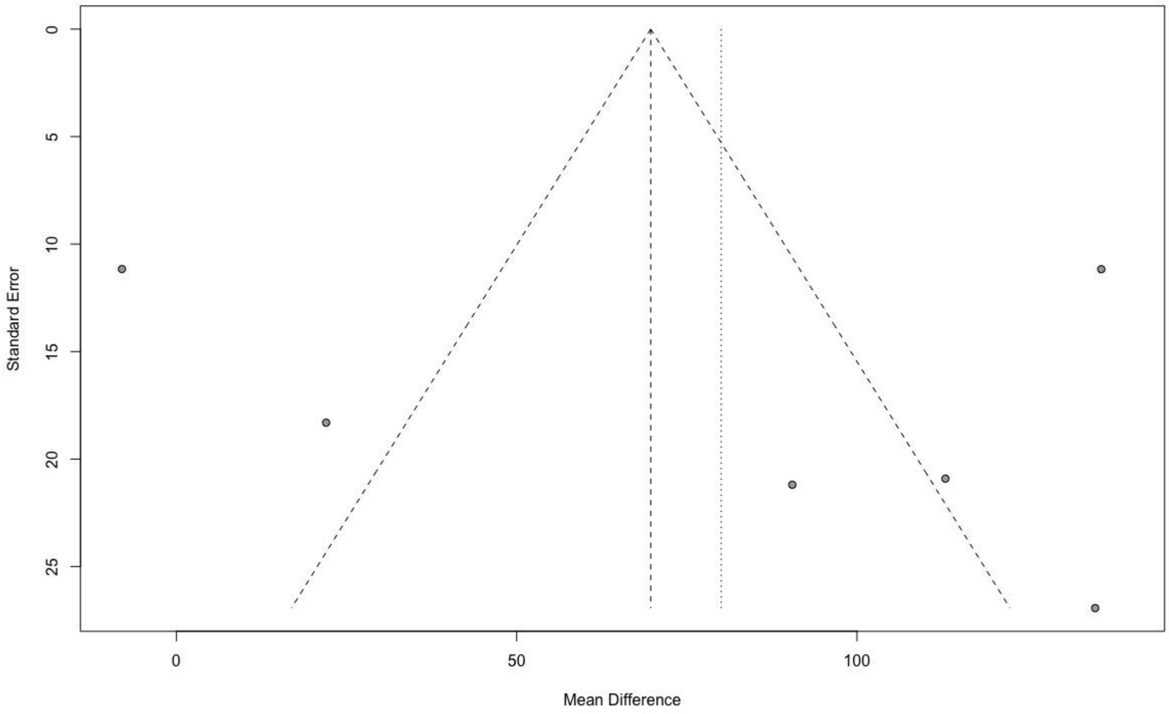

Figure 50

Eggers' test of the intercept

intercept	95% CI	t	p
2.929	-9.2 - 15.06	0.473	0.6606381

Eggers' test does not indicate the presence of funnel plot asymmetry.

Sensitivity analyses

SSD studies

Forest plot

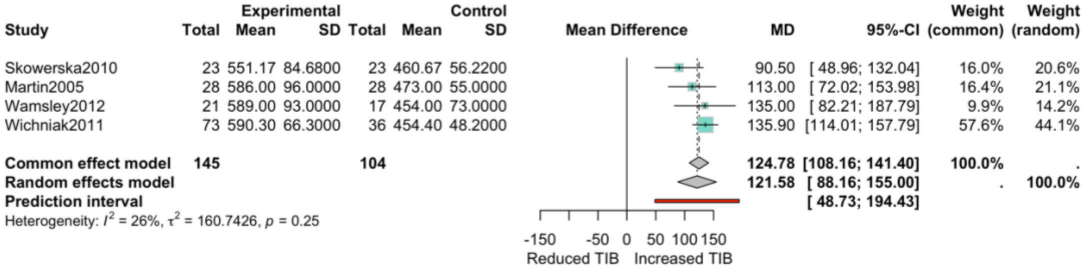

Figure 51

Number of studies: k = 4
Number of observations: o = 249 (o.e = 145, o.c = 104)

	MD	95%-CI	z|t	p-value
Common effect model	124.7777	[108.1585; 141.3969]	14.72	< 0.0001
Random effects model	121.5798	[88.1569; 155.0027]	11.58	0.0014
Prediction interval		[48.7346; 194.4250]		

Quantifying heterogeneity:
tau^2 = 160.7426 [0.0000; >1607.4259]; tau = 12.6784 [0.0000; >40.0927]
I^2 = 26.3% [0.0%; 72.0%]; H = 1.16 [1.00; 1.89]

Test of heterogeneity:

Q	d.f.	p-value
4.07	3	0.2541

Details on meta-analytical method:
- Inverse variance method
- Restricted maximum-likelihood estimator for tau^2
- Q-Profile method for confidence interval of tau^2 and tau
- Hartung-Knapp adjustment for random effects model (df = 3)
- Prediction interval based on t-distribution (df = 2)

CHR-P studies

Forest plot

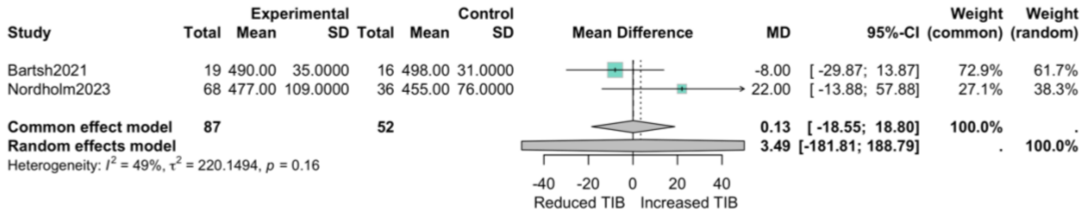

Figure 52

Number of studies: k = 2
Number of observations: o = 139 (o.e = 87, o.c = 52)

	MD	95%-CI	z|t	p-value
Common effect model	0.1272	[-18.5489; 18.8034]	0.01	0.9893
Random effects model	3.4895	[-181.8106; 188.7896]	0.24	0.8505
Prediction interval				

Quantifying heterogeneity:
tau^2 = 220.1494; tau = 14.8374; I^2 = 48.9%; H = 1.40

Test of heterogeneity:

Q	d.f.	p-value
1.96	1	0.1617

Details on meta-analytical method:
- Inverse variance method
- Restricted maximum-likelihood estimator for tau^2
- Hartung-Knapp adjustment for random effects model (df = 1)

Outliers and heterogeneity

Identified outliers (fixed-effect model)

"Bartsh2021", "Wamsley2012", "Wichniak2011"

Results with outliers removed

Review: TIB

Number of studies: k = 3
Number of observations: o = 206 (o.e = 119, o.c = 87)

	MD	95%-CI	z|t	p-value

Common effect model 70.1044 [47.4685; 92.7403] 6.07 < 0.0001
Random effects model 74.3449 [-44.2345; 192.9243] 2.70 0.1143
Prediction interval [-582.9811; 731.6708]

Quantifying heterogeneity:
tau^2 = 1905.6948 [235.6562; >19056.9482]; tau = 43.6543 [15.3511; >138.0469]
I^2 = 83.4% [49.7%; 94.5%]; H = 2.45 [1.41; 4.27]

Test of heterogeneity:
Q d.f. p-value
12.04 2 0.0024

Details on meta-analytical method:
- Inverse variance method
- Restricted maximum-likelihood estimator for tau^2
- Q-Profile method for confidence interval of tau^2 and tau
- Hartung-Knapp adjustment for random effects model (df = 2)
- Prediction interval based on t-distribution (df = 1)

Identified outliers (random-effects model)

"Bartsh2021"

Results with outliers removed

Review: TIB

Number of studies: k = 5
Number of observations: o = 353 (o.e = 213, o.c = 140)

MD 95%-CI z|t p-value
Common effect model 106.6243 [91.5440; 121.7045] 13.86 < 0.0001
Random effects model 98.9366 [39.9169; 157.9562] 4.65 0.0096
Prediction interval [-56.5547; 254.4278]

Quantifying heterogeneity:
tau^2 = 1922.9652 [462.1623; 17917.3486]; tau = 43.8516 [21.4980; 133.8557]
I^2 = 86.7% [71.1%; 93.9%]; H = 2.74 [1.86; 4.03]

Test of heterogeneity:
Q d.f. p-value
30.02 4 < 0.0001

Details on meta-analytical method:
- Inverse variance method
- Restricted maximum-likelihood estimator for tau^2
- Q-Profile method for confidence interval of tau^2 and tau

- Hartung-Knapp adjustment for random effects model (df = 4)
- Prediction interval based on t-distribution (df = 3)

Influence analyses

Baujat plot

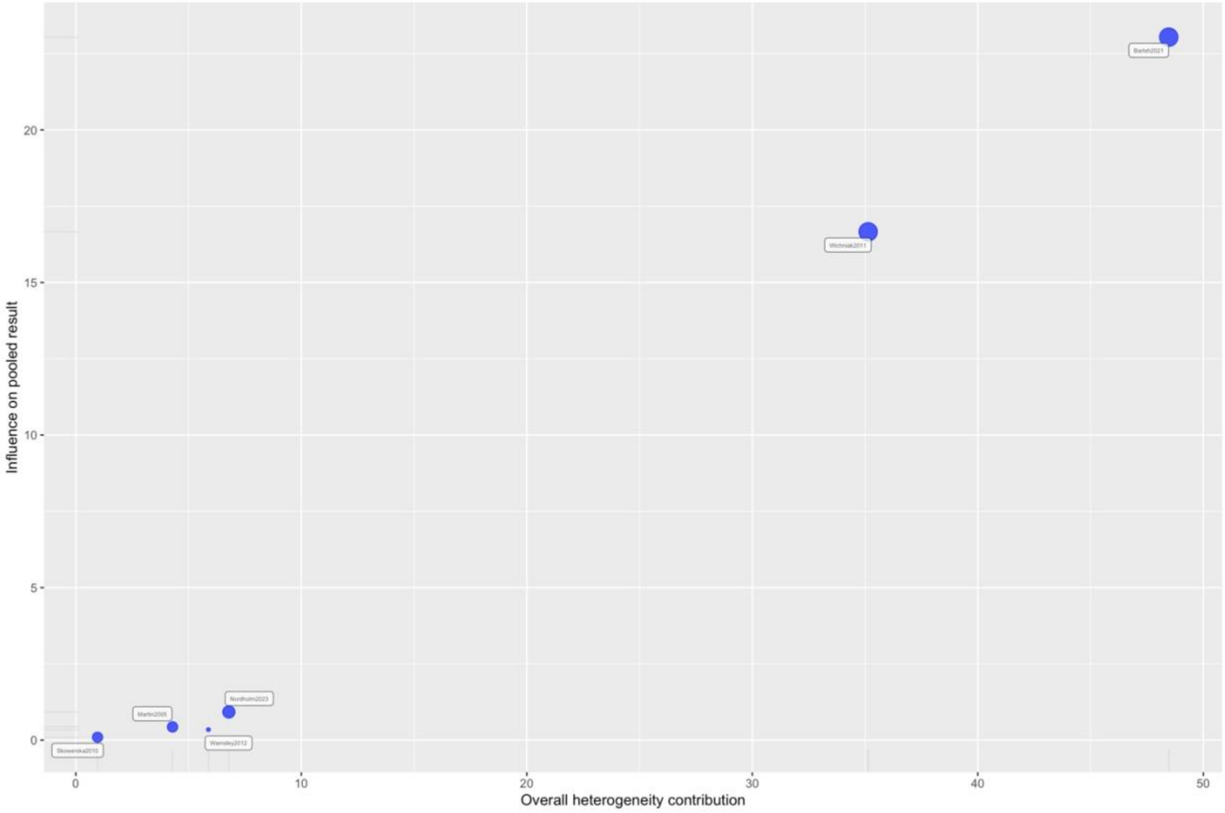

Figure 53

Influence diagnostics

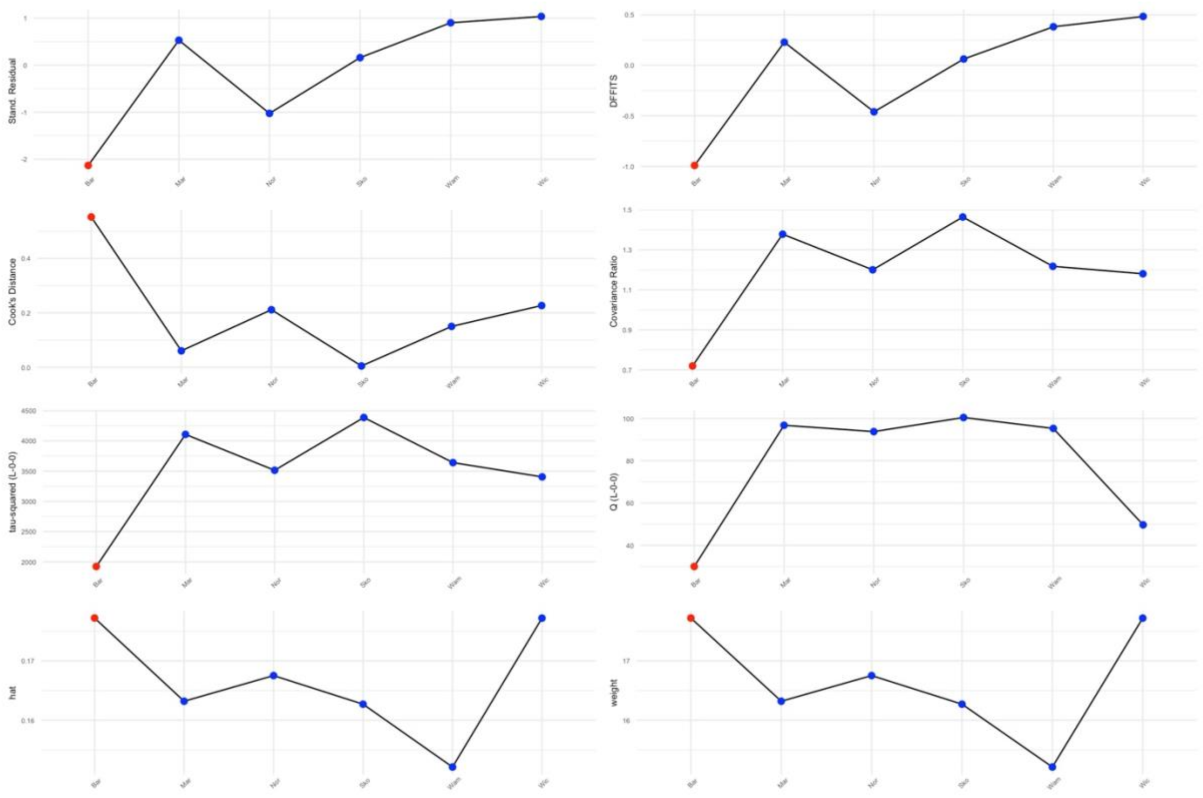

Figure 54

RESULTS SLEEP LATENCY

Forest plot – all studies

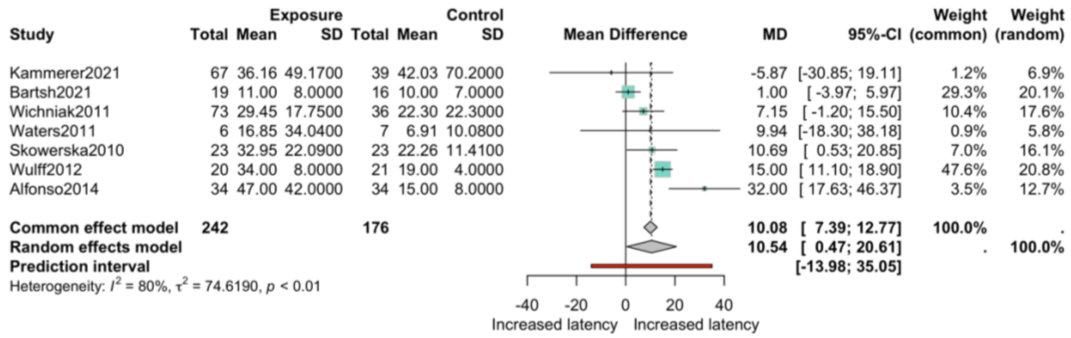

Figure 55

	MD	95%-CI	%W(common)	%W(random)
Alfonso2014	32.0000	[17.6287; 46.3713]	3.5	12.7
Bartsh2021	1.0000	[-3.9703; 5.9703]	29.3	20.1
Kammerer2021	-5.8700	[-30.8505; 19.1105]	1.2	6.9
Skowerska2010	10.6900	[0.5291; 20.8509]	7.0	16.1

Waters2011 9.9400 [-18.3022; 38.1822] 0.9 5.8
Wichniak2011 7.1500 [-1.1953; 15.4953] 10.4 17.6
Wulff2012 15.0000 [11.0988; 18.9012] 47.6 20.8

Number of studies: k = 7
Number of observations: o = 418 (o.e = 242, o.c = 176)

```
                    MD            95%-CI  z|t  p-value
Common effect model  10.0797 [  7.3870; 12.7723] 7.34 < 0.0001
Random effects model 10.5365 [  0.4661; 20.6069] 2.56   0.0429
Prediction interval         [-13.9780; 35.0510]
```

Quantifying heterogeneity:
tau^2 = 74.6190 [13.3588; 611.6696]; tau = 8.6382 [3.6550; 24.7320]
I^2 = 79.9% [59.1%; 90.2%]; H = 2.23 [1.56; 3.19]

Test of heterogeneity:

```
    Q d.f.  p-value
 29.92    6 < 0.0001
```

Details on meta-analytical method:
- Inverse variance method
- Restricted maximum-likelihood estimator for tau^2
- Q-Profile method for confidence interval of tau^2 and tau
- Hartung-Knapp adjustment for random effects model (df = 6)
- Prediction interval based on t-distribution (df = 5)

Funnel plot

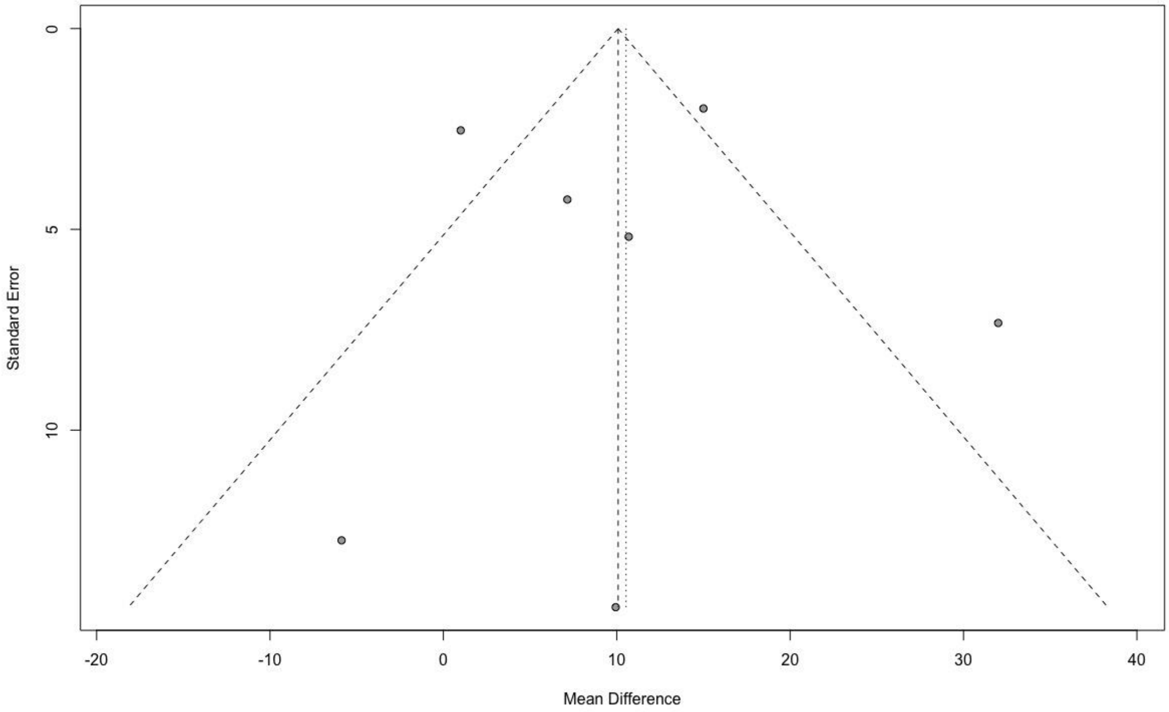

Figure 56

Eggers' test of the intercept

intercept 95% CI t p
0.023 -3.27 - 3.32 0.014 0.9894104

Eggers' test does not indicate the presence of funnel plot asymmetry.

SSD studies

Forest plot

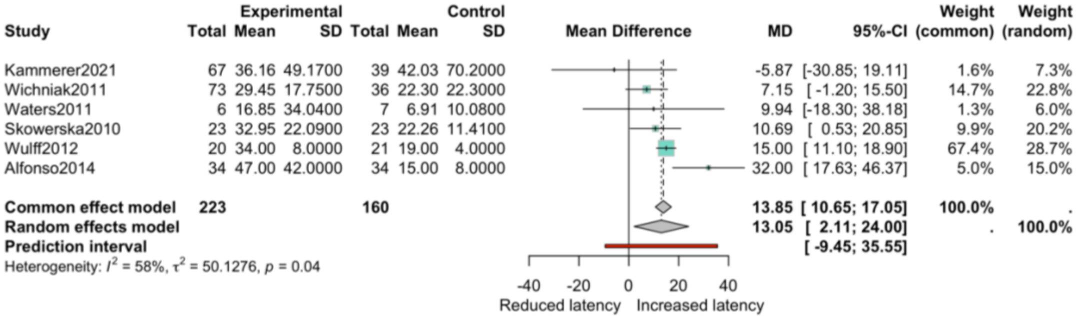

Figure 57

Number of studies: k = 6
Number of observations: o = 383 (o.e = 223, o.c = 160)

	MD	95%-CI	z|t	p-value
Common effect model	13.8513	[10.6479; 17.0547]	8.47	< 0.0001
Random effects model	13.0546	[2.1104; 23.9988]	3.07	0.0279
Prediction interval		[-9.4451; 35.5543]		

Quantifying heterogeneity:
tau^2 = 50.1276 [0.0000; >501.2757]; tau = 7.0801 [0.0000; >22.3892]
I^2 = 57.5% [0.0%; 82.8%]; H = 1.53 [1.00; 2.41]

Test of heterogeneity:

Q	d.f.	p-value
11.78	5	0.0380

Details on meta-analytical method:
- Inverse variance method
- Restricted maximum-likelihood estimator for tau^2
- Q-Profile method for confidence interval of tau^2 and tau
- Hartung-Knapp adjustment for random effects model (df = 5)
- Prediction interval based on t-distribution (df = 4)

CHR-P studies

Forest plot

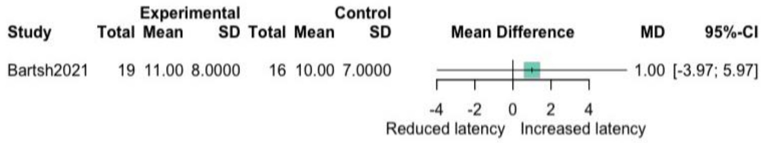

Figure 58

Review: CHR-P_latency

Number of observations: o = 35 (o.e = 19, o.c = 16)

```
            MD          95%-CI    z p-value
Bartsh2021 1.0000 [-3.9703; 5.9703] 0.39  0.6933
```

Outliers and heterogeneity

Identified outliers (fixed-effect model)

"Alfonso2014", "Bartsh2021"

Results with outliers removed

Review: latency

Number of studies: k = 5

Number of observations: o = 315 (o.e = 189, o.c = 126)

```
                        MD          95%-CI  z|t  p-value
Common effect model  12.9024 [ 9.6163; 16.1885] 7.70 < 0.0001
Random effects model 11.1344 [ 4.3918; 17.8770] 4.58   0.0101
Prediction interval         [-2.7420; 25.0108]
```

Quantifying heterogeneity:

tau^2 = 11.5701 [0.0000; >115.7013]; tau = 3.4015 [0.0000; >10.7565]

I^2 = 24.9% [0.0%; 69.7%]; H = 1.15 [1.00; 1.82]

Test of heterogeneity:

```
    Q d.f. p-value
 5.33    4  0.2551
```

Details on meta-analytical method:

- Inverse variance method
- Restricted maximum-likelihood estimator for tau^2
- Q-Profile method for confidence interval of tau^2 and tau

- Hartung-Knapp adjustment for random effects model (df = 4)
- Prediction interval based on t-distribution (df = 3)

Identified outliers (random-effects model)

Results with outliers removed

Review: latency

Number of studies: k = 7
Number of observations: o = 418 (o.e = 242, o.c = 176)

```
                     MD            95%-CI  z|t  p-value
Common effect model  10.0797 [  7.3870; 12.7723] 7.34 < 0.0001
Random effects model 10.5365 [  0.4661; 20.6069] 2.56   0.0429
Prediction interval         [-13.9780; 35.0510]
```

Quantifying heterogeneity:
tau^2 = 74.6190 [13.3588; 611.6696]; tau = 8.6382 [3.6550; 24.7320]
I^2 = 79.9% [59.1%; 90.2%]; H = 2.23 [1.56; 3.19]

Test of heterogeneity:

```
    Q d.f.  p-value
 29.92    6 < 0.0001
```

Details on meta-analytical method:
- Inverse variance method
- Restricted maximum-likelihood estimator for tau^2
- Q-Profile method for confidence interval of tau^2 and tau
- Hartung-Knapp adjustment for random effects model (df = 6)
- Prediction interval based on t-distribution (df = 5)

Influence analyses

Baujat plot

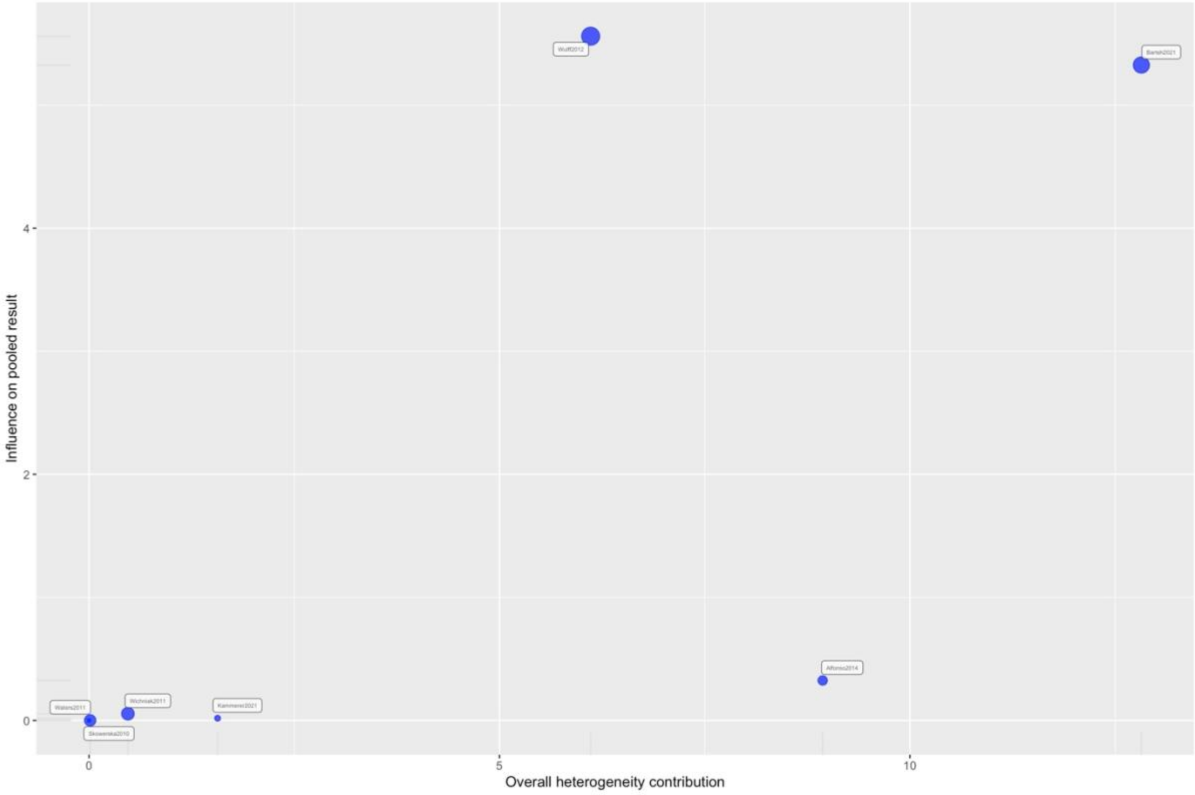

Figure 59

Influence diagnostics

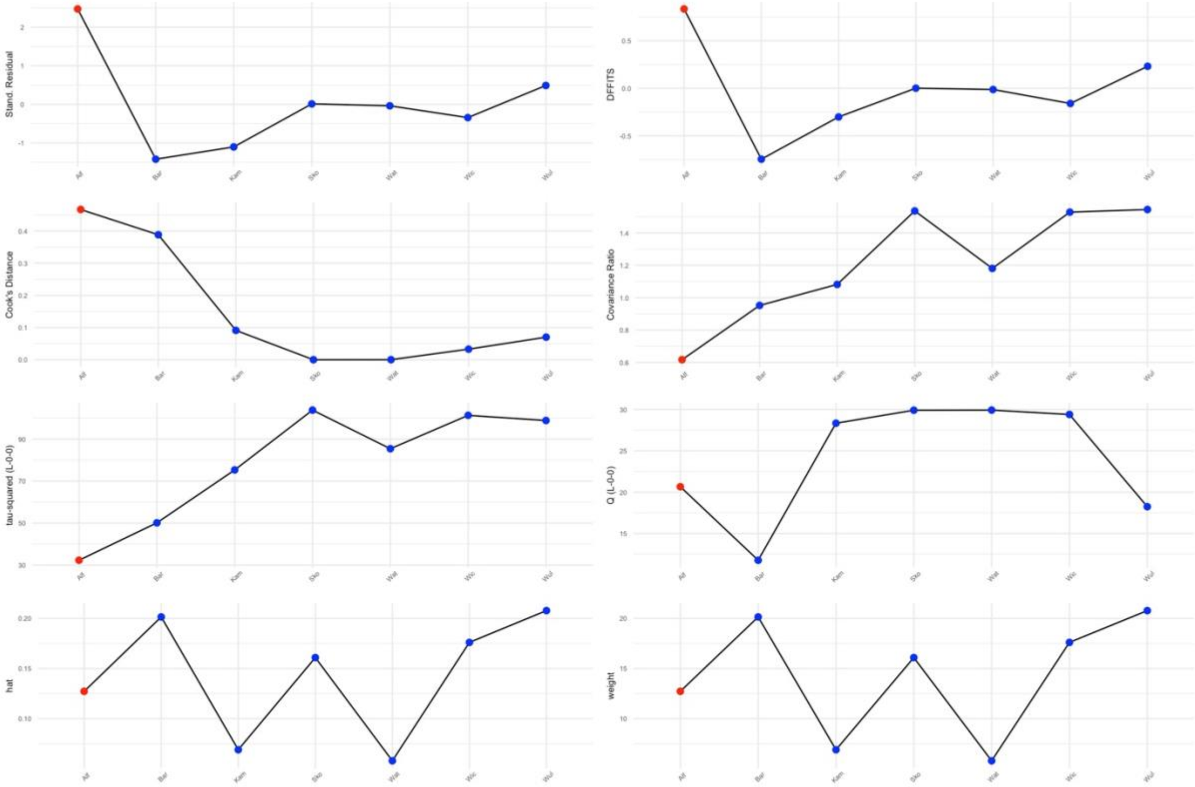

Figure 60

RESULTS SLEEP EFFICIENCY

Forest plot – all studies

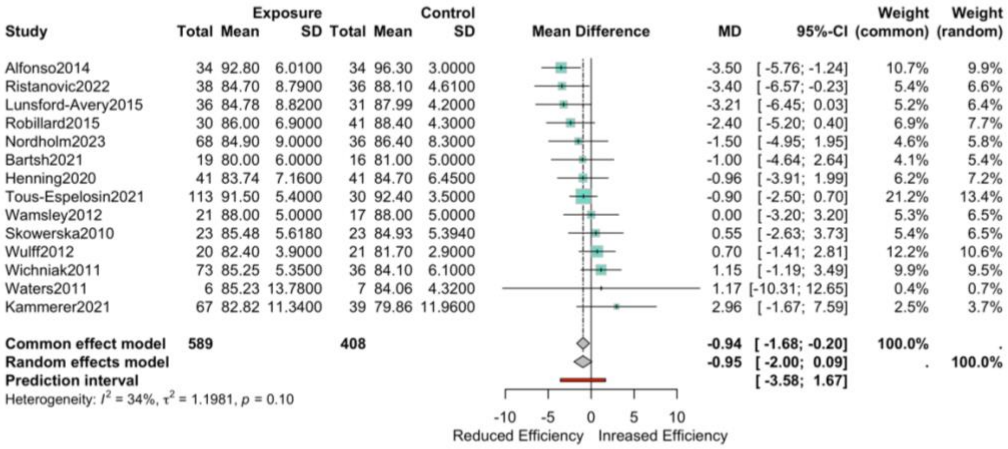

Figure 61

```
                    MD            95%-CI %W(common) %W(random)
Alfonso2014        -3.5000 [ -5.7578; -1.2422]       10.7        9.9
Bartsh2021        -1.0000 [ -4.6443;  2.6443]        4.1        5.4
Henning2020        -0.9600 [ -3.9098;  1.9898]        6.2        7.2
Kammerer2021        2.9600 [ -1.6728;  7.5928]        2.5        3.7
Lunsford-Avery2015 -3.2100 [ -6.4484;  0.0284]        5.2        6.4
Nordholm2023       -1.5000 [ -4.9535;  1.9535]        4.6        5.8
Ristanovic2022     -3.4000 [ -6.5747; -0.2253]        5.4        6.6
Robillard2015      -2.4000 [ -5.1980;  0.3980]        6.9        7.7
Skowerska2010       0.5500 [ -2.6329;  3.7329]        5.4        6.5
Tous-Espelosin2021 -0.9000 [ -2.5000;  0.7000]       21.2       13.4
Wamsley2012         0.0000 [ -3.1972;  3.1972]        5.3        6.5
Waters2011          1.1700 [-10.3111; 12.6511]        0.4        0.7
Wichniak2011        1.1500 [ -1.1902;  3.4902]        9.9        9.5
Wulff2012           0.7000 [ -1.4118;  2.8118]       12.2       10.6

Number of studies: k = 14
Number of observations: o = 997 (o.e = 589, o.c = 408)

                          MD            95%-CI  z|t p-value
Common effect model  -0.9407 [-1.6778; -0.2036] -2.50  0.0124
Random effects model -0.9540 [-2.0013;  0.0933] -1.97  0.0708
Prediction interval          [-3.5762;  1.6682]

Quantifying heterogeneity:
 tau^2 = 1.1981 [0.0000; 6.3904]; tau = 1.0946 [0.0000; 2.5279]
```

I^2 = 34.0% [0.0%; 65.1%]; H = 1.23 [1.00; 1.69]

Test of heterogeneity:
Q d.f. p-value
19.69 13 0.1032

Details on meta-analytical method:
- Inverse variance method
- Restricted maximum-likelihood estimator for tau^2
- Q-Profile method for confidence interval of tau^2 and tau
- Hartung-Knapp adjustment for random effects model (df = 13)
- Prediction interval based on t-distribution (df = 12)

Funnel plot

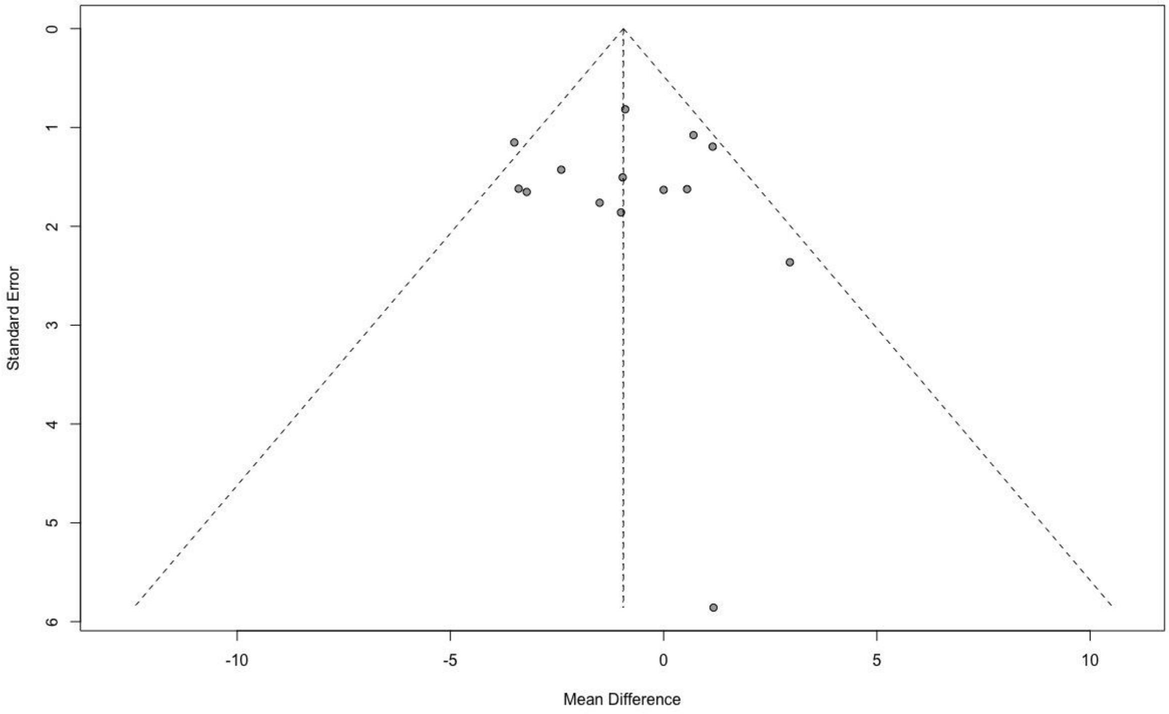

Figure 62

Linear regression test of funnel plot asymmetry

Test result: t = 0.21, df = 12, p-value = 0.8400
Bias estimate: 0.2104 (SE = 1.0196)

Details:
- multiplicative residual heterogeneity variance (tau^2 = 1.6350)
- predictor: standard error

- weight: inverse variance
- reference: Egger et al. (1997), BMJ

Eggers' test of the intercept

intercept   95% CI   t   p
0.21 -1.79 - 2.21 0.206 0.8400112

Eggers' test does not indicate the presence of funnel plot asymmetry.

SSD studies

Forest plot

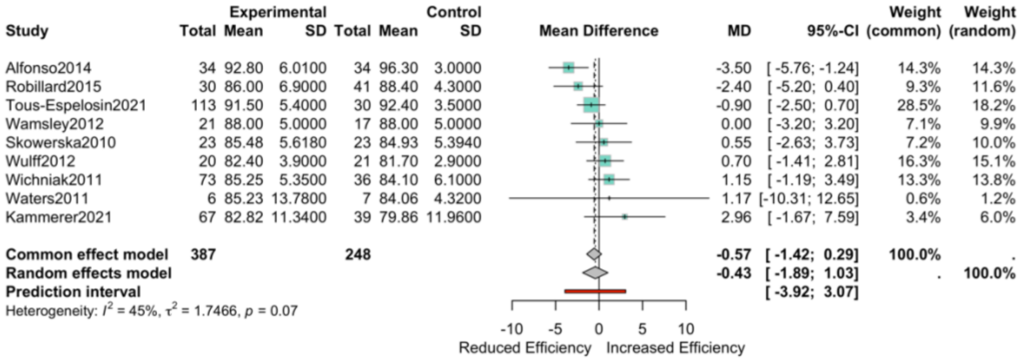

Figure 63

Number of studies: k = 9
Number of observations: o = 635 (o.e = 387, o.c = 248)

MD   95%-CI   z|t p-value
Common effect model -0.5661 [-1.4198; 0.2876] -1.30 0.1937
Random effects model -0.4299 [-1.8888; 1.0291] -0.68 0.5160
Prediction interval   [-3.9250; 3.0653]

Quantifying heterogeneity:
tau^2 = 1.7466 [0.0000; 11.1061]; tau = 1.3216 [0.0000; 3.3326]
I^2 = 45.4% [0.0%; 74.7%]; H = 1.35 [1.00; 1.99]

Test of heterogeneity:
Q d.f. p-value
14.66   8 0.0662

Details on meta-analytical method:

- Inverse variance method
- Restricted maximum-likelihood estimator for tau^2
- Q-Profile method for confidence interval of tau^2 and tau
- Hartung-Knapp adjustment for random effects model (df = 8)
- Prediction interval based on t-distribution (df = 7)

CHR-P studies

Forest plot

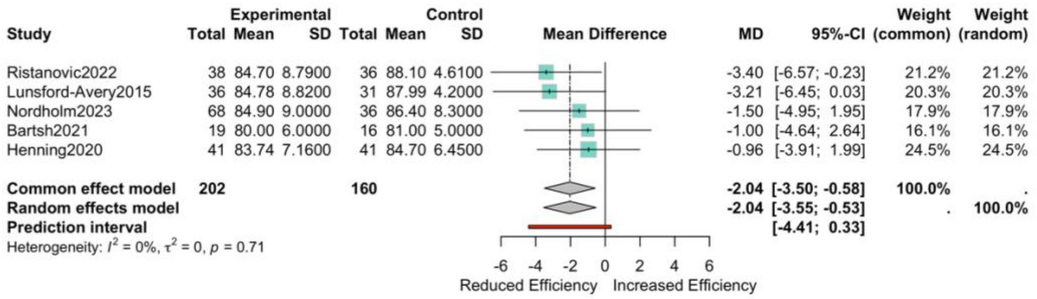

Figure 64

Review: CHR_Efficiency

Number of studies: k = 5
Number of observations: o = 362 (o.e = 202, o.c = 160)

	MD	95%-CI	z|t	p-value
Common effect model	-2.0374	[-3.4982; -0.5767]	-2.73	0.0063
Random effects model	-2.0374	[-3.5468; -0.5281]	-3.75	0.0200
Prediction interval		[-4.4093; 0.3344]		

Quantifying heterogeneity:
tau^2 = 0 [0.0000; 9.1390]; tau = 0 [0.0000; 3.0231]
I^2 = 0.0% [0.0%; 79.2%]; H = 1.00 [1.00; 2.19]

Test of heterogeneity:

Q	d.f.	p-value
2.13	4	0.7122

Details on meta-analytical method:
- Inverse variance method
- Restricted maximum-likelihood estimator for tau^2
- Q-Profile method for confidence interval of tau^2 and tau
- Hartung-Knapp adjustment for random effects model (df = 4)
- Prediction interval based on t-distribution (df = 3)

Outliers and heterogeneity

No outliers detected (fixed-effect/random-effects model)

Influence analyses

Baujat plot

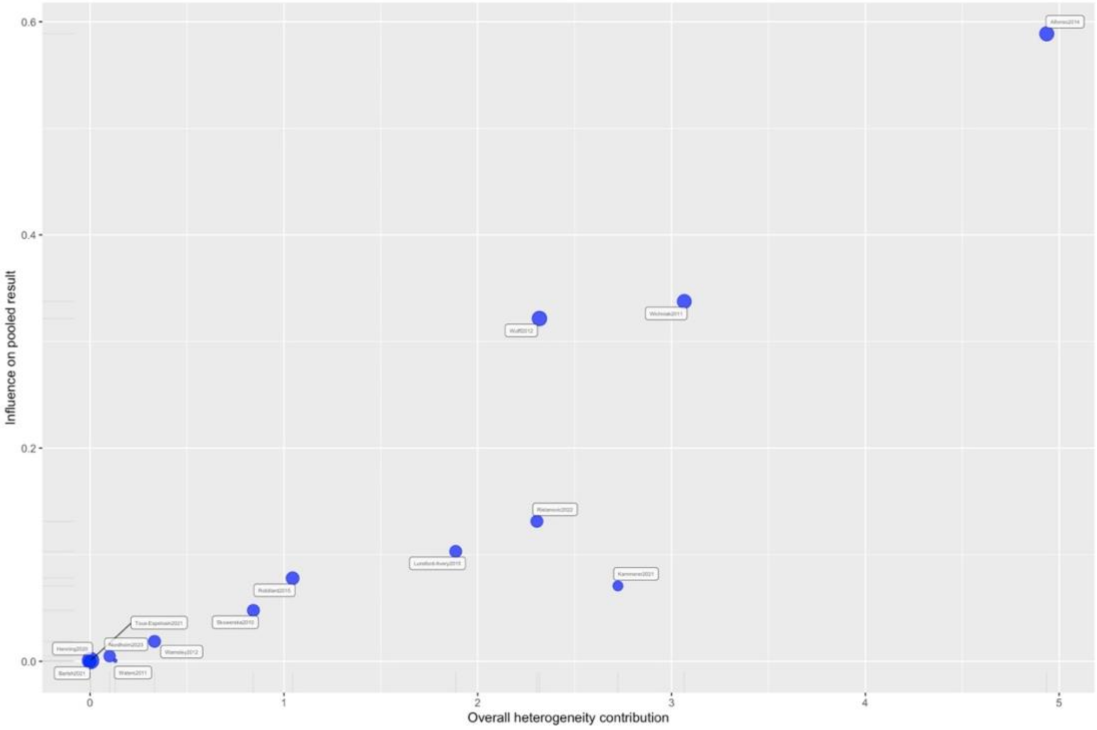

Figure 65

Influence diagnostics

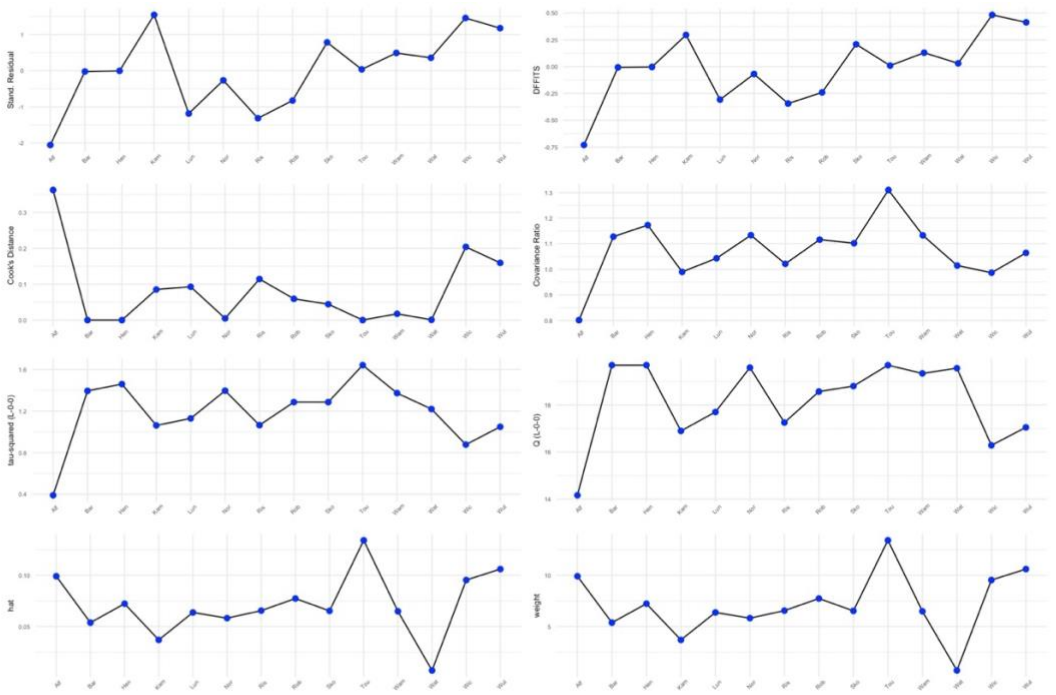

Figure 66

RESULTS WASO

Forest plot – all studies

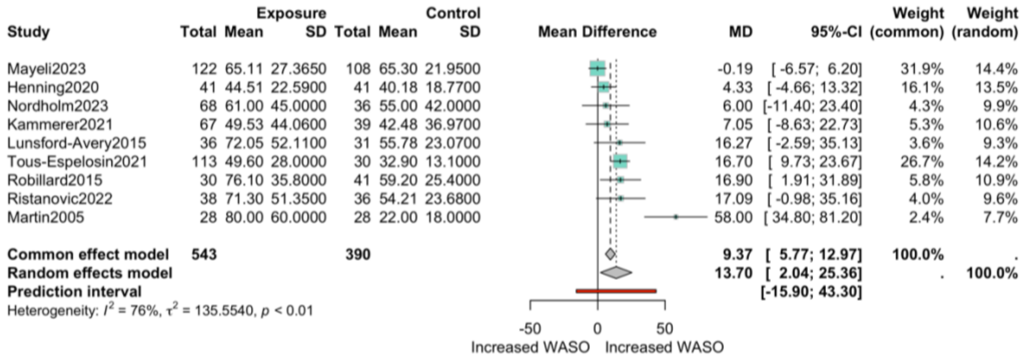

Figure 67

	MD	95%-CI	%W(common)	%W(random)
Henning2020	4.3300	[-4.6601; 13.3201]	16.1	13.5
Kammerer2021	7.0500	[-8.6322; 22.7322]	5.3	10.6
Lunsford-Avery2015	16.2700	[-2.5903; 35.1303]	3.6	9.3
Martin2005	58.0000	[34.7976; 81.2024]	2.4	7.7
Mayeli2023	-0.1850	[-6.5659; 6.1959]	31.9	14.4
Nordholm2023	6.0000	[-11.3962; 23.3962]	4.3	9.9

Ristanovic2022 17.0900 [-0.9764; 35.1564] 4.0 9.6
Robillard2015 16.9000 [1.9147; 31.8853] 5.8 10.9
Tous-Espelosin2021 16.7000 [9.7267; 23.6733] 26.7 14.2

Number of studies: k = 9
Number of observations: o = 933 (o.e = 543, o.c = 390)

MD 95%-CI z|t p-value
Common effect model 9.3707 [5.7682; 12.9732] 5.10 < 0.0001
Random effects model 13.6994 [2.0358; 25.3630] 2.71 0.0267
Prediction interval [-15.8984; 43.2972]

Quantifying heterogeneity:
tau^2 = 135.5540 [37.8705; 962.7086]; tau = 11.6428 [6.1539; 31.0275]
I^2 = 76.0% [54.0%; 87.5%]; H = 2.04 [1.47; 2.83]

Test of heterogeneity:
Q d.f. p-value
33.35 8 < 0.0001

Details on meta-analytical method:
- Inverse variance method
- Restricted maximum-likelihood estimator for tau^2
- Q-Profile method for confidence interval of tau^2 and tau
- Hartung-Knapp adjustment for random effects model (df = 8)
- Prediction interval based on t-distribution (df = 7)

Funnel plot

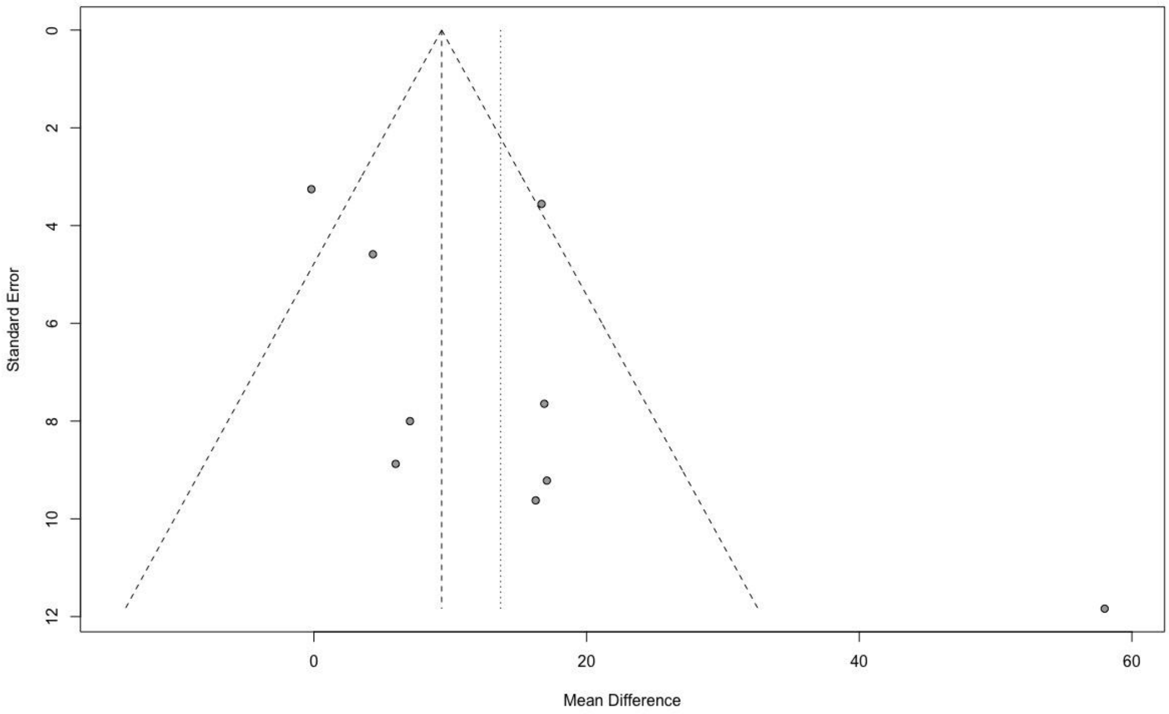

Figure 68

Eggers' test of the intercept

intercept 95% CI t p
2.377 -0.41 - 5.16 1.672 0.138535

Eggers' test does not indicate the presence of funnel plot asymmetry.

Sensitivity analyses

SSD studies

Forest plot

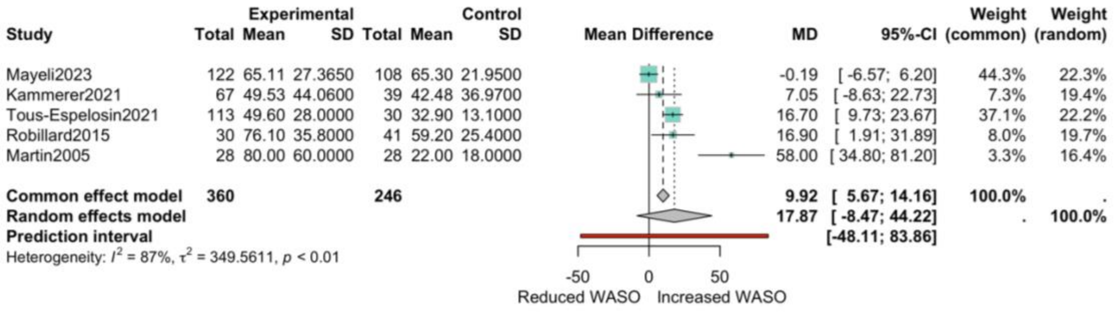

Figure 69

Number of studies: k = 5
Number of observations: o = 606 (o.e = 360, o.c = 246)

```
                      MD            95%-CI  z|t  p-value
Common effect model   9.9195 [  5.6748; 14.1642] 4.58 < 0.0001
Random effects model 17.8738 [ -8.4702; 44.2179] 1.88   0.1327
Prediction interval          [-48.1107; 83.8584]
```

Quantifying heterogeneity:
tau^2 = 349.5611 [84.3590; >3495.6111]; tau = 18.6966 [9.1847; >59.1237]
I^2 = 87.0% [71.9%; 94.0%]; H = 2.77 [1.89; 4.07]

Test of heterogeneity:

```
    Q d.f.  p-value
30.72    4 < 0.0001
```

Details on meta-analytical method:
- Inverse variance method
- Restricted maximum-likelihood estimator for tau^2
- Q-Profile method for confidence interval of tau^2 and tau
- Hartung-Knapp adjustment for random effects model (df = 4)
- Prediction interval based on t-distribution (df = 3)

CHR-P studies

Forest plot

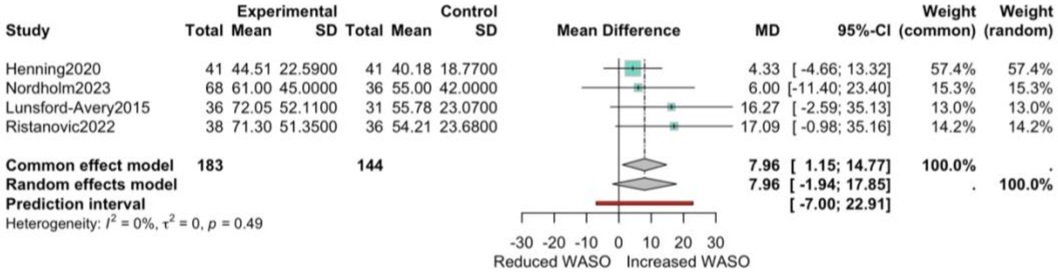

Figure 70

Number of studies: k = 4
Number of observations: o = 327 (o.e = 183, o.c = 144)

```
                     MD            95%-CI  z|t p-value
Common effect model  7.9574 [ 1.1457; 14.7691] 2.29  0.0220
Random effects model 7.9574 [-1.9391; 17.8539] 2.56  0.0833
Prediction interval         [-6.9961; 22.9109]
```

Quantifying heterogeneity:

tau^2 = 0 [0.0000; >100.0000]; tau = 0 [0.0000; >10.0000]
I^2 = 0.0% [0.0%; 84.7%]; H = 1.00 [1.00; 2.56]

Test of heterogeneity:
   Q d.f. p-value
2.40   3 0.4933

Details on meta-analytical method:
- Inverse variance method
- Restricted maximum-likelihood estimator for tau^2
- Q-Profile method for confidence interval of tau^2 and tau
- Hartung-Knapp adjustment for random effects model (df = 3)
- Prediction interval based on t-distribution (df = 2)

Outliers and heterogeneity

Identified outliers (fixed-effect model)

"Martin2005"

Results with outliers removed

Review: WASO

Number of studies: k = 8
Number of observations: o = 877 (o.e = 515, o.c = 362)

	MD	95%-CI	z|t	p-value
Common effect model	8.1694	[4.5227; 11.8162]	4.39	< 0.0001
Random effects model	9.3707	[3.1173; 15.6241]	3.54	0.0094
Prediction interval		[-6.9065; 25.6479]		

Quantifying heterogeneity:
tau^2 = 34.8805 [0.0438; 164.5819]; tau = 5.9060 [0.2093; 12.8289]
I^2 = 56.4% [4.0%; 80.2%]; H = 1.51 [1.02; 2.25]

Test of heterogeneity:
    Q d.f. p-value
16.06   7 0.0245

Details on meta-analytical method:
- Inverse variance method
- Restricted maximum-likelihood estimator for tau^2
- Q-Profile method for confidence interval of tau^2 and tau
- Hartung-Knapp adjustment for random effects model (df = 7)
- Prediction interval based on t-distribution (df = 6)

```
Identified outliers (random-effects model)
------------------------------------------
"Martin2005"

Results with outliers removed
-----------------------------
Review:     WASO

Number of studies: k = 8
Number of observations: o = 877 (o.e = 515, o.c = 362)

                      MD            95%-CI  z|t  p-value
Common effect model  8.1694 [ 4.5227; 11.8162] 4.39 < 0.0001
Random effects model 9.3707 [ 3.1173; 15.6241] 3.54   0.0094
Prediction interval         [-6.9065; 25.6479]

Quantifying heterogeneity:
 tau^2 = 34.8805 [0.0438; 164.5819]; tau = 5.9060 [0.2093; 12.8289]
 I^2 = 56.4% [4.0%; 80.2%]; H = 1.51 [1.02; 2.25]

Test of heterogeneity:
     Q d.f. p-value
 16.06    7  0.0245

Details on meta-analytical method:
- Inverse variance method
- Restricted maximum-likelihood estimator for tau^2
- Q-Profile method for confidence interval of tau^2 and tau
- Hartung-Knapp adjustment for random effects model (df = 7)
- Prediction interval based on t-distribution (df = 6)
```

Influence analyses

Baujat plot

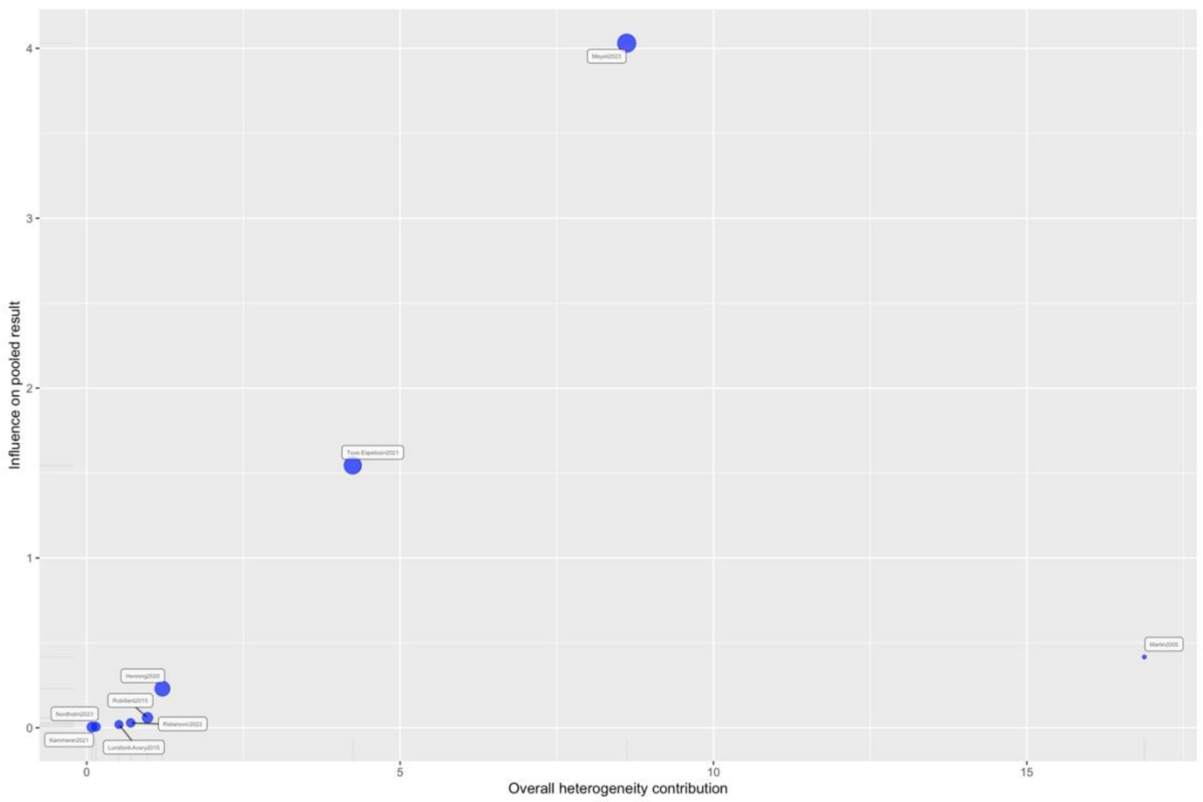

Figure 71

Influence diagnostics

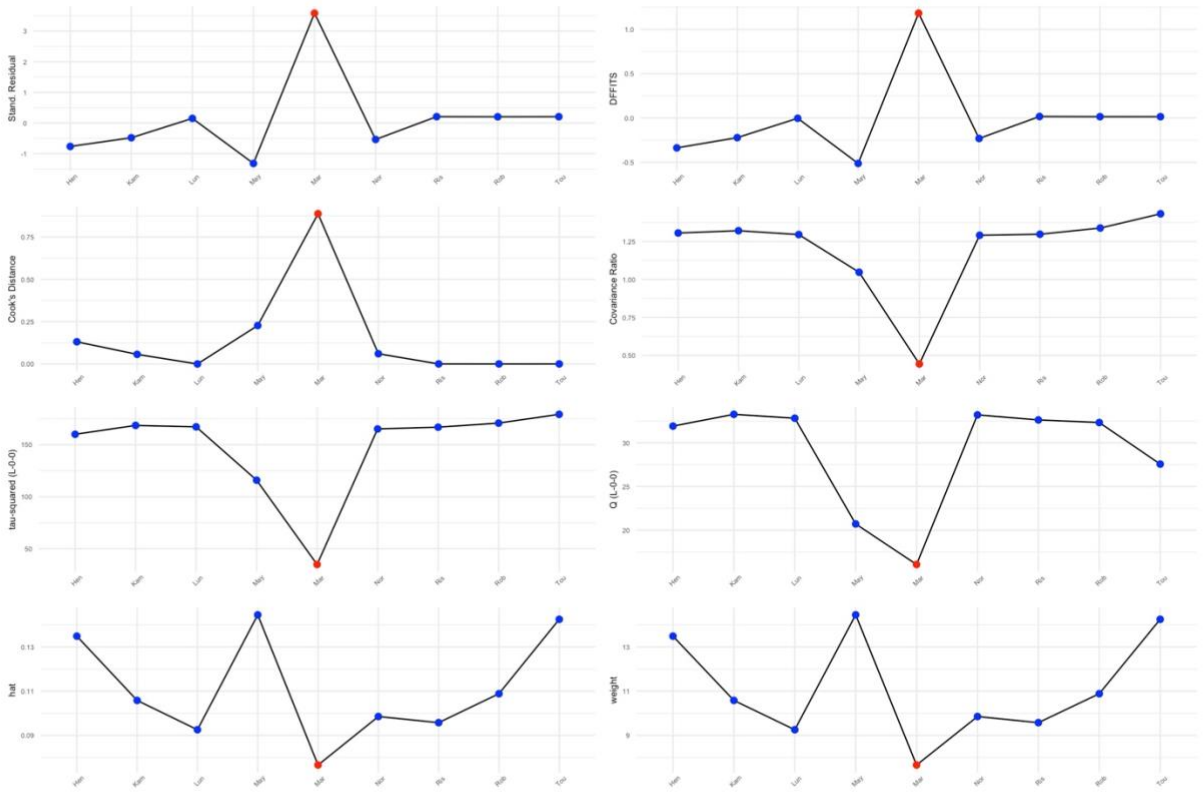

Figure 72

RESULTS Nighttime Awakenings

Forest plot – all studies

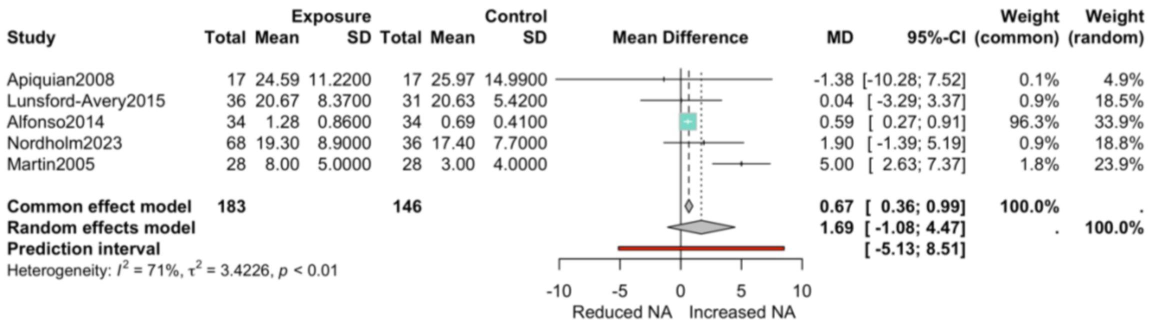

Figure 73

```
                    MD          95%-CI %W(common) %W(random)
Alfonso2014        0.5900 [  0.2698; 0.9102]      96.3      33.9
Apiquian2008      -1.3800 [-10.2807; 7.5207]       0.1       4.9
Lunsford-Avery2015  0.0400 [ -3.2940; 3.3740]      0.9      18.5
Martin2005         5.0000 [  2.6283; 7.3717]       1.8      23.9
Nordholm2023       1.9000 [ -1.3865; 5.1865]       0.9      18.8

Number of studies: k = 5
Number of observations: o = 329 (o.e = 183, o.c = 146)

                      MD          95%-CI  z|t  p-value
Common effect model  0.6721 [ 0.3578; 0.9864] 4.19 < 0.0001
Random effects model 1.6937 [-1.0841; 4.4716] 1.69   0.1657
Prediction interval         [-5.1262; 8.5137]

Quantifying heterogeneity:
 tau^2 = 3.4226 [0.2012; 41.9570]; tau = 1.8500 [0.4485; 6.4774]
 I^2 = 71.3% [27.3%; 88.7%]; H = 1.87 [1.17; 2.97]

Test of heterogeneity:
     Q d.f. p-value
 13.92    4  0.0075
```

Details on meta-analytical method:
- Inverse variance method
- Restricted maximum-likelihood estimator for tau^2
- Q-Profile method for confidence interval of tau^2 and tau
- Hartung-Knapp adjustment for random effects model (df = 4)
- Prediction interval based on t-distribution (df = 3)

Funnel plot

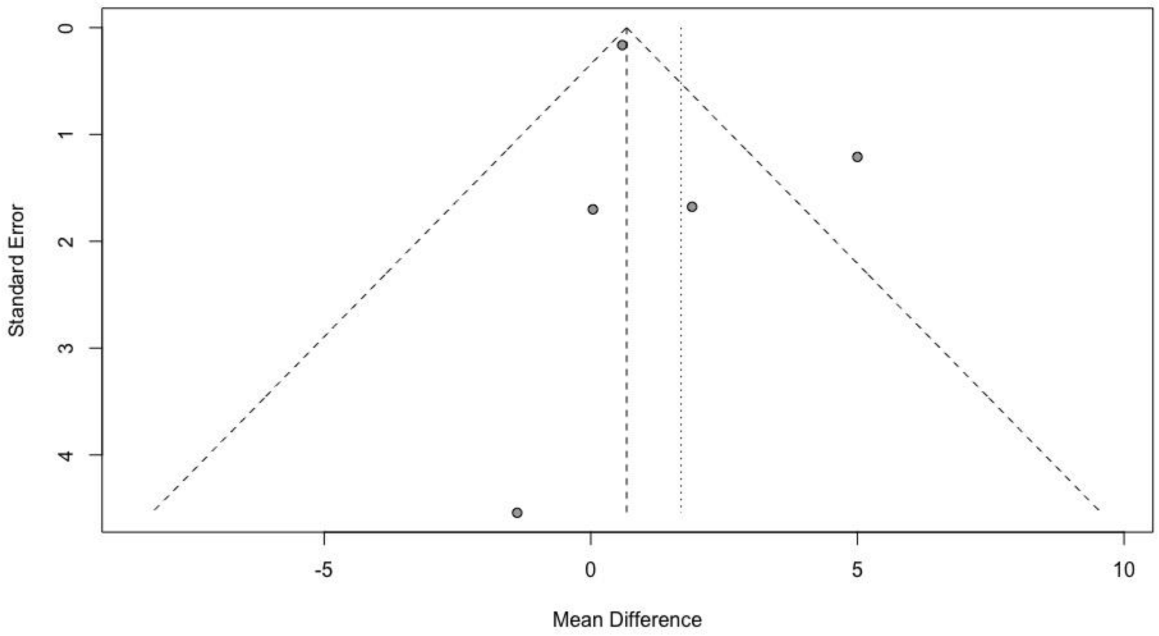

Figure 74

Eggers' test of the intercept

intercept	95% CI	t	p
0.93	-1.18 - 3.04	0.864	0.4510582

Eggers' test does not indicate the presence of funnel plot asymmetry.

SSD studies

Forest plot

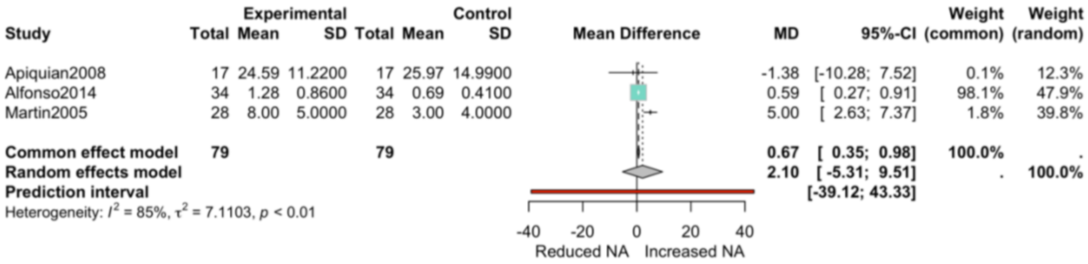

Figure 75

Number of studies: k = 3
Number of observations: o = 158 (o.e = 79, o.c = 79)

	MD	95%-CI	z|t	p-value
Common effect model	0.6664	[0.3492; 0.9835]	4.12	< 0.0001
Random effects model	2.1039	[-5.3063; 9.5142]	1.22	0.3463
Prediction interval		[-39.1191; 43.3270]		

Quantifying heterogeneity:
tau^2 = 7.1103 [0.6530; >100.0000]; tau = 2.6665 [0.8081; >10.0000]
I^2 = 84.9% [55.3%; 94.9%]; H = 2.57 [1.50; 4.43]

Test of heterogeneity:

Q	d.f.	p-value
13.25	2	0.0013

Details on meta-analytical method:
- Inverse variance method
- Restricted maximum-likelihood estimator for tau^2
- Q-Profile method for confidence interval of tau^2 and tau
- Hartung-Knapp adjustment for random effects model (df = 2)
- Prediction interval based on t-distribution (df = 1)

CHR-P studies

Forest plot

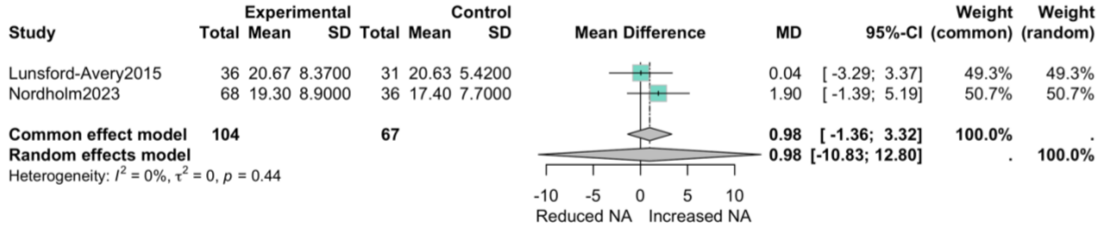

Figure 76

Number of studies: k = 2
Number of observations: o = 171 (o.e = 104, o.c = 67)

MD 95%-CI z|t p-value
Common effect model 0.9833 [-1.3572; 3.3239] 0.82 0.4103
Random effects model 0.9833 [-10.8322; 12.7989] 1.06 0.4822
Prediction interval

Quantifying heterogeneity:
tau^2 = 0; tau = 0; I^2 = 0.0%; H = 1.00

Test of heterogeneity:
Q d.f. p-value
0.61 1 0.4362

Details on meta-analytical method:
- Inverse variance method
- Restricted maximum-likelihood estimator for tau^2
- Hartung-Knapp adjustment for random effects model (df = 1)

Outliers and heterogeneity

Identified outliers (fixed-effect model)

"Martin2005"

Results with outliers removed

Review: NA

Number of studies: k = 4
Number of observations: o = 273 (o.e = 155, o.c = 118)

MD 95%-CI z|t p-value

Common effect model 0.5947 [0.2776; 0.9118] 3.68 0.0002
Random effects model 0.5947 [0.3124; 0.8771] 6.70 0.0068
Prediction interval [-0.1014; 1.2908]

Quantifying heterogeneity:
tau^2 = 0 [0.0000; 15.6073]; tau = 0 [0.0000; 3.9506]
I^2 = 0.0% [0.0%; 84.7%]; H = 1.00 [1.00; 2.56]

Test of heterogeneity:
Q d.f. p-value
0.90 3 0.8249

Details on meta-analytical method:
- Inverse variance method
- Restricted maximum-likelihood estimator for tau^2
- Q-Profile method for confidence interval of tau^2 and tau
- Hartung-Knapp adjustment for random effects model (df = 3)
- Prediction interval based on t-distribution (df = 2)

Identified outliers (random-effects model)

Results with outliers removed

Review: NA

Number of studies: k = 5
Number of observations: o = 329 (o.e = 183, o.c = 146)

MD 95%-CI z|t p-value
Common effect model 0.6721 [0.3578; 0.9864] 4.19 < 0.0001
Random effects model 1.6937 [-1.0841; 4.4716] 1.69 0.1657
Prediction interval [-5.1262; 8.5137]

Quantifying heterogeneity:
tau^2 = 3.4226 [0.2012; 41.9570]; tau = 1.8500 [0.4485; 6.4774]
I^2 = 71.3% [27.3%; 88.7%]; H = 1.87 [1.17; 2.97]

Test of heterogeneity:
Q d.f. p-value
13.92 4 0.0075

Details on meta-analytical method:
- Inverse variance method
- Restricted maximum-likelihood estimator for tau^2
- Q-Profile method for confidence interval of tau^2 and tau

- Hartung-Knapp adjustment for random effects model (df = 4)
- Prediction interval based on t-distribution (df = 3)

Influence analyses

Baujat plot

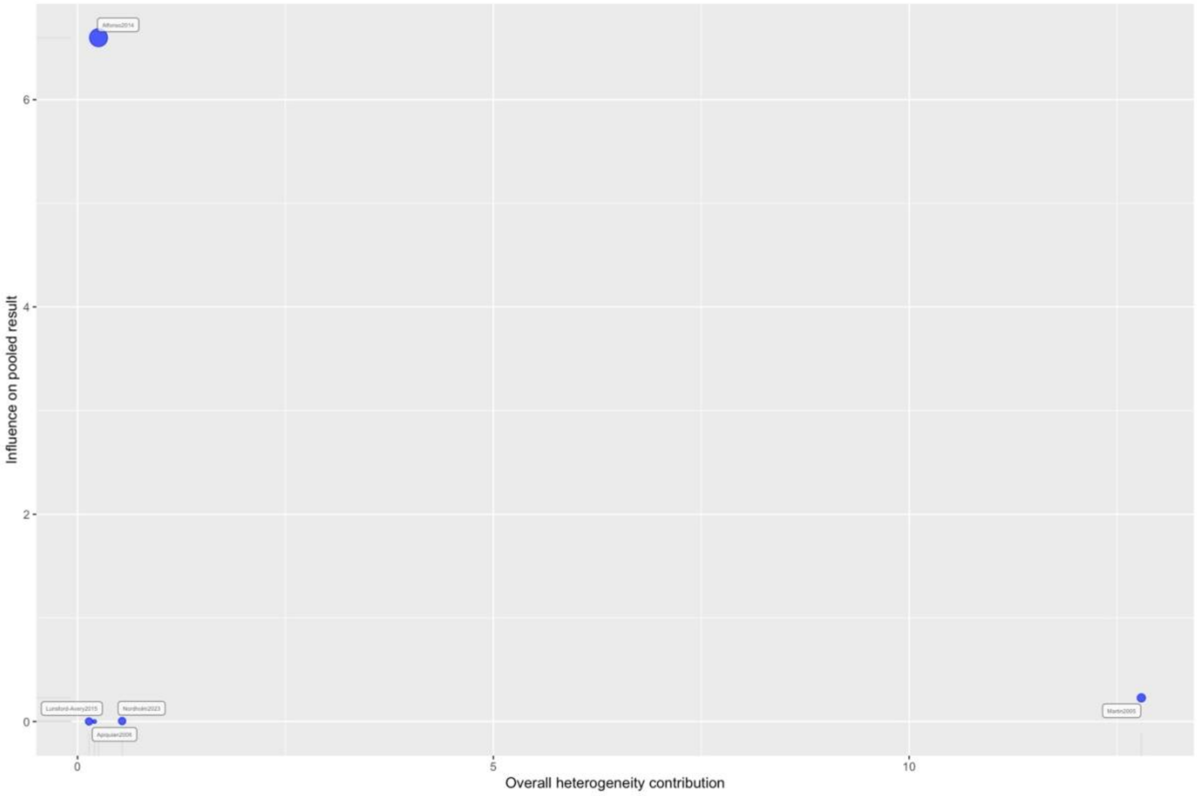

Figure 77

Influence diagnostics

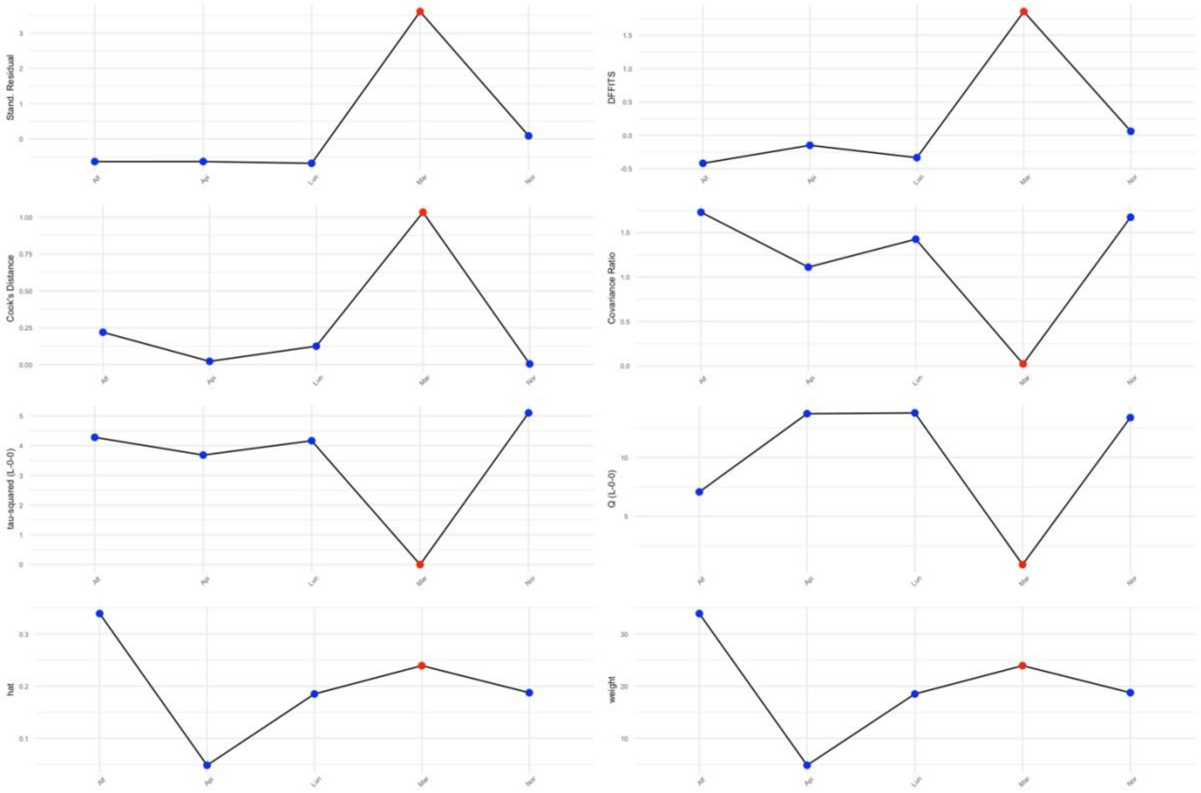

Figure 78

QUALITY OF SLEEP – SMD PSQI and AIS

Forest plot – all studies

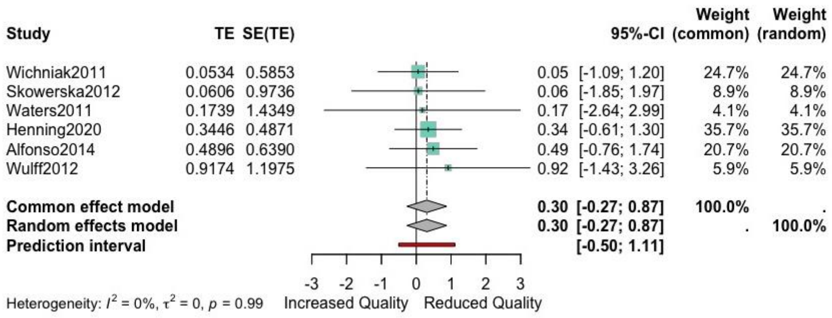

Figure 79

95%-CI %W(common) %W(random)
Alfonso2014 0.4896 [-0.7629; 1.7421] 20.7 20.7
Henning2020 0.3446 [-0.6101; 1.2993] 35.7 35.7
Skowerska2012 0.0606 [-1.8476; 1.9687] 8.9 8.9

Waters2011   0.1739 [-2.6383; 2.9862]   4.1   4.1
Wichniak2011 0.0534 [-1.0938; 1.2006]   24.7   24.7
Wulff2012   0.9174 [-1.4296; 3.2644]   5.9   5.9

Number of studies: k = 6

                                                 95%-CI   z p-value
Common effect model 0.3042 [-0.2659; 0.8742] 1.05 0.2957
Random effects model 0.3042 [-0.2659; 0.8742] 1.05 0.2957
Prediction interval   [-0.5034; 1.1117]

Quantifying heterogeneity:
 tau^2 = 0; tau = 0; I^2 = 0.0% [0.0%; 74.6%]; H = 1.00 [1.00; 1.99]

Test of heterogeneity:
    Q d.f. p-value
 0.61   5 0.9877

Details on meta-analytical method:
- Inverse variance method
- Restricted maximum-likelihood estimator for tau^2
- Prediction interval based on t-distribution (df = 4)

Funnel plot

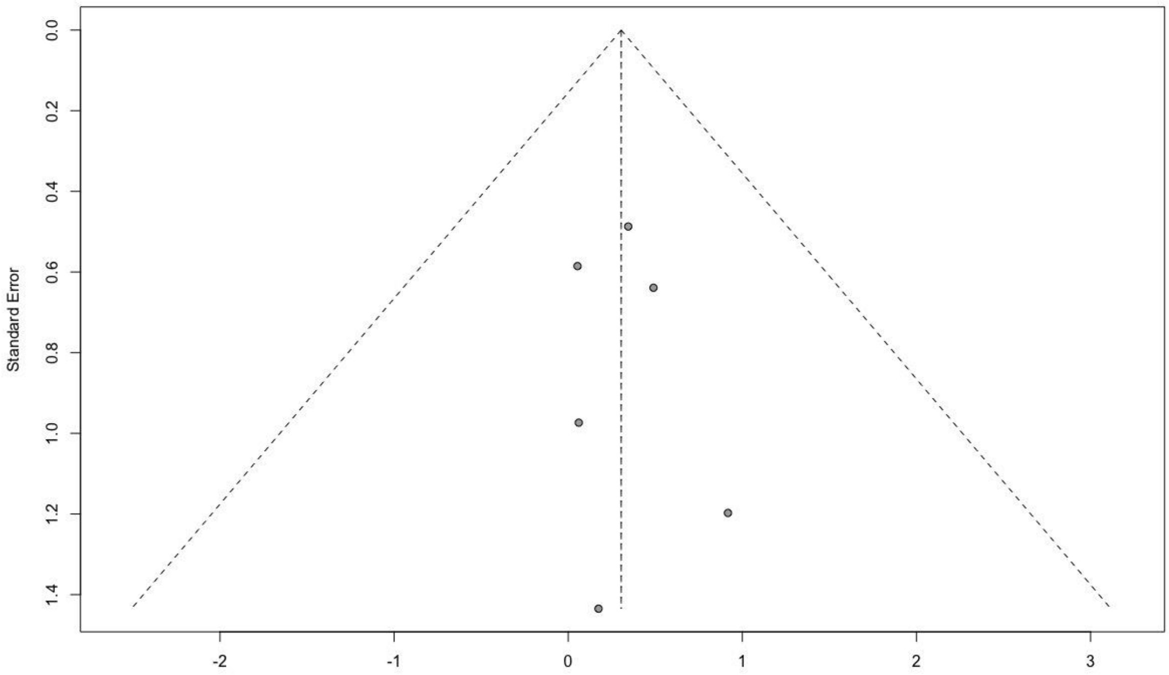

Figure 80

Eggers' test

intercept 95% CI t p
0.157 -0.72 - 1.03 0.351 0.7432214

Eggers' test does not indicate the presence of funnel plot asymmetry.

Baujat Plot

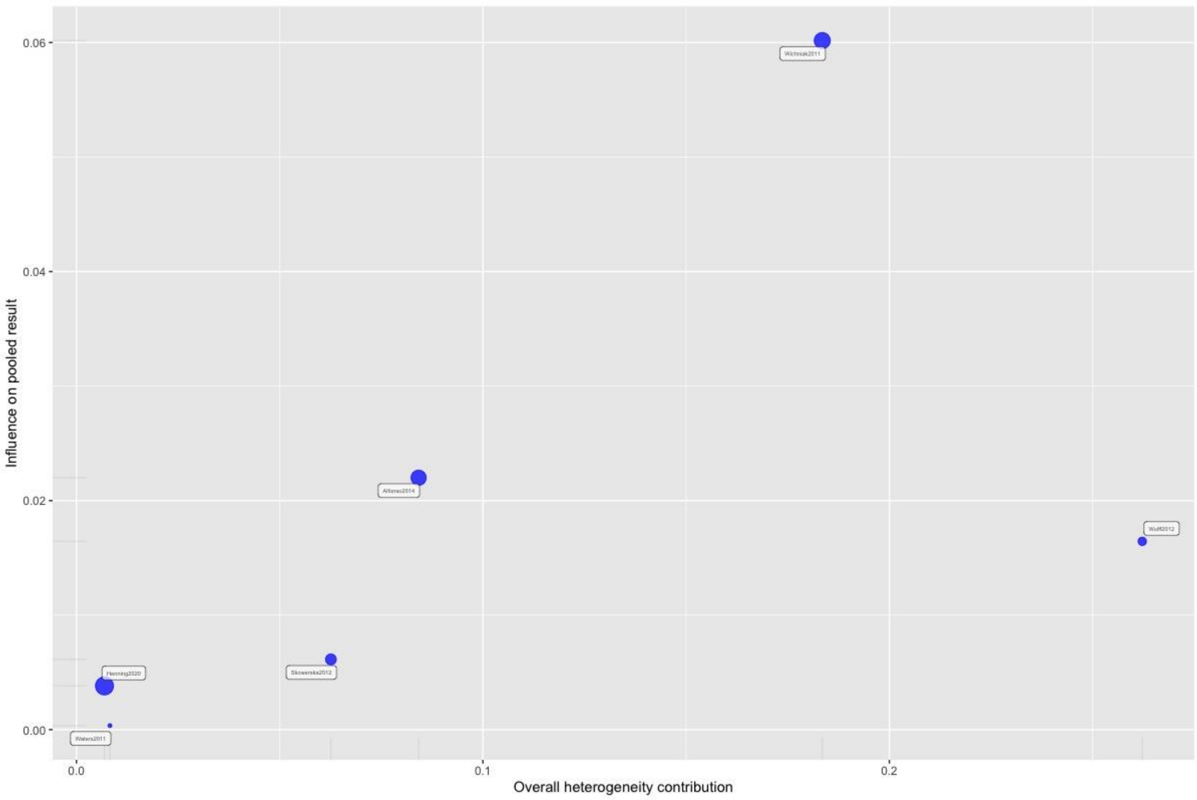

Figure 81

Influence analyses

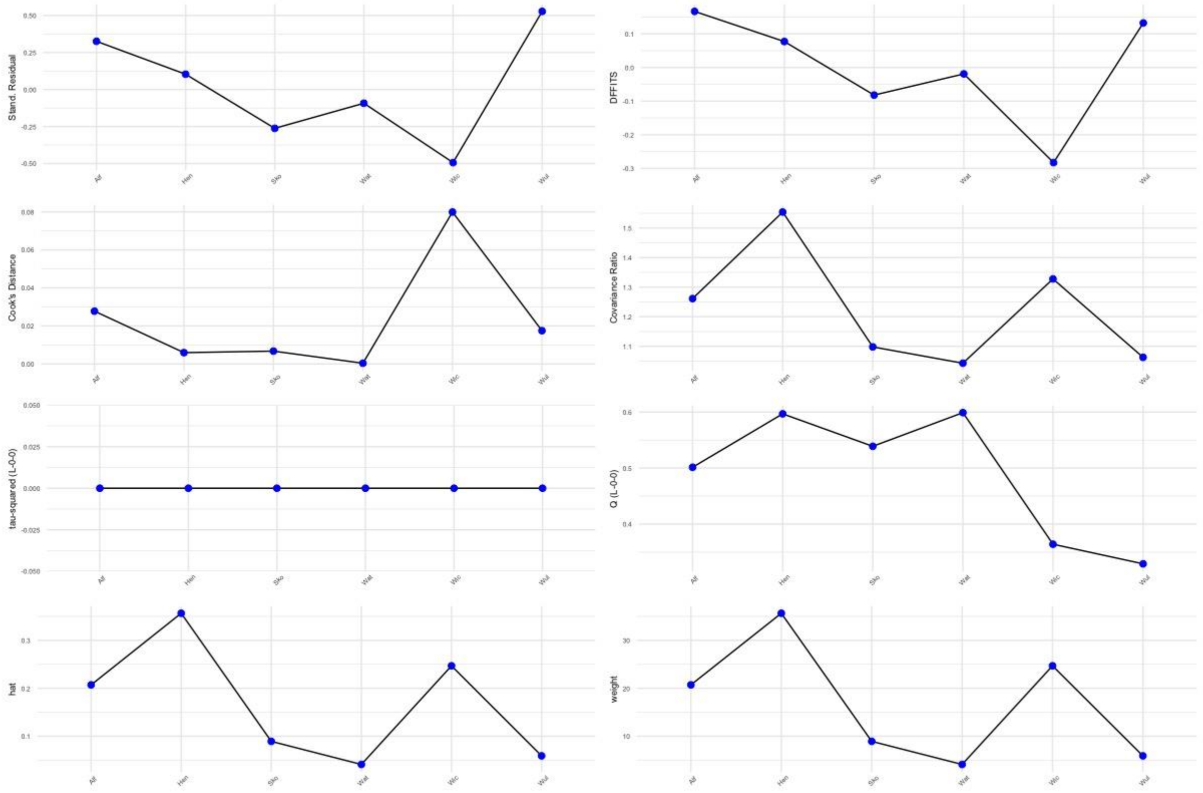

Figure 82

SSD studies

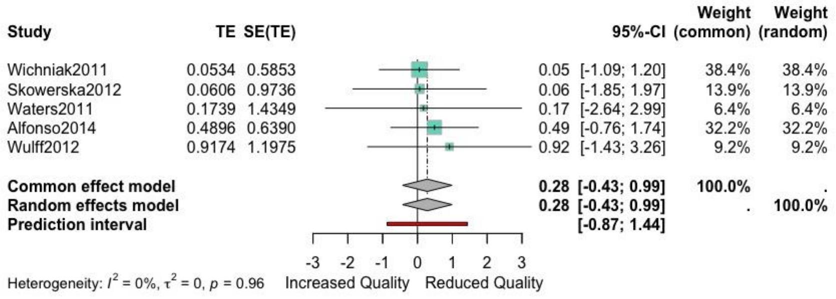

Figure 83

		95%-CI	%W(common)	%W(random)
Alfonso2014	0.4896	[-0.7629; 1.7421]	32.2	32.2
Skowerska2012	0.0606	[-1.8476; 1.9687]	13.9	13.9
Waters2011	0.1739	[-2.6383; 2.9862]	6.4	6.4
Wichniak2011	0.0534	[-1.0938; 1.2006]	38.4	38.4
Wulff2012	0.9174	[-1.4296; 3.2644]	9.2	9.2

Number of studies: k = 5

95%-CI z p-value

Common effect model 0.2818 [-0.4289; 0.9924] 0.78 0.4371
Random effects model 0.2818 [-0.4289; 0.9924] 0.78 0.4371
Prediction interval   [-0.8722; 1.4357]

Quantifying heterogeneity:
tau^2 = 0 [0.0000; 0.1555]; tau = 0 [0.0000; 0.3943]
I^2 = 0.0% [0.0%; 79.2%]; H = 1.00 [1.00; 2.19]

Test of heterogeneity:
Q d.f. p-value
0.60   4 0.9634

Details on meta-analytical method:
- Inverse variance method
- Restricted maximum-likelihood estimator for tau^2
- Q-Profile method for confidence interval of tau^2 and tau
- Prediction interval based on t-distribution (df = 3)

CHR-P

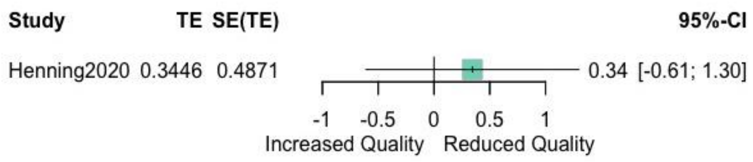

Figure 84

95%-CI   z p-value
Henning2020 0.3446 [-0.6101; 1.2993] 0.71 0.4793.

PRISMA 2020 Checklists

PRISMA 2020 Main Checklist

Topic	No.	Item	Location where item is reported
**TITLE**			
**Title**	1	Identify the report as a systematic review.	Page 1
**ABSTRACT**			

Topic	No.	Item	Location where item is reported
**Abstract**	2	See the PRISMA 2020 for Abstracts checklist	Page 2
**INTRODUCTION**			
**Rationale**	3	Describe the rationale for the review in the context of existing knowledge.	Pages 4 - 5
**Objectives**	4	Provide an explicit statement of the objective(s) or question(s) the review addresses.	Page 5
**METHODS**			
**Eligibility criteria**	5	Specify the inclusion and exclusion criteria for the review and how studies were grouped for the syntheses.	Pages 5-6
**Information sources**	6	Specify all databases, registers, websites, organisations, reference lists and other sources searched or consulted to identify studies. Specify the date when each source was last searched or consulted.	Page 5
**Search strategy**	7	Present the full search strategies for all databases, registers and websites, including any filters and limits used.	Page 5, Supplemental Materials
**Selection process**	8	Specify the methods used to decide whether a study met the inclusion criteria of the review, including how many reviewers screened each record and each report retrieved, whether they worked independently, and if applicable, details of automation tools used in the process.	Page 6
**Data collection process**	9	Specify the methods used to collect data from reports, including how many reviewers collected data from each report, whether they worked independently, any processes for obtaining or confirming data from study investigators, and if applicable, details of automation tools used in the process.	Pages 5-6
**Data items**	10a	List and define all outcomes for which data were sought. Specify whether all results that were compatible with each outcome domain in each study were sought (e.g. for all measures, time points, analyses), and if not, the methods used to decide which results to collect.	Pages 5-6
	10b	List and define all other variables for which data were sought (e.g. participant and intervention characteristics, funding sources). Describe any assumptions made about any missing or unclear information.	Pages 5-6

Topic	No.	Item	Location where item is reported
**Study risk of bias assessment**	11	Specify the methods used to assess risk of bias in the included studies, including details of the tool(s) used, how many reviewers assessed each study and whether they worked independently, and if applicable, details of automation tools used in the process.	Page 7
**Effect measures**	12	Specify for each outcome the effect measure(s) (e.g. risk ratio, mean difference) used in the synthesis or presentation of results.	Pages 5-7
**Synthesis methods**	13a	Describe the processes used to decide which studies were eligible for each synthesis (e.g. tabulating the study intervention characteristics and comparing against the planned groups for each synthesis (item 5)).	Pages 5-7
	13b	Describe any methods required to prepare the data for presentation or synthesis, such as handling of missing summary statistics, or data conversions.	Pages 5-7
	13c	Describe any methods used to tabulate or visually display results of individual studies and syntheses.	Pages 5-7
	13d	Describe any methods used to synthesize results and provide a rationale for the choice(s). If meta-analysis was performed, describe the model(s), method(s) to identify the presence and extent of statistical heterogeneity, and software package(s) used.	Pages 5-7
	13e	Describe any methods used to explore possible causes of heterogeneity among study results (e.g. subgroup analysis, meta-regression).	Pages 5-7
	13f	Describe any sensitivity analyses conducted to assess robustness of the synthesized results.	Pages 5-7
**Reporting bias assessment**	14	Describe any methods used to assess risk of bias due to missing results in a synthesis (arising from reporting biases).	Page 7
**Certainty assessment**	15	Describe any methods used to assess certainty (or confidence) in the body of evidence for an outcome.	Pages 5-7
**RESULTS**			
**Study selection**	16a	Describe the results of the search and selection process, from the number of records identified in the search to the number of studies included in the review, ideally using a flow diagram.	Page 8, Figure 1 - PRISMA 2020 flow diagram for systematic review

Topic	No.	Item	Location where item is reported
	16b	Cite studies that might appear to meet the inclusion criteria, but which were excluded, and explain why they were excluded.	Figure 1
**Study characteristics**	17	Cite each included study and present its characteristics.	Table 1 Supplemental Materials
**Risk of bias in studies**	18	Present assessments of risk of bias for each included study.	Page 8, Supplemental Materials
**Results of individual studies**	19	For all outcomes, present, for each study: (a) summary statistics for each group (where appropriate) and (b) an effect estimate and its precision (e.g. confidence/credible interval), ideally using structured tables or plots.	Pages 9-11, Figures 2a, 2b, 3a, 3b, 4a, 4b
**Results of syntheses**	20a	For each synthesis, briefly summarise the characteristics and risk of bias among contributing studies.	Pages 9-11, Figures 2a, 2b, 3a, 3b, 4a, 4b, Supplemental Materials
	20b	Present results of all statistical syntheses conducted. If meta-analysis was done, present for each the summary estimate and its precision (e.g. confidence/credible interval) and measures of statistical heterogeneity. If comparing groups, describe the direction of the effect.	Pages 9-11, Figures 2a, 2b, 3a, 3b, 4a, 4b, Supplemental Materials
	20c	Present results of all investigations of possible causes of heterogeneity among study results.	Pages 9-11, Figures 2a, 2b, 3a, 3b, 4a, 4b, Supplemental Materials
	20d	Present results of all sensitivity analyses conducted to assess the robustness of the synthesized results.	Pages 9-11, Figures 2a, 2b, 3a, 3b, 4a, 4b, Supplemental Materials
**Reporting biases**	21	Present assessments of risk of bias due to missing results (arising from reporting biases) for each synthesis assessed.	Supplemental Materials
**Certainty of evidence**	22	Present assessments of certainty (or confidence) in the body of evidence for each outcome assessed.	Supplemental Materials
**DISCUSSION**			
**Discussion**	23a	Provide a general interpretation of the results in the context of other evidence.	Pages 11-16
	23b	Discuss any limitations of the evidence included in the review.	Page 14

Topic	No.	Item	Location where item is reported
	23c	Discuss any limitations of the review processes used.	Page 14-15
	23d	Discuss implications of the results for practice, policy, and future research.	Pages 15-16
**OTHER INFORMATION**			
**Registration and protocol**	24a	Provide registration information for the review, including register name and registration number, or state that the review was not registered.	Page 5
	24b	Indicate where the review protocol can be accessed, or state that a protocol was not prepared.	Page 5
	24c	Describe and explain any amendments to information provided at registration or in the protocol.	None
**Support**	25	Describe sources of financial or non-financial support for the review, and the role of the funders or sponsors in the review.	17
**Competing interests**	26	Declare any competing interests of review authors.	Page 17
**Availability of data, code and other materials**	27	Report which of the following are publicly available and where they can be found: template data collection forms; data extracted from included studies; data used for all analyses; analytic code; any other materials used in the review.	Supplemental Materials

PRISMA Abstract Checklist

Topic	No.	Item	Reported?
**TITLE**			
**Title**	1	Identify the report as a systematic review.	Yes
**BACKGROUND**			
**Objectives**	2	Provide an explicit statement of the main objective(s) or question(s) the review addresses.	Yes
**METHODS**			
**Eligibility criteria**	3	Specify the inclusion and exclusion criteria for the review.	No

Topic	No.	Item	Reported?
**Information sources**	4	Specify the information sources (e.g. databases, registers) used to identify studies and the date when each was last searched.	Yes
**Risk of bias**	5	Specify the methods used to assess risk of bias in the included studies.	Yes
**Synthesis of results**	6	Specify the methods used to present and synthesize results.	Yes
**RESULTS**			
**Included studies**	7	Give the total number of included studies and participants and summarise relevant characteristics of studies.	Yes
**Synthesis of results**	8	Present results for main outcomes, preferably indicating the number of included studies and participants for each. If meta-analysis was done, report the summary estimate and confidence/credible interval. If comparing groups, indicate the direction of the effect (i.e. which group is favoured).	Yes
**DISCUSSION**			
**Limitations of evidence**	9	Provide a brief summary of the limitations of the evidence included in the review (e.g. study risk of bias, inconsistency and imprecision).	Yes
**Interpretation**	10	Provide a general interpretation of the results and important implications.	Yes
**OTHER**			
**Funding**	11	Specify the primary source of funding for the review.	Yes
**Registration**	12	Provide the register name and registration number.	No

From: Page MJ, McKenzie JE, Bossuyt PM, Boutron I, Hoffmann TC, Mulrow CD, et al. The PRISMA 2020 statement: an updated guideline for reporting systematic reviews. MetaArXiv. 2020, September 14. DOI: 10.31222/osf.io/v7gm2. For more information, visit: www.prisma-statement.org

Sensitivity analyses accounting for RoB (excluding studies with a high risk of bias)

Total sleep time

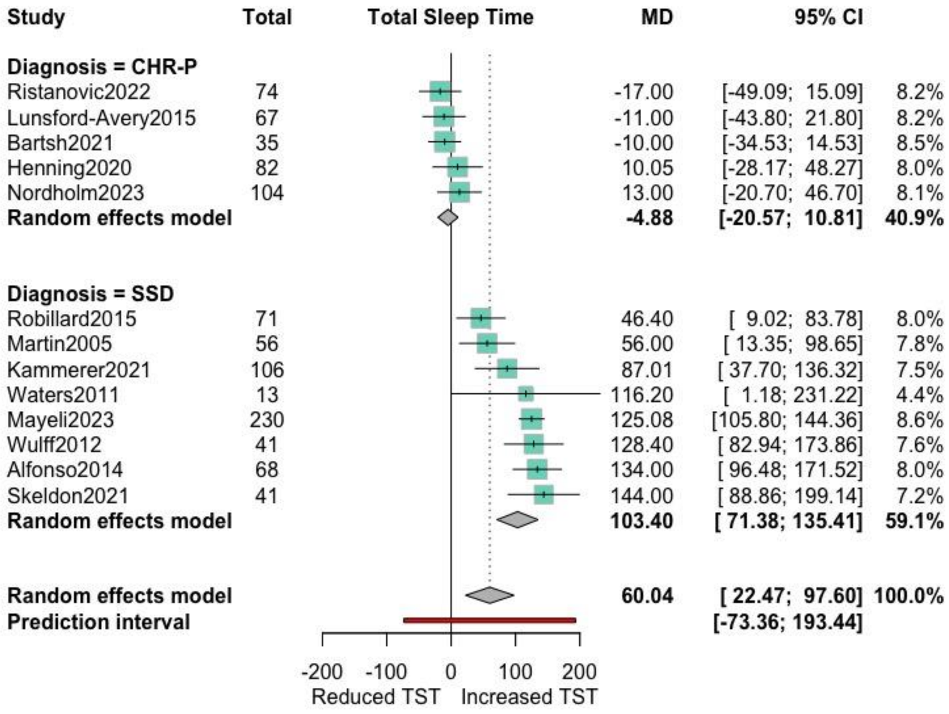

Figure 85

Review: Total Sleep time (TST) - Filtered

Number of studies: k = 13
Number of observations: o = 988 (o.e = 529, o.c = 459)

```
                  MD             95%-CI    t p-value
Random effects model 60.0375 [ 22.4701;  97.6049] 3.48  0.0045
Prediction interval          [-73.3627; 193.4378]
```

Quantifying heterogeneity (with 95%-CIs):
tau^2 = 3444.2049 [1531.1235; 9918.6617]; tau = 58.6873 [39.1296; 99.5925]
I^2 = 92.7% [89.2%; 95.0%]; H = 3.69 [3.05; 4.48]

Test of heterogeneity:

```
      Q d.f.  p-value
 163.82   12 < 0.0001
```

Results for subgroups (random effects model):

```
              k      MD             95%-CI    tau^2    tau     Q   I^2
Diagnosis = CHR-P  5  -4.8827 [-20.5731;  10.8078]        0        0  2.52  0.0%
Diagnosis = SSD    8 103.3953 [ 71.3772; 135.4134] 1046.4838 32.3494 24.11 71.0%
```

Test for subgroup differences (random effects model):

	Q	d.f.	p-value
Between groups	54.46	1	< 0.0001

Details of meta-analysis methods:
- Inverse variance method
- Restricted maximum-likelihood estimator for tau^2
- Q-Profile method for confidence interval of tau^2 and tau
- Calculation of I^2 based on Q
- Hartung-Knapp adjustment for random effects model (df = 12)
- Prediction interval based on t-distribution (df = 12)

Time in bed

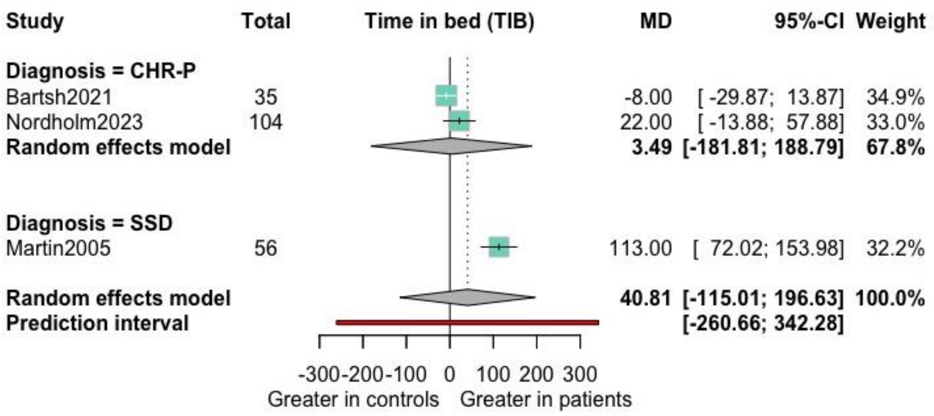

Figure 86

Review: TIB

Number of studies: k = 3
Number of observations: o = 195 (o.e = 115, o.c = 80)

	MD	95%-CI	t	p-value
Random effects model	40.8085	[-115.0084; 196.6254]	1.13	0.3768
Prediction interval		[-260.6638; 342.2808]		

Quantifying heterogeneity (with 95%-CIs):
tau^2 = 3608.3567 [751.4271; >36083.5671]; tau = 60.0696 [27.4122; >189.9568]
I^2 = 92.3% [80.8%; 96.9%]; H = 3.61 [2.28; 5.71]

Test of heterogeneity:

Q d.f. p-value
26.09 2 < 0.0001

Results for subgroups (random effects model):

	k	MD	95%-CI	tau^2	tau	Q	I^2
Diagnosis = CHR-P	2	3.4895	[-181.8106; 188.7896]	220.1494	14.8374	1.96	48.9%
Diagnosis = SSD	1	113.0000	[72.0195; 153.9805]	--	--	0.00	--

Test for subgroup differences (random effects model):

	Q	d.f.	p-value
Between groups	18.45	1	< 0.0001

Details of meta-analysis methods:
- Inverse variance method
- Restricted maximum-likelihood estimator for tau^2
- Q-Profile method for confidence interval of tau^2 and tau
- Calculation of I^2 based on Q
- Hartung-Knapp adjustment for random effects model (df = 2)
- Prediction interval based on t-distribution (df = 2)

Sleep Efficiency

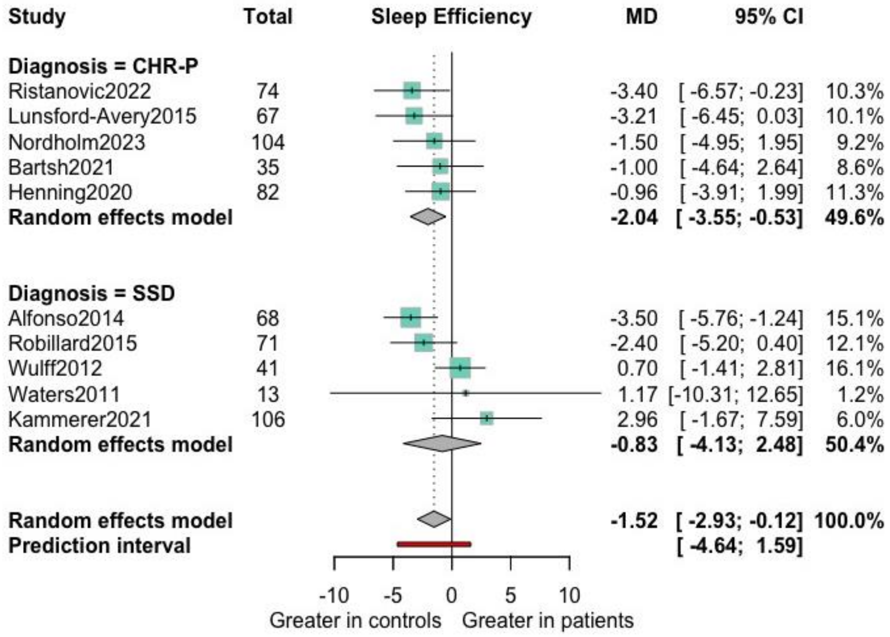

Figure 87

Review: Efficiency

Number of studies: k = 10
Number of observations: o = 661 (o.e = 359, o.c = 302)

MD 95%-CI t p-value

Random effects model -1.5228 [-2.9293; -0.1163] -2.45 0.0368
Prediction interval [-4.6385; 1.5929]

Quantifying heterogeneity (with 95%-CIs):
tau^2 = 1.4735 [0.0000; 10.7059]; tau = 1.2139 [0.0000; 3.2720]
I^2 = 35.7% [0.0%; 69.3%]; H = 1.25 [1.00; 1.81]

Test of heterogeneity:

Q	d.f.	p-value
13.99	9	0.1226

Results for subgroups (random effects model):

	k	MD	95%-CI	tau^2	tau	Q	I^2
Diagnosis = CHR-P	5	-2.0374	[-3.5468; -0.5281]	0	0	2.13	0.0%
Diagnosis = SSD	5	-0.8252	[-4.1296; 2.4793]	4.6345	2.1528	11.06	63.8%

Test for subgroup differences (random effects model):

	Q	d.f.	p-value
Between groups	0.86	1	0.3542

Details of meta-analysis methods:
- Inverse variance method
- Restricted maximum-likelihood estimator for tau^2
- Q-Profile method for confidence interval of tau^2 and tau
- Calculation of I^2 based on Q
- Hartung-Knapp adjustment for random effects model (df = 9)
- Prediction interval based on t-distribution (df = 9)

Sleep Latency

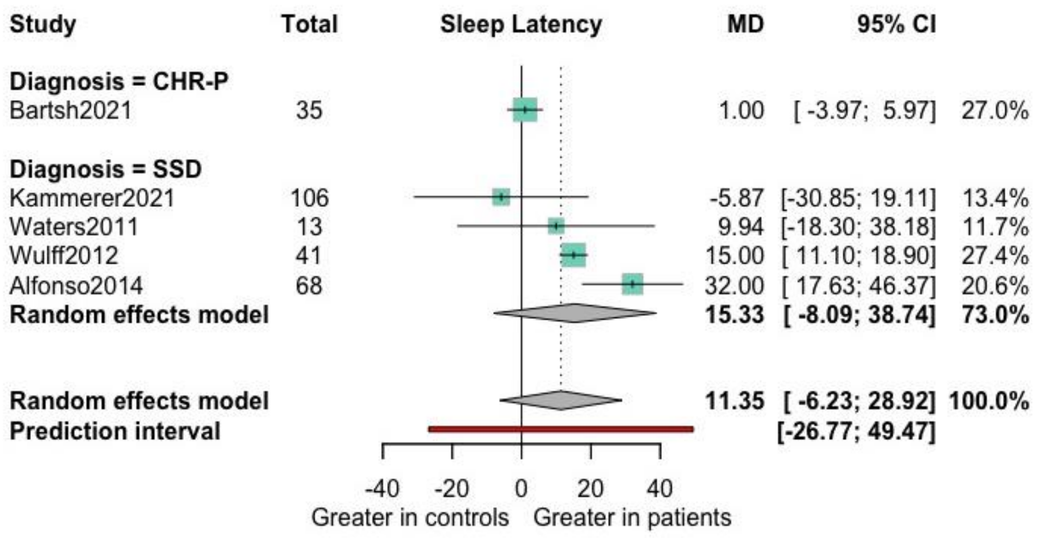

Figure 88

Review: latency

Number of studies: k = 5
Number of observations: o = 263 (o.e = 146, o.c = 117)

MD 95%-CI t p-value
Random effects model 11.3479 [-6.2271; 28.9228] 1.79 0.1475
Prediction interval [-26.7743; 49.4700]

Quantifying heterogeneity (with 95%-CIs):
tau^2 = 147.1323 [23.6023; >1471.3235]; tau = 12.1298 [4.8582; >38.3578]
I^2 = 86.4% [70.4%; 93.8%]; H = 2.71 [1.84; 4.00]

Test of heterogeneity:
Q d.f. p-value
29.39 4 < 0.0001

Results for subgroups (random effects model):
k MD 95%-CI tau^2 tau Q I^2
Diagnosis = CHR-P 1 1.0000 [-3.9703; 5.9703] -- -- 0.00 --
Diagnosis = SSD 4 **15.3285 [-8.0871; 38.7441]** 129.0279 11.3590 8.09 62.9%

Test for subgroup differences (random effects model):
Q d.f. p-value
Between groups 3.39 1 0.0656

Details of meta-analysis methods:
- Inverse variance method
- Restricted maximum-likelihood estimator for tau^2
- Q-Profile method for confidence interval of tau^2 and tau
- Calculation of I^2 based on Q
- Hartung-Knapp adjustment for random effects model (df = 4)
- Prediction interval based on t-distribution (df = 4)

Wakefulness After Sleep Onset

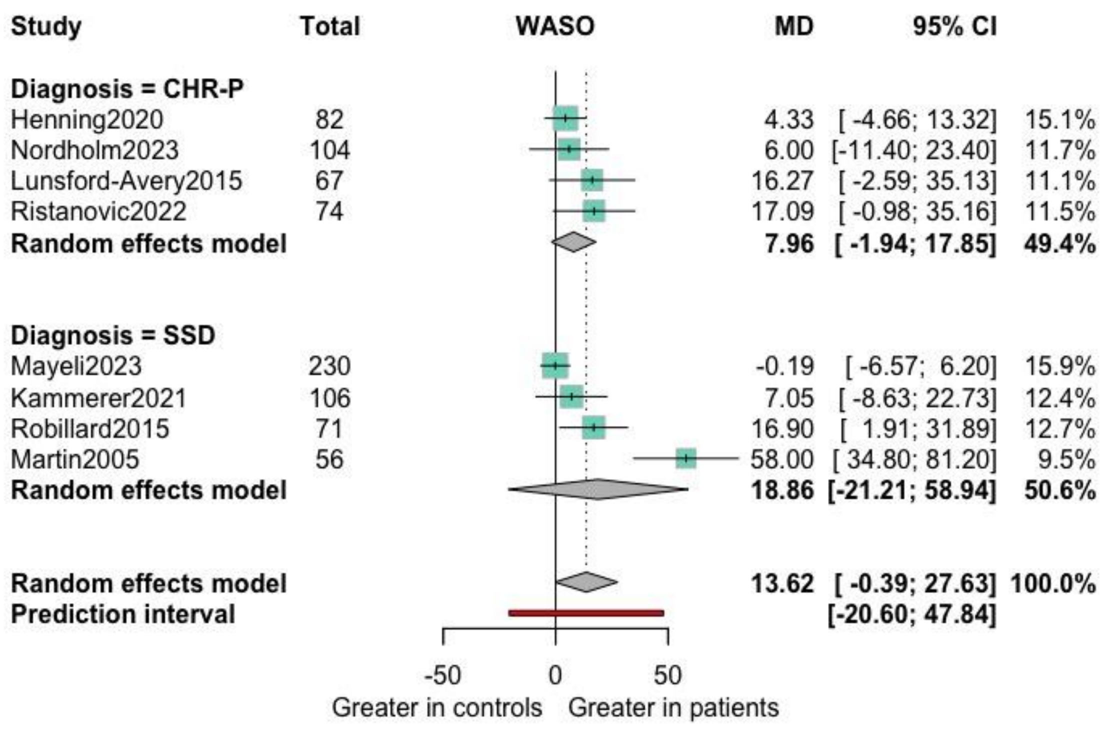

Figure 89

Review: WASO

Number of studies: k = 8
Number of observations: o = 790 (o.e = 430, o.c = 360)

	MD	95%-CI	t	p-value
Random effects model	13.6192	[-0.3904; 27.6288]	2.30	0.0551
Prediction interval		[-20.6002; 47.8385]		

Quantifying heterogeneity (with 95%-CIs):
tau^2 = 179.1711 [45.0586; 1273.0228]; tau = 13.3855 [6.7126; 35.6794]
I^2 = 74.6% [48.7%; 87.4%]; H = 1.98 [1.40; 2.82]

Test of heterogeneity:

     Q d.f. p-value
 27.57   7 0.0003

Results for subgroups (random effects model):

	k	MD	95%-CI	tau^2	tau	Q	I^2
Diagnosis = CHR-P	4	7.9574	[-1.9391; 17.8539]	0	0	2.40	0.0%
Diagnosis = SSD	4	18.8629	[-21.2140; 58.9398]	533.2640	23.0925	24.95	88.0%

Test for subgroup differences (random effects model):

	Q	d.f.	p-value
Between groups	0.71	1	0.4005

Details of meta-analysis methods:
- Inverse variance method
- Restricted maximum-likelihood estimator for tau^2
- Q-Profile method for confidence interval of tau^2 and tau
- Calculation of I^2 based on Q
- Hartung-Knapp adjustment for random effects model (df = 7)
- Prediction interval based on t-distribution (df = 7)

Nighttime awakenings

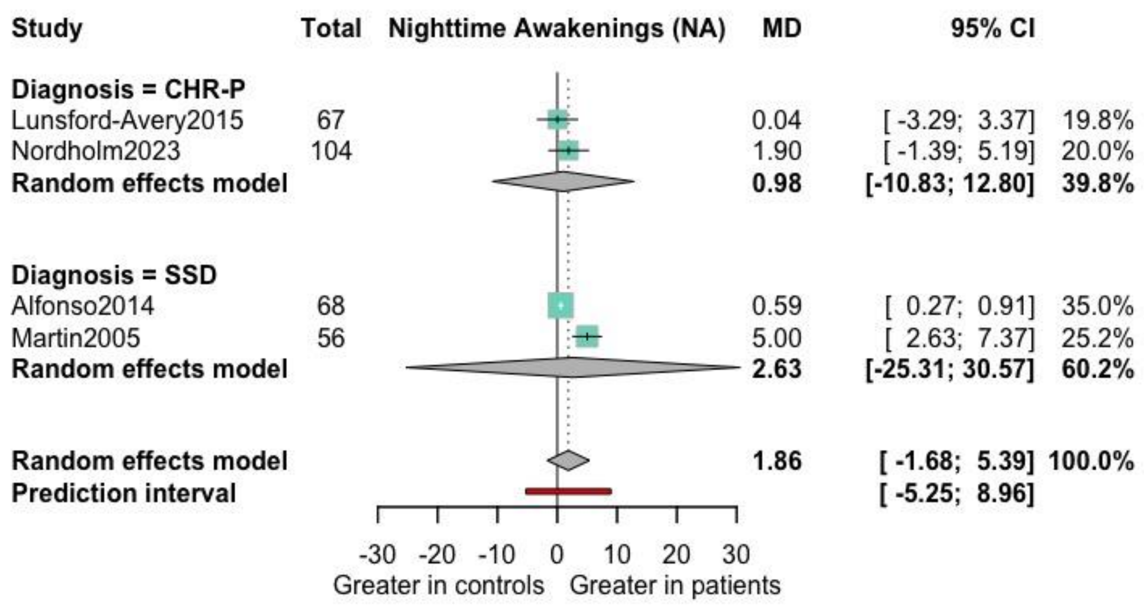

Figure 90

Review: NA

Number of studies: k = 4
Number of observations: o = 295 (o.e = 166, o.c = 129)

```
                    MD          95%-CI    t p-value
Random effects model 1.8563 [-1.6815; 5.3941] 1.67  0.1935
Prediction interval        [-5.2487; 8.9613]

Quantifying heterogeneity (with 95%-CIs):
 tau^2 = 3.6849 [0.3680; 66.8912]; tau = 1.9196 [0.6067; 8.1787]
 I^2 = 78.1% [41.0%; 91.9%]; H = 2.14 [1.30; 3.51]

Test of heterogeneity:
    Q d.f. p-value
 13.72    3  0.0033

Results for subgroups (random effects model):
                k    MD           95%-CI  tau^2    tau     Q   I^2
Diagnosis = CHR-P   2 0.9833 [-10.8322; 12.7989]      0      0  0.61  0.0%
Diagnosis = SSD     2 2.6320 [-25.3085; 30.5725] 8.9786 2.9964 13.04 92.3%

Test for subgroup differences (random effects model):
              Q d.f. p-value
Between groups 0.48    1  0.4899

Details of meta-analysis methods:
- Inverse variance method
- Restricted maximum-likelihood estimator for tau^2
- Q-Profile method for confidence interval of tau^2 and tau
- Calculation of I^2 based on Q
- Hartung-Knapp adjustment for random effects model (df = 3)
- Prediction interval based on t-distribution (df = 3)
```
